# Studies on the Genus *Pyrenopolyporus* (Hypoxylaceae) in Thailand Using a Polyphasic Taxonomic Approach

**DOI:** 10.3390/jof9040429

**Published:** 2023-03-30

**Authors:** Sarunyou Wongkanoun, Boonchuai Chainuwong, Noppol Kobmoo, Sittiruk Roytrakul, Sayanh Somrithipol, Jennifer Luangsa-ard, Esteban Charria-Girón, Prasert Srikitikulchai, Marc Stadler

**Affiliations:** 1 National Biobank of Thailand (NBT), National Science and Technology Development Agency (NSTDA), 111 Thailand Science Park, Phahonyothin Road, Khlong Nueng, Khlong Luang, Pathum Thani 12120, Thailand; sarunyou.won@nstda.or.th (S.W.);; 2 Plant Microbe Interaction Research Team (APMT), Integrative Crop Biotechnology and Management Research Group, National Center for Genetic Engineering and Biotechnology (BIOTEC), 113 Thailand Science Park, Phahonyothin Road, Khlong Nueng, Khlong Luang, Pathum Thani 12120, Thailand; noppol.kob@biotec.or.th (N.K.);; 3 Functional Proteomics Technology (IFPT), Functional Ingredients and Food Innovation Research Group, National Center for Genetic Engineering and Biotechnology (BIOTEC), 113 Thailand Science Park, Phahonyothin Road, Khlong Nueng, Khlong Luang, Pathum Thani 12120, Thailand; 4 Department of Microbial Drugs, Helmholtz Centre for Infection Research GmbH (HZI), and German Centre for Infection Research Association (DZIF), Partner Site Hannover-Braunschweig, Inhoffenstraße 7, 38124 Braunschweig, Germany; 5 Institute of Microbiology, Technische Universität Braunschweig, Spielmannstraße 7, 38106 Braunschweig, Germany

**Keywords:** Ascomycota, chemotaxonomy, five new species, molecular phylogeny, peptide mass fingerprint, Sordariomycetes

## Abstract

Over the past two decades, hypoxylaceous specimens were collected from several sites in Thailand. In this study, we examined their affinity to the genus *Pyrenopolyporus* using macroscopic and microscopic morphological characters, dereplication of their stromatal secondary metabolites using ultrahigh performance liquid chromatography coupled to diode array detection and ion mobility tandem mass spectrometry (UHPLC-DAD-IM-MS/MS), and molecular phylogenetic analyses. We describe and illustrate five novel species and a new record for the country, present multi-locus phylogenetic analyses that show the distinction between the proposed species, and provide proteomic profiles of the fungi using matrix associated laser desorption ionization-time-of-flight mass spectrometry (MALDI-TOF/MS) for the first time. Based on our findings, this strategy is useful as a complementary tool to distinguish species between *Daldinia* and *Pyrenopolyporus* in a consistent way with the phylogenetic analysis.

## 1. Introduction

**Figure 1:**
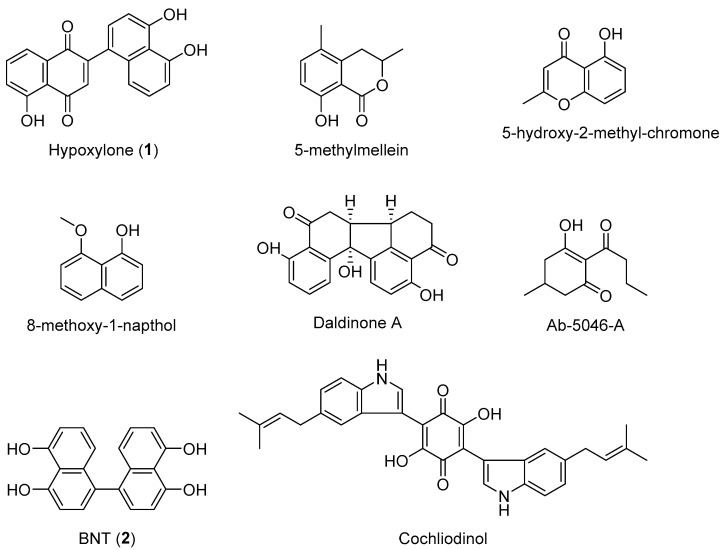
Chemical structures of stromatal metabolites detected in this study as well as representative metabolites from *Daldinia*, *Hypoxylon*, and other genera of the Hypoxylaceae as reported by Wendt et al. [3].

The genus *Pyrenopolyporus* was erected by Lloyd in 1917 [1] based on morphological characters of stromata, reminiscent of the macroscopic appearance of polyporaceous basidiomycetes. The earliest taxonomic name for its type species, *P. hunteri*, however, was *Penzigia polyporus* Starbäck. *Pyrenopolyporus hunteri* was previously treated as *Hypoxylon polyporum* by Ju and Rogers in their monograph of *Hypoxylon* [2]. The genus *Pyrenopolyporus* was resurrected by Wendt et al. [3] and included other species that were also previously placed in *Hypoxylon* by Ju and Rogers [2]. This group of hypoxylaceous pyrenomycetes had historically been regarded as an intermediate form between *Hypoxylon* and *Daldinia* (cf. [2,3,4]). However, the ITS-based phylogenies of the aforementioned studies did not provide conclusive evidence that would justify the separation of *Pyrenopolyporus* from *Hypoxylon* [5,6,7]. Their phylogeny was finally resolved by Wendt et al. [3] who demonstrated that three *Pyrenopolyporus* species constituted a distinct monophyletic clade as a sister group to *Daldinia*. Moreover, *Pyrenopolyporus* species are characterized by having massive, often discoid to peltate stromata forming long tubular perithecia. They differ from the species of *Daldinia*, of which the stromata possess no internal concentric zones such as in *D. korfii* [4] and *D. placentiformis* which have ascospores with indehiscent perispores in KOH solution [3]. Where this is known, the species of *Pyrenopolyporus* also differ from those of *Daldinia* in their anamorphic branching patterns and the production of certain secondary metabolites in their cultures [5] (Figure 1). *Pyrenopolyporus* spp. have a characteristic virgariella-like conidial stage and produce cochliodinol and 8-methoxy-1-naphtol but no chromones, eutypinols, and phytotoxic lactones of the “Ab-5046” type, which are characteristic of *Daldinia* [5]. The basis for corroborating the phylogenetic affinities of *Pyrenopolyporus* and allied genera has been recently established [3,8]. By using multi-locus phylogenetic studies of the type and authentic specimens of the stromatic Xylariales, a phylogenetic backbone for these pyrenomyceteous genera was provided for the first time. Likewise, phylogenomic studies of representatives of the Xylariales have further confirmed the placement of *Pyrenopolyporus* in the Hypoxylaceae and provided a starting point in the establishment of a stable phylogeny in the Xylariales [9]. The availability of high quality genomic data for representatives of this family has as well enabled the study of their biosynthetic diversity, revealing the presence of 783 different biosynthetic pathways across only 14 species, from which the majority of biosynthetic gene clusters had no clear links to the previously reported secondary metabolites from the Hypoxylaceae [10].

Recently, peptide mass fingerprint (PMF) created by matrix-assisted laser desorption/ionization time-of-flight mass spectrometry (MALDI-TOF/MS) has been widely used to support systematics and taxonomy (identification of microbial species and strains in medical mycology and bacteriology) [11,12]. This technique has emerged as an additional tool to identify isolates of filamentous fungi. During our taxonomic studies of Xylariales in Thailand, we discovered five new species of *Pyrenopolyporus* and a new record for the country. The current study is dedicated to their phenotypic description and illustration, and also provides evidence on their phylogenetic position. Furthermore, we have conducted, for the first time, a proteomics profiling via MALDI-TOF/MS for *Pyrenopolyporus*, which showed a resolution power to the species level, suggesting the use of this method as a complementary identification tool.

## 2. Materials and Methods

### 2.1. Survey and Sample Collection

The fungal specimens of this study were collected from several sampling sites in Thailand. Dark purple or dark grey, hemispherical or flattened, hard or velvety stromata occurring on dead fallen dicotyledonous wood and bamboos were carefully excised from the substrate and placed separately in paper bags and brought to the laboratory. Macroscopic features, including stromata appearance in the natural habitat, were examined using a Canon 60D digital camera (Canon Inc., Tokyo, Japan). Fungal cultures were obtained using a multi-spore isolation method accordance with Ju and Rogers [2]. Germinated ascospores were transferred to new agar plates. Pure cultures were deposited in Thailand Bioresource Research Center (TBRC, BCC) and National Biobank of Thailand (NBT), whereas the dried specimens were deposited at the BIOTEC Bangkok Herbarium (BBH). Scanning electron microscopy (SEM) was carried out using a conventional procedure described by Kuhnert et al. [13].

### 2.2. Morphological Characterization and HPLC Profiling

Morphological features, such as stromatal size and shape, perithecia, asci, apical apparatus, and ascospores were examined in accordance with Ju and Rogers [2] using a Nikon (Bangkok, Thailand) Eclipse Ni connected with a Nikon microscope camera DS-Ri2 and a stereo dissecting microscope Olympus SZ61 (Olympus, Bangkok, Thailand). Fungal cultures were grown on several media, i.e., Oatmeal Agar (Difco OA; Becton Dickinson, Carlsbad, CA, USA), Potato Dextrose Agar (Difco PDA), and Yeast Malt Glucose Agar (1% malt extract, 0.4% glucose and 0.4% yeast extract; agar 1%; YMGA). The morphological studies were carried out on 9 cm Petri dishes. Conidiogenous cells and conidiophore branching patterns of the anamorph were examined as proposed by Ju and Rogers [2]. Furthermore, the colors of stromata, KOH-extractable pigments, and cultures were documented following the color chart of Rayner [14]. For chemotaxonomic studies, stromatal secondary metabolites were extracted with acetone and analyzed using ultrahigh performance liquid chromatography coupled to diode array detection and ion mobility tandem mass spectrometry (UHPLC-DAD-IM-MS/MS) as described concurrently [15].

### 2.3. DNA Extraction, Polymerase Chain Reaction (PCR)

A modified method based on cetyltrimethyl ammonium bromide (CTAB) was used to isolate total genomic DNA from mycelia (pure cultures) grown for 5 days on PDA as previously described in Mongkolsamrit et al. [16]. The internal transcribed spacer regions (ITS), and partial sequences of the large subunit of the rDNA (LSU), RNA polymerase II (*RPB2*), and beta tubulin (*TUB2*) were amplified, using standard primers introduced by White at al. [17] (ITS4 and ITS5 for ITS [18], (LR5), Rehner and Samuels [19] (LROR) for LSU, Liu et al. [20] (RPB2–5F and 7Cr for *RPB2*), and O’Donnell and Cigelnik [21] (T1 and T22) for *TUB2*. PCR was conducted in 25 µL reaction volumes consisting of 1× PCR buffer, 200 μM of each of the four dNTPs, 2.5 mM MgCl_2_, 1 U Taq DNA Polymerase recombinant (Thermo Scientific, USA), 0.5 µM of each primer, and 50–100 ng of DNA template. The PCR conditions were performed as follows: 94 °C for 2 min, followed by 35 cycles of denaturation at 94 °C for 1 min, annealing at a suitable temperature for 1 min, extension at 72 °C for 2 min, and a final extension of 72 °C for 10 min. The annealing temperature of each gene was 55 °C for ITS and LSU; 54 °C for *RPB2*; and 53 °C for *TUB2*. PCR products were purified and subsequently sequenced with PCR amplification primers.

### 2.4. Sequencing Methods

A total of 5 µL of a post-PCR product was combined with 2 µL of ExoSAP-IT™ reagent for a 7 µL reaction total volume. When treating PCR product volumes greater than 5 µL, we simply increased the amount of ExoSAP-IT™ reagent proportionally. The mix was incubated at 37 °C for 15 min, followed by 15 min at 80 °C to degrade remaining primers and dinucleotides. DNA templates were processed for the DNA sequencing using the ABI-PRISM BigDye Terminator (version 3.1; Applied Biosystems, Foster, CA, USA) with both forward and reverse sequence-specific primers. Purified PCR products were used in a 20 µL sequencing reaction solution containing 8 µL of BigDye Terminator and 0.1 M of the same PCR primer. Sequencing reactions were performed using a 2 min initial denaturation at 96 °C, followed by 25 cycles for 10 s at 94 °C, 15 s at 50 °C, and 3 min at 60 °C. Sequence data were generated with the ABI PRISM 3100 DNA Analyzer (Applied Biosystems). Sequences were analyzed by Sequencer 3.1.1 software (Applied Biosystems) to compare variations. DNA sequences were checked manually and assembled using BioEdit v. 7.2.5 [22]. All newly generated sequences were submitted to GenBank (https://www.ncbi.nlm.nih.gov/ accessed on 21 December 2022) and listed in Table 1.

### 2.5. Phylogenetic Analyses

All sequences were aligned in Multiple Sequence Comparison by Log-Expectation program (MUSCLE) [37] and refined by direct examination. Multiple sequence alignments were analyzed with closely matched sequences and other reference taxa obtained from GenBank as shown in Table 1. Sequences were analyzed using maximum parsimony (MP), maximum likelihood (ML), and Bayesian inference (MB). The MP analysis was performed in PAUP*4.0b10 [38] and all characters were equally weighted, and gaps were treated as missing data. The most parsimonious trees were obtained from heuristic searches: 500 replicates of stepwise random addition and tree-bisection-reconnection (TBR) as a branch swapping algorithm.

Maximum parsimony bootstrap supports (MPBS) were estimated by 1000 replicates (10 replicates of stepwise random sequence addition). Tree length, consistency index (CI), retention index (RI), relative consistency index (RC), and homoplasy index (HI) were estimated. The ML tree and bootstrap analyses (MLBS) were conducted through the CIPRES Science Gateway V. 3.3 [39] using RAxML 8.2.4 [40] with the BFGS method to optimize GTR rate parameters. Bayesian posterior probabilities (BPP) of the branches were computed using MrBayes 3.0B4 [41] with the best-fit model (GTR + I + G), selected using the Akaike information criterion (AIC) in Mr Modeltest 2.2 [42] and tested with hierarchical likelihood ratios (hLRTs). Five million generations were run in four Markov chains and sampled every 100 generations with a burn-in value set at 5000 sampled trees. Sequences of *Graphostroma platystomum* CBS 270.87 and *Xylaria hypoxylon* CBS12260 were used as out groups. 

### 2.6. Cultivation of Fungal Strains for MALDI-TOF MS Analyses

The following strains were used for comparison in the MALDI-TOF/MS (for details see taxonomic part): *Pyrenopolyporus bambusicola* BCC89335; *P. cinereopigmentosus* BCC33615 and BCC89375; *P. hunteri* MUCL49209 (ex-epitype) and MUCL49339; *P. laminosus* BCC82043 and BCC89388; *P. macrosporus*: BCC89373; *P. papillatus* BCC20324 and BCC33622; *P. tonngachangensis* BCC31553 and BCC31555; *Daldinia flavogranulata*: BCC89367 and BCC82045; *D. bambusicola* BCC33677.

For the fermentation of *Pyrenopolyporus* and *Daldinia* spp., the seed culture were realized in 50 mL centrifuge tube containing 20 mL potato dextrose broth (Difco, PDB). Five pieces (ca. 20 mm) of a well grown agar plate of the fungi were used to inoculate each tube. The tubes were incubated for 3 days on a shaker (25 °C, under 12 h of fluorescent light at 150 rpm).

### 2.7. MALDI-TOF MS Analysis 

The fungal mycelia were mixed thoroughly with 300 µL distilled water, and with absolute ethanol (900 µL). The content was then centrifuged at 13,000 rpm for 5 min; the supernatant was discarded, and the pellet was air dried. Approximately 50 μL of the pellet was mixed thoroughly with 100 μL of trifluoroacetic acid (80%), and centrifuged at 13,000 rpm for 15 min. The protein concentration in the obtained supernatant was adjusted to 0.4–0.8 mg/μL with standard solvent (50% acetonitrile and 2.5% trifluoroacetic acid) and then 1 µL was placed on an MSP 96 target polished steel BC (Ref. 1011025092). Subsequently, eight sample positions (including one Bruker Bacterial Test Standard position) were overlaid with 1 µL of a matrix (HCCA portioned; Bruker Daltonics GmbH, Bremen, Germany) consisting of a saturated solution of α-cyano-4 hydroxycinnamic acid (HCCA) in 50% acetonitrile, 47.5% water, and 2.5% trifluoroacetic acid (final concentration:10 mg HCCA/mL) and air-dried at room temperature. MALDI-TOF/MS measurement was conducted on a Microflex LT bench-top instrument operated by FlexControl software (Bruker Daltonics GmbH, Bremen, Germany). Spectra were acquired in linear positive mode at a laser frequency of 200 Hz by using the standard FlexControl and AutoX methods within a mass range of 2000 to 20,000 Da. Spectra were accumulated in the MS/parent mode (240 shots) resulting in 24 MALDI spectra per strain.

Raw spectra from fungal extracts were loaded into the ClinProTools software (version 3; Bruker) and processed for analysis using the following parameters: 800 resolution, Top Hat baseline subtraction with a 10% minimal baseline width and no data reduction. Null spectra and noise spectra exclusion with a noise threshold of 2.00 were both enabled, and spectra grouping was also supported. Peak selection and average peak list calculation ranged from 2000 to 10,000 mass to charge ratio values (*m/z*), and recalibration was performed with a 1000 parts per million (ppm) maximal peak shift and 30% match to mass calibrant peaks. Non-recalibrated spectra were excluded. A final set of 82 peaks were retained. Mass to charge ratio values (*m/z*) from average spectra were identified according to their statistical significance, as determined by the different statistical tests realized in ClinProTools: ANOVA test and Wilcoxon/Kruskal–Wallis test (PWKW). Statistical analyses through principal component analysis (PCA) were performed using the obtained feature table containing the averaged peak areas/intensities values from the final set of 82 peaks. ClinProTools can also automatically select the two most discriminating peaks between classes of samples as defined by users. Therefore, the software picked the two most discriminating peaks between (1) all taxa, (2) *Pyrenopolyporus cinereopigmentosus* and *P. macrosporus*, (3) *P. hunteri, P. papillatus* and *P. tonngachangensis*, (4) *P. bambusicola* and *P. laminosus*. The ex-epitype species of the genus was included in each of these statistical analyses.

## 3. Results

### 3.1. Morphological Characterization

The morphological features of the five novel species and the new record of *Pyrenopolyporus* and the phylogenetic positions of these taxa according to the multi-locus genealogy are described further below.

**Figure 2:**
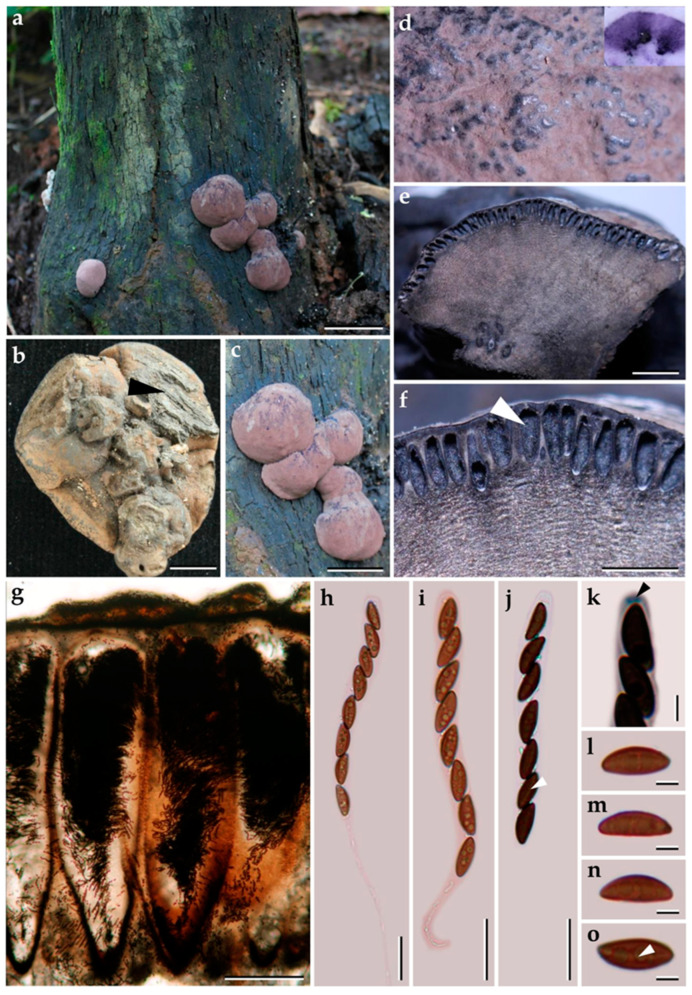
Morphological characteristics of *Pyrenopolyporus laminosus* from Thailand (BBH47928). (**a**,**c**) Stromata with natural substrate; (**b**) detail of stromal insertion point; (**d**) stromal surface and ostioles with KOH-extractable pigments in 10% KOH; (**e**) longitudinal section of the stroma showing perithecia and the tissue below the perithecial layer; (**f**) Perithecia (white arrow); (**g**) Perithecia under light microscope; (**h**–**j**) asci; (**k**) apical apparatus, bluing in Melzer’s reagent (black arrow); (**l**–**n**) ascospores. (**o**) Ascospore showing germ slit (white arrow); scale bars: (**a**,**c**) = 5 cm; (**b**) = 0.5 cm; (**e**) = 5 mm; (**f**) = 1 mm; (**g**) = 0.25 mm; (**h**–**j**) = 20 µm; (**k**–**o**) = 5 µm.

**Figure 3:**
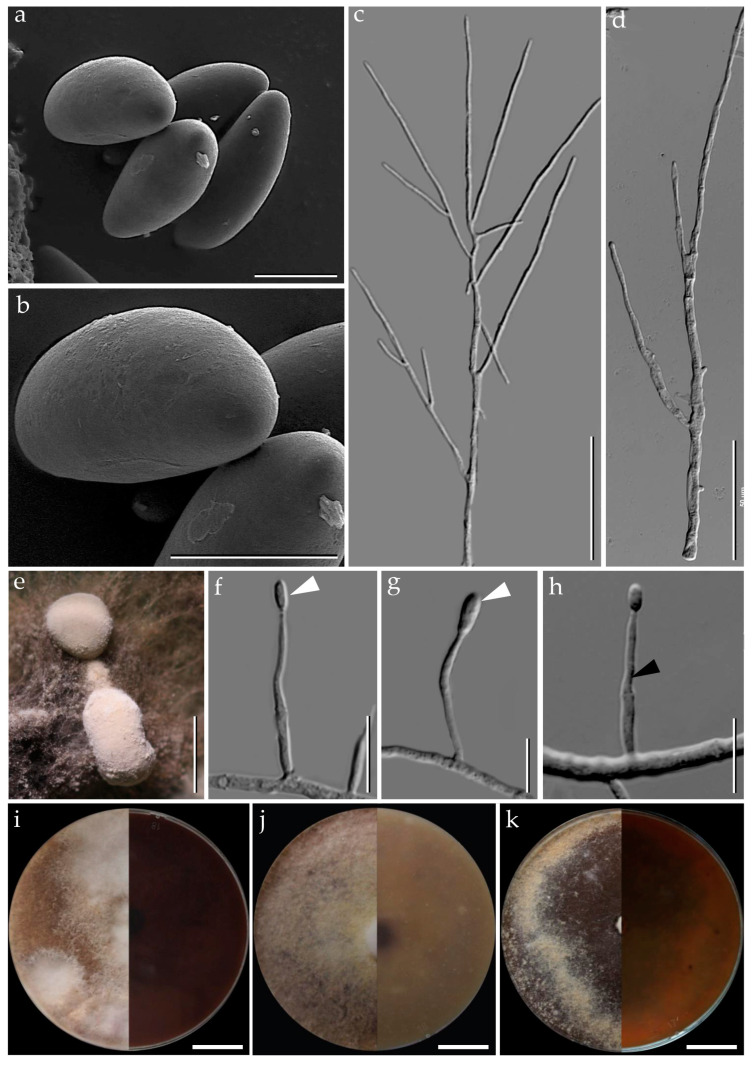
*Pyrenopolyporus laminosus* from Thailand (strain BCC89383). (**a**,**b**) Ascospores by SEM; (**c**,**d**) aerial mycelium showing branching pattern; (**e**) primordia in culture; (**f**) conidia (white arrows); (**h**) conidiogenous cell (black arrow); (**i**) colony on PDA after one month; (**j**) colony on OA after one month; (**k**) colony on YMGA after one month. Scale bars: (**a**,**b**) = 5 µm; (**c**,**d**) = 50 µm; (**e**) = 0.5 mm; (**f**–**h**) = 10 µm; (**i**–**k**) = 2 cm.

***Pyrenopolyporus laminosus*** (J. Fourn., Kuhnert and M. Stadler) M. Stadler, Kuhnert and L. Wendt, Mycological Progress 17 (1–2): 150 (2017).               Figure 2 and Figure 3.

*Material studied.* Thailand: Tak Province, Pa Daeng Mine’s area, 16°41′46″ N, 98°36′56″ E, reforestation forest, on decaying wood, 13 December 2017, P. Srikitikulchai (P.S.), S. Wongkanoun (S.W.), (BBH47928); (strain, BCC89383); DNA sequences of the Thai strain: (ITS = MN153855), (LSU = MN153872), (*RPB2* = MN172210), (*TUB2* = MN172199).

*Teleomorph. Stromata* solitary to coalescent, hemispherical to depressed-spherical, widely attached to the substrate, very rarely substipitate, smooth or with inconspicuous perithecial outlines, 18–39 × 14–24 mm; surface Mouse Grey (116), Purplish Grey (126), and Vinaceous Grey (126); dark brown granules immediately beneath the surface, with KOH-extractable pigments Livid Violet (79) or Greyish Lavender (98), often rather dilute, especially in fully mature to overly mature specimens; the tissue between perithecia greyish brown to brown, pithy to woody; the tissues below the perithecia layer greyish, soft-textured, with a blackish line separating the perithecial layer from the sterile internal tissue, interior blackish brown, soft-textured, solid, with a lamellate structure consisting of densely intricate small black and golden-brown lines, 8–14 mm thick. *Perithecia* lanceolate, 0.2–0.3 × 0.7–0.9 mm ($\overset{-}{x}$= 0.3 × 0.8 µm; *n* = 20). *Ostioles* umbilicate to slightly raised, discoid. *Asci* cylindrical, eight-spored, 170.0−207.5 µm in length, the spore-bearing parts, 75.0−87.5 µm long, 5.0−7.5 µm broad, the stipes, 87.5−137.5 µm long; with amyloid apical apparatus bluing in Melzer’s reagent, discoid, 1−2 µm high, 3 µm broad. *Ascospores* ellipsoid-inequilateral with narrowly rounded ends, (12–) 13–14 (–15) × 4–5 ($\overset{-}{x}$= 13.1 × 4.8 µm; *n*= 50); with straight to rarely slightly sigmoid germ slit covering much less than spore-length or nearly spore-length on the convex side; perispore indehiscent in 10% KOH, epispore smooth.

*Cultures and anamorph*. Colonies on OA covering a 9 cm Petri dish in 1 week, at first whitish becoming velvety to felty, azonate with entire margin, Rosy Vinaceous (58), reverse Olivaceous (46). Colonies on YMGA covering a 9 cm Petri dish in 1 week, azonate, at first aerial mycelium whitish becoming velvety to felty, azonate with entire margin, Olivaceous (48) and Dark Brick (66); reverse Sepia (63). Colonies on PDA covering a 9 cm Petri dish in 1 week, at first whitish becoming velvety to felty, azonate with entire margin, Dark Brick (66); reverse Dark Vinaceous (82), Sepia (63), and Dark Brick (66). *Primordia* cylindrical to somewhat clavate, unbranched or sometimes branched, 2.8 × 1.3 mm. *Conidiogenous structures* with virgariella-like branching patterns as defined by Ju and Rogers [2], main axis hyaline to pale brown, finely roughened. *Conidiogenous cells* produced holoblastically, cymbiform, obovoid, hyaline, 15–20 × 2–3 μm, each cell producing one or several conidia. *Conidia* hyaline, smooth, subglobose, obovoid, ellipsoid, (6–) 7–8 × 3–4 µm ($\overset{-}{x}$= 7.28 × 2.97 µm; *n* = 10).

*Additional specimens examined*. Thailand: Chiang Mai Province, Ban Hua Thung community forest, 18°51′17″ N, 99°16′57″ E, hill evergreen forest, on bamboo, 22 August 2016; P.S. and S.W., (BBH47916, BCC82043; BBH47917, BCC82044). Chiang Mai Province, Ban Hua Thung community forest, 18°51′17″ N, 99°16′57″ E, hill evergreen forest, on bamboo, 3 November 2016, P.S. and S.W., (BBH42275, BCC82671). Tak Province, Pa Daeng Mine’s area, 16°41′46″ N, 98°36′56″ E, restoration forest, on bamboo, 4 September 2018, P.S. and S.W., (BCC89388).

*Secondary metabolites*. Stromata contain hypoxylone (**1**), BNT (**2**), and an unknown hydroxy derivative of hypoyxlone (**3**: [M + H]^+^ = 349.07041 Da; C_20_H_12_O_6_, Figure S8) as major constituents (Figure S7 and Table S2).

*Notes*. The Thai specimens of *Pyrenopolyporus laminosus* correspond well to the description by Kuhnert et al. [28]. This species is distinctive for its stromatal morphology and the characteristic tissue below the perithecia layer is without any internal concentric zones. Herein we reexamined the type of material of *P. laminosus* (syn. *Hypoxylon laminosus*) and compared it with the Thai material, matching the data originally reported by Kuhnert et al. [28]. Our phylogeny based on multi-locus analyses showed that the Thai strains grouped with *Pyrenopolyporus laminosus* with high statistical supports MP, ML, and BPP, confirming that this species is not only present in the neotropics but also occurs in Thailand.

**Figure 4:**
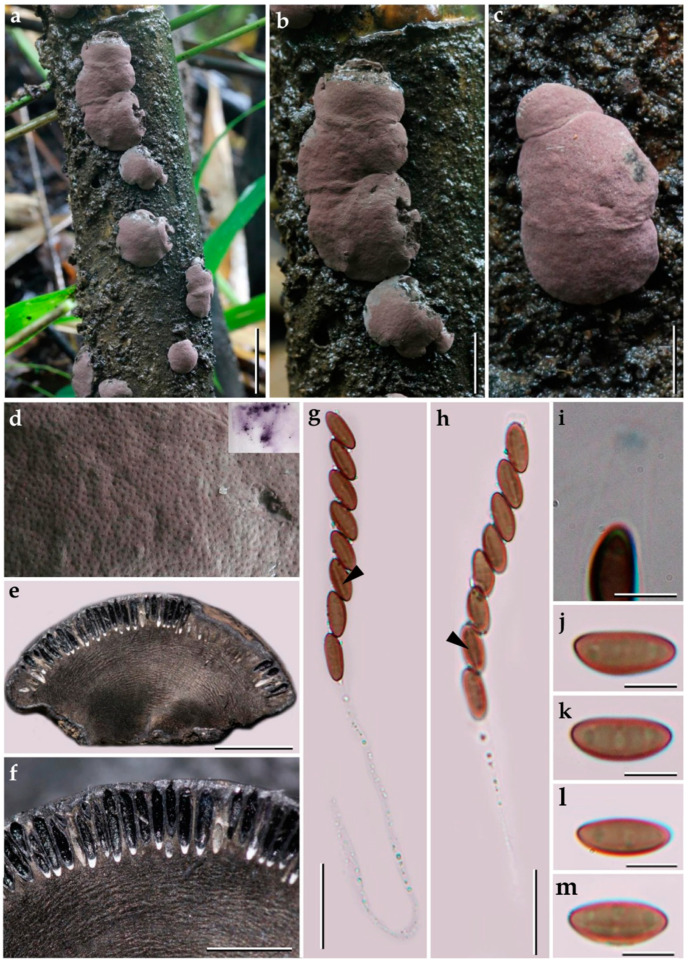
Morphological characteristics of *Pyrenopolyporus bambusicola* (BBH47923). (**a**–**c**) Stroma on bamboos; (**d**) stromal surface and ostioles with KOH-extractable pigments in 10% KOH; (**e**) longitudinal section of stroma showing perithecia and the tissue below the perithecial layer; (**f**) Perithecia; (**g**,**h**) asci showing germ slit (white arrows); (**i**) apical apparatus, bluing in Melzer’s reagent; (**j**–**m**) ascospores. Scale bars: (**a**) = 10 mm; (**b**,**c**) = 5 mm; (**e**) = 2 mm; (**f**) = 1 mm (**g**,**h**) = 20 µm; (**i**–**m**) = 5 µm.

**Figure 5:**
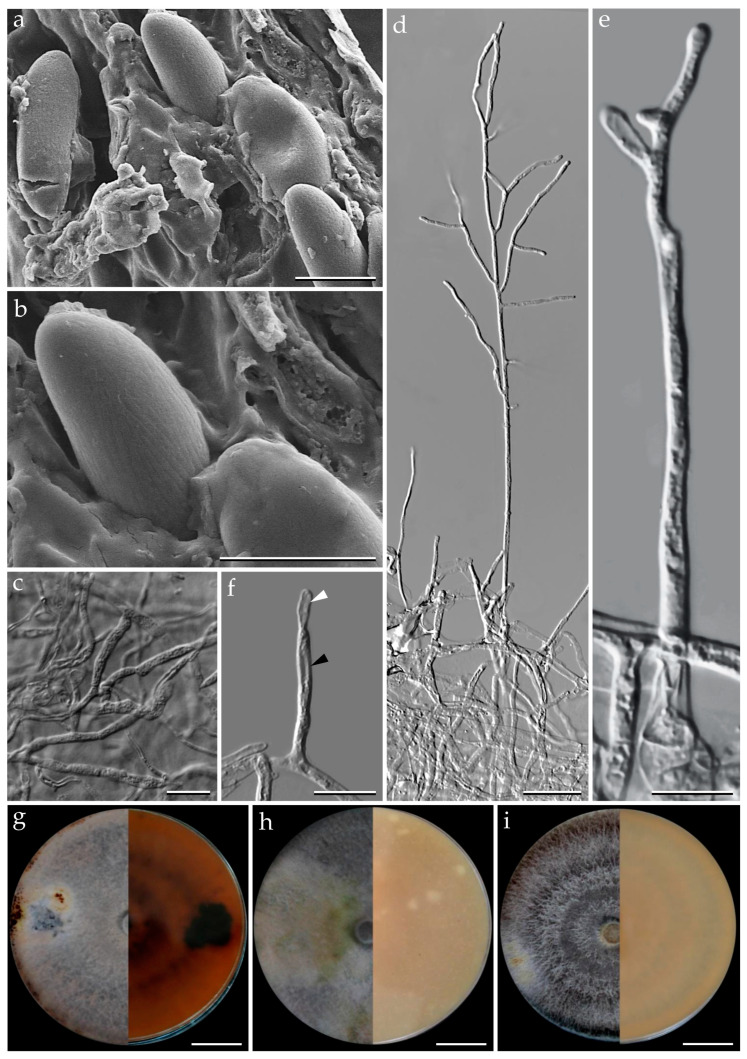
*Pyrenopolyporus bambusicola* strain BCC89369. (**a**,**b**) Ascospores by SEM; (**c**) vegetative mycelium; (**d**,**e**) aerial mycelium showing branching pattern; (**f**) conidia (white arrow) and conidiogenous cell (black arrow); (**g**) colony on PDA after one month; (**h**) colony on OA after one month; (**i**) colony on YMGA after one month. Scale bars: (**a**,**b**) = 5 µm; (**c**,**e**,**f**) = 10 µm; (**d**) = 20 µm; (**g**–**i**) = 2 cm.

***Pyrenopolyporus bambusicola*** Srikitikulchai, Wongkanoun, M. Stadler and Luangsa–ard, sp. nov.                                             Figure 4 and Figure 5.

*MycoBank*. MB846446.

*Etymology.*“*bambusicola*” refer to the bambusicolous habit.

*Holotype.* Thailand: Tak Province, Pa Daeng Mine’s area, 16°41′46″ N, 98°36′56″ E, restoration forest, on bamboo trunk in fire damaged area, 4 September 2018, P.S. and S.W., (BBH47923).

*Ex-type culture*. BCC89355. DNA sequences of ex-type culture: (ITS = OP304856), (LSU = OP304876), (*RPB2* = OP981624), (*TUB2* = OQ101839).

*Teleomorph. Stromata* solitary to coalescent, peltate to hemispherical with a short and broadly attached central base, the margin almost inseparable from host surface with the host surface, 11–16 mm long, 8–13 mm wide, 4–9 mm thick; surface Pale Mouse Grey (117) to Mouse Grey (116) and Pale Purplish Grey (127) with KOH-extractable pigments Livid Violet (79) and Greyish Lavender (98); dark brown to black tissue forming a thin layer above perithecia; the tissue between perithecia grey or blackish brown; the tissue below the perithecial layer without internal concentric zones, grey, 3–8 mm thick, with a lamellate structure consisting of densely intricate small black and golden brown lines; lacking the dark brown line below the perithecia layer. *Perithecia* tubular, 0.75–0.90 mm high, 0.30–0.35 mm broad. *Ostioles* umbilicate conspicuous. *Asci* cylindrical, very long-stipitate, eight-spored, 154–160 μm in length, the spore-bearing parts, 62–64 μm long, 4–5 μm broad; with amyloid apical apparatus, bluing in Melzer’s reagent, discoid in outline, 1.0–1.2 μm high, 1 μm broad. *Ascospores* brown to blackish brown, ellipsoid with narrowly rounded ends, 10–11 (–12) × (3–) 4–5 μm ($\overset{-}{x}$= 10.56 × 4.04 μm, *n* = 50), with a straight spore-length germ slit on the most convex side; perispore indehiscent in KOH, epispore smooth.

*Cultures and anamorph.* Colonies on OA covering a 9 cm Petri dish in 1 week, at first whitish becoming velvety to felty, azonate with entire margin, Herbage Green (17), reverse Olivaceous (48). Colonies on YMGA covering a 9 cm Petri dish in 1 week, at first whitish becoming velvety to felty, inconspicuous zonate with entire margin, Olivaceous (48) and Dark Brick (66) reverse Olivaceous (48). Colonies on PDA covering a 9 cm Petri dish in 1 week, at first whitish becoming velvety to felty, zonate with entire margin, Olivaceous (48); reverse Greyish Sepia (106) and Olivaceous Grey (121). *Conidiogenous structures* with virgariella-like branching patterns as defined by Ju and Rogers [2]. *Conidiogenous cells* cylindrical, hyaline, finely roughened, 14−15 × 2.5−3.0 µm. *Conidia* hyaline, smooth, ellipsoid, 5−6 × 3−4 µm.

*Additional specimens examined.* Thailand: Tak Province, Pa Daeng Mine’s area, 16°41′46″ N, 98°36′56″E, reforestation forest, on bamboo trunk (Bambusoideae) in fire damaged area, 4 September 2018, P.S. and S.W., (BCC89369, BBH47923; BCC89360).

*Secondary metabolites*. Stromata contain hypoxylone (**1**), BNT (**2**), and an unknown hydroxy derivative of hypoyxlone (**3**: [M + H]^+^ = 349.07041 Da; C_20_H_12_O_6_; Figure S8) as major constituents (Figures 18 and S7 and Table S2).

*Notes*. Our new fungus *Pyrenopolyporus bambusicola* showed a close relationship to *P. laminosus* which is associated with Bambusoideae but differs by the ascospore size range [10–11 (–12) × (3–) 4–5 (*P. bambusicola*) vs. 11–13.5 × 4.2–4.5 µm (*P. laminosus*)]. The tissue below the perithecial layer of *P. laminosus* has a blackish line separating the perithecial layer from the sterile internal tissue, a characteristic lacking in *P. bambusicola*. Additionally, the stromatal secondary metabolites found in *P. bambusicola* resemble the ones found in *P. laminosus*.

**Figure 6:**
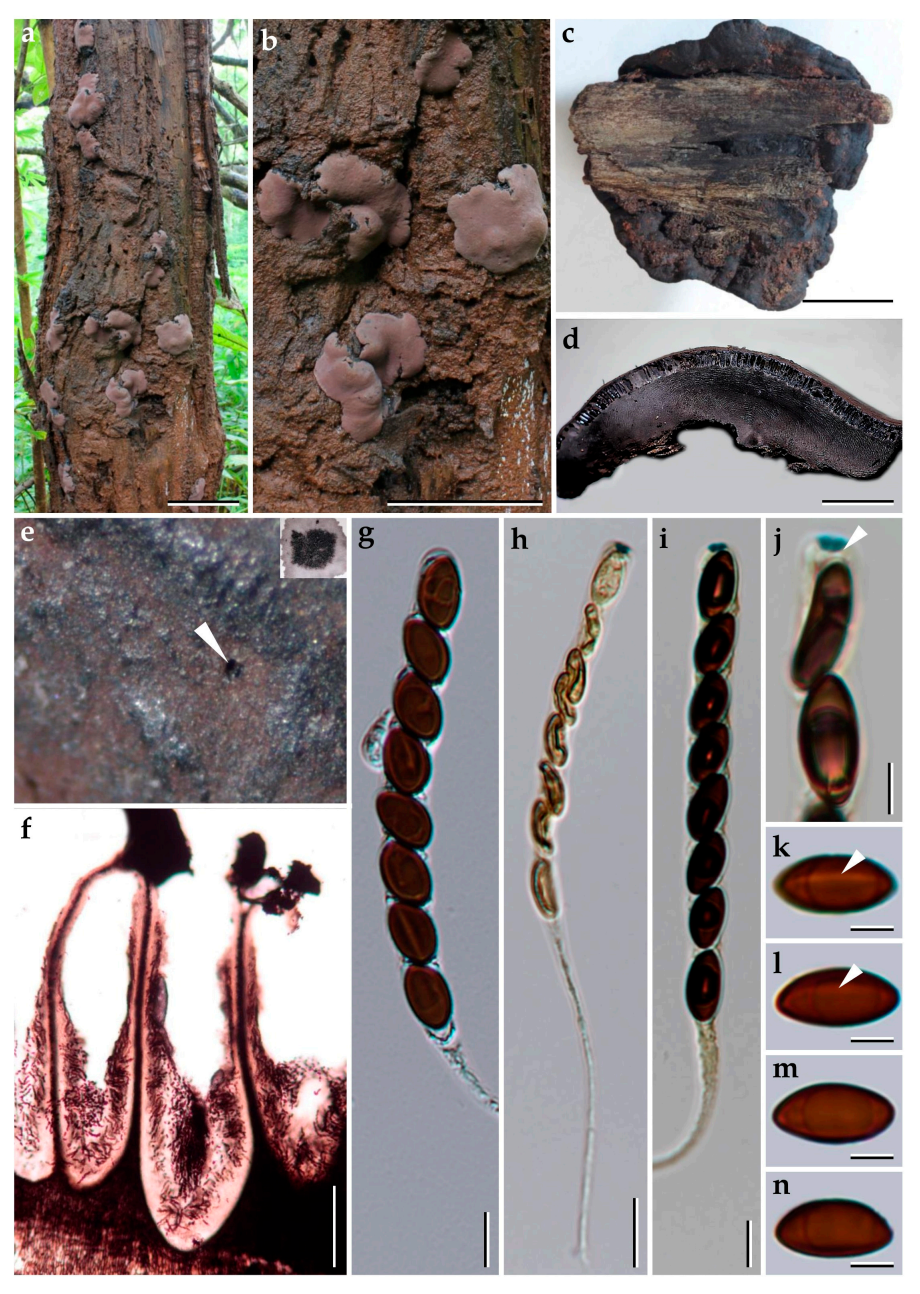
Morphological characteristics of *Pyrenopolyporus cinereopigmentosus* (BBH47927). (**a**,**b**) Stromata on natural habitat; (**c**) stromata showing ventral surface. (**d**) Longitudinal section of the stroma showing the tissue below the perithecial layer; (**e**) stromatal surface showing ostioles (white arrow) with KOH-extractable pigment; (**f**) longitudinal section of the stromata showing perithecia under light microscope; (**g**–**i**) asci. (**j**) Apical apparatus bluing in Melzer’s reagent (white arrow); (**k**,**l**) ascospores showing germ slit (white arrow); (**m**,**n**) ascospores. Scale bars (**a**,**b**) = 5 cm; (**c**) = 1 cm; (**d**) = 5 mm; (**f**) = 0.25 mm; (**g**–**l**) = 10 µm; (**j**–**n**) = 5 µm.

**Figure 7:**
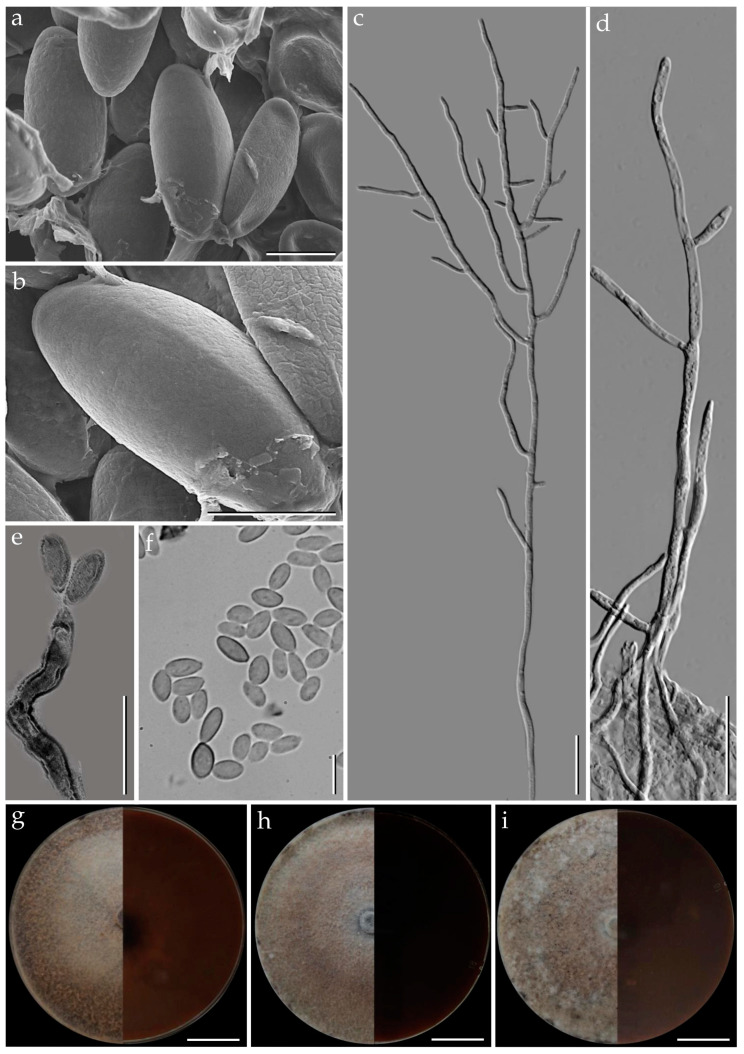
*Pyrenopolyporus cinereopigmentosus* strain BCC89382. (**a**,**b**) Ascospores by SEM; (**c**,**d**) aerial mycelium showing branching pattern; (**e**) conidiogenous cell; (**f**) conidia; (**g**) colony on PDA after one month; (**h**) colony on OA after one month; (**i**) colony on YMGA after on month. Scale bars: (**a**,**b**) = 5 µm; (**c**,**d**) = 20 µm; (**e**,**f**) = 10 µm; (**g**–**i**) = 2 cm.

***Pyrenopolyporus cinereopigmentosus*** Srikitikulchai, Wongkanoun, M. Stadler and Luangsa-ard, sp. nov.                                            Figure 6 and Figure 7.

*MycoBank.* MB846447.

*Etymology.* from the Latin “*cinereus*” in reference to its grey KOH-extractable pigments of the stromatal surface.

*Holotype.* Thailand: Tak Province, Pa Daeng Mine’s area, 16°41′46″ N, 98°36′56″ E, restoration forest, on decaying wood in fire-damaged area, 4 September 2018, P.S. and S.W., (BBH47927).

*Ex-type culture*. BCC89382. DNA sequences of ex-type culture: (ITS = OP304860), (LSU = OP304882), (*RPB2* = OP981627), (*TUB2* = OQ101843).

*Teleomorph. Stromata* solitary to coalescent, effused-pulvinate, attached on substrate, the margin almost in contact with the host surface, 25–36 mm diam, 5–19 mm thick; surface Pale Mouse Grey (117), Mouse Grey (116), and Fuscous Black (104); dark brown to blackish brown immediately beneath the stromatal surface, with KOH-extractable pigment Dark Mouse Grey (119) and Iron Grey (122); the tissue between perithecia Pale Olivaceous Grey (120) or Olivaceous Grey (121); the tissue below the perithecia layer massive, Olivaceous Grey (121) and or Olivaceous Black (108), 3.6–5 mm thick. *Perithecia* tubular 0.9–1.1 mm high, 0.3–0.4 mm broad. *Ostioles* inconspicuous, umbilicate. *Asci* cylindrical, eight-spored, 180–248 µm in length, the spore bearing part, 83–98 µm long, 7–8 µm broad; apical apparatus bluing in Melzer’s reagent rectangular shape, 3–4 × 1–2 µm. *Ascospores* dark brown to blackish brown, unicellular, ellipsoid with narrowly to broadly rounded ends, (12–) 13–14 (–15) × 6–7 µm ($\overset{-}{x}$ = 13.62 × 6.42 µm; *n* = 25) with straight germ slit covering full spore length on convex side, perispore indehiscent in 10% KOH.

*Cultures and anamorph*. Colonies on OA covering a 9 cm Petri dish in 1 week, at first whitish becoming velvety to felty, inconspicuous zonate with entire margin, Sepia (63) and Dark Brick (66); reverse Olivaceous (48), Dull Green (70), and Sepia (68). Colonies on YMGA covering a 9 cm Petri dish in 1 week, at first aerial mycelium whitish becoming velvety, azonate with entire margin, Sepia (68); reverse Sepia (68) and Dark Vinaceous (82). Colonies on PDA covering a 9 cm Petri dish in 1 week, at first whitish becoming velvety to felty, inconspicuous zonate with entire margin, Sepia (62); reverse Dark Vinaceous (82), Sepia (63), and Brown Vinaceous (84). *Conidiogenous structures* with virgariella-like branching patterns as defined in Ju and Rogers [2], main axis hyaline and the cell walls roughed or smooth, dark brown to blackish brown. *Conidiogenous cells* cylindrical, hyaline, finely roughened, 10−15 × 2−3 µm. *Conidia* hyaline, smooth, ellipsoid, 5−8 × 2−3 µm.

*Additional specimens examined*. Thailand: Tak Province, Pa Daeng Mine’s area, 16°41′46″ N, 98°36′56″ E, reforestation forest, on decaying wood in fire-damaged area, 4 September 2018, P.S. and S.W., (BCC89355, BBH47920; BCC89360).

*Secondary metabolites*. Stromata contain hypoxylone (**1**), BNT (**2**), two isobaric unknown compounds (**4**: [M + Na]^+^ = 258.10997 Da; C_13_H_17_NO_3_), and other unknown metabolites (**5**: [M + H]^+^ = 633.45371; C_30_H_60_N_6_O_8_) as major constituents (Figure S7 and Table S2).

*Notes.* Molecular phylogenetic assessment via a multi-locus supermatrix approach led to the placement of our new fungus *Pyrenopolyporus cinereopigmentosus* as a sister species to *P. macrosporus*. Morphologically, *P. cinereopigmentosus* closely resembles the above-mentioned species by having pale brown to dark brown ascospore color but differs by the ascospore morphology and size range. *Pyrenopolyporus macrosporus* produces a highly variable shape of ascospore as shown in the Figure 7i−o, while the ascospore length is much larger than *P. cinereopigmentosus* as follows [(14–) 16–17 × (6–) 7–8 vs. (12–) 13–14 (–15) × 6–7 µm]. *Pyrenopolyporus cinereopigmentosus* differs from *P. hunteri* in the KOH-extractable pigment and the ascospores size range is as follows (12–) 13–14 (–15) × 6–7 (*P. cinereopigmentosus*) vs. 11.5–14.0 × 5.0–5.5 µm (*P. hunteri*)]. Our phylogenetic multi-locus analysis showed that our new species is clearly separated from *P. hunteri* with high statistical support. Similarly, the chemical characterization of this new fungus showed the additional presence of unknown compounds not presence among the major constituents of *P. laminosus* and *P. bambusicola*. However, its secondary metabolite profile resembles the one obtained for *P. macrosporus*.

**Figure 8:**
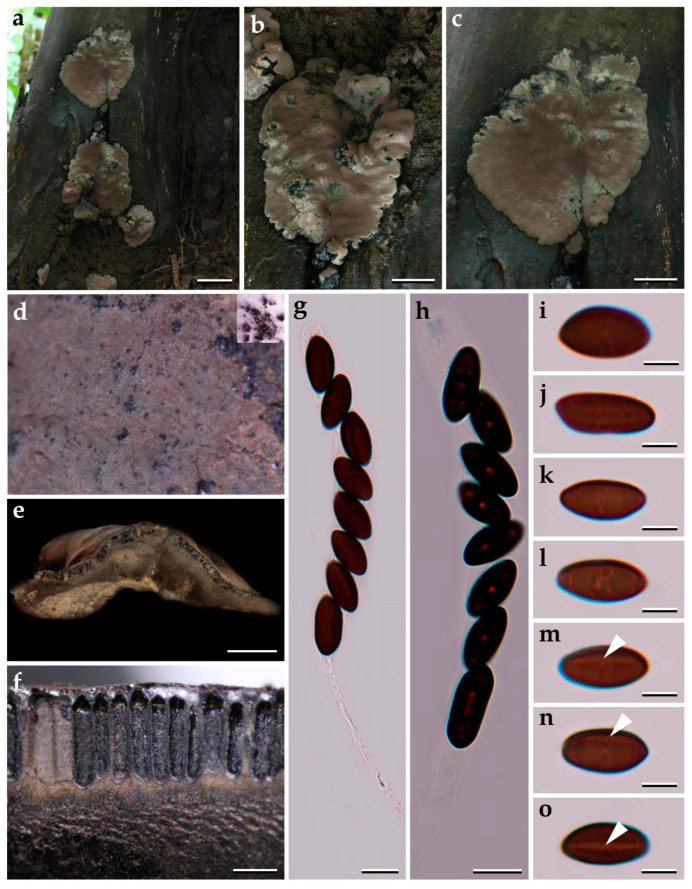
Morphological characteristics of *Pyrenopolyporus macrosporus* (BBH47924). (**a**–**c**) Stroma on natural habit; (**d**) stromal surface and ostioles with KOH-extractable pigments in 10% KOH; (**e**) longitudinal section of stroma showing the tissue below the perithecial layer; (**f**) Perithecia; (**g**) ascus in distilled water; (**h**) ascus in Melzer’s reagent showing apical apparatus; (**j**–**o**) ascospores with highly variable shapes. Scale bars: (**a**) = 2 cm; (**b**,**c**) = 1 cm; (**e**,**f**) = 0.5 mm; (**g**,**h**) = 10 µm; (**i**–**o**) = 5 µm.

**Figure 9:**
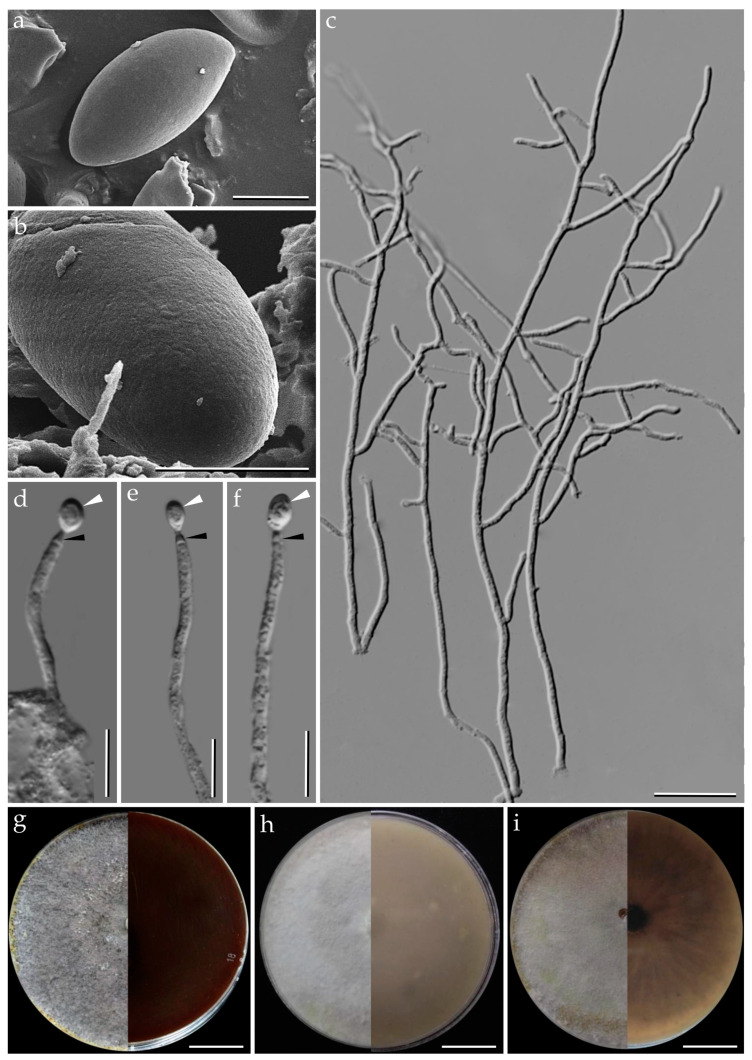
*Pyrenopolyporus macrosporus* strain (BCC89373). (**a**,**b**) Ascospores by SEM; (**c**) aerial mycelium showing branching pattern; (**d**–**f**) conidia (white arrow) and conidiogenous cells (black arrow); (**g**) colony on PDA after one month; (**h**) colony on OA after one month; (**i**) colony on YMGA after one month. Scale bars: (**a**,**b**) = 5 µm; (**c**) = 20 µm; (**d**–**f**) = 10 µm; (**g**–**i**) = 2 cm.

***Pyrenopolyporus macrosporus*** Srikitikulchai, Wongkanoun, M. Stadler and Luangsa-ard, sp. nov.                                             Figure 8 and Figure 9.

*MycoBank.* MB846448.

*Etymology.* “*macrosporus*” based on the large ascospores when compared with other *Pyrenopolyporus* species.

*Holotype.* Thailand: Tak Province, Pa Daeng Mine’s area, 16°41′46″ N, 98°36′56″ E, restoration forest, on decaying wood in fire-damaged area, 4 September 2018, P.S. and S.W., (BBH47924).

*Ex-type culture.* BCC89373; DNA sequences of ex-type culture: (ITS = OP304870), (LSU = OP304879), (*RPB2* = OP981621), (*TUB2* = OQ101844).

*Teleomorph. Stromata* solitary or coalescent, effused-pulvinate, attached on substrate, 20–70 mm long, 21–29 mm wide, 6–11 mm thick; surface Pale Olivaceous Grey (120), Olivaceous Grey (121), and Iron Grey (122); carbonaceous immediately beneath of the stromatal surface with KOH-extractable pigment Livid Violet (79) and Greyish Lavender (98); the tissue between perithecia greyish brown to brown or pithy to woody; with a woody line separating the perithecial layer from the sterile internal tissue; interior blackish brown or dark brown, 2.1–2.4 mm thick. *Perithecia* tubular, 0.7–0.9 mm high, 0.3–0.4 mm broad. *Ostioles* lower than the stromatal surface, inconspicuous. *Asci* unitunicate, cylindrical, eight-spored, 177–206 µm in length; the spore bearing part, 91–105 µm long, 7–8 µm broad; apical apparatus bluing in Melzer’s reagent, rectangular shape, 2 × 1 µm. *Ascospores* dark brown to blackish brown, unicellular, ellipsoid, inequilateral, highly variable with narrowly to broadly rounded ends, (14–) 16–17 × (6–) 7–8 µm ($\overset{-}{x}$ = 15.67 × 7.14 µm, *n* = 25) with straight germ slit covering full spore length on convex side, perispore indehiscent in 10% KOH, epispore smooth.

*Cultures and anamorph.* Colonies on OA covering a 9 cm Petri dish in 1 week, inconspicuous zonate with entire margin, at first whitish becoming Sepia (63) and Dark Brick (66); reverse Olivaceous (48), Dull Green (70), and Sepia (68). Colonies on covering a 9 cm Petri dish in 1 week, at first whitish becoming velvety to felty, zonate with entire margin, Sepia (68); reverse, Sepia (68), and Dark Vinaceous (82). Colonies on PDA covering a 9 cm Petri dish in 1 week, at first aerial mycelium whitish becoming velvety to felty, inconspicuous zonate with entire margin, Sepia (62); reverse Dark Vinaceous (82), Sepia (63), and Brown Vinaceous (84). *Conidiogenous structures* with virgariella-like branching patterns as defined in Ju and Rogers [2], main axis hyaline to hyaline to pale brown, finely roughened. *Conidiogenous cells* cylindrical, hyaline, finely roughened, 1−2 × 1−1.5 µm. *Conidia* hyaline, smooth, subglobose, 4−5 × 2.5−4.0 µm.

*Additional specimens examined.* Thailand: Tak Province, Pa Daeng Mine’s area, 16°41′46″ N, 98°36′56″ E, reforestation forest, on decaying wood in fire-damaged area, 4 September 2018, P.S. and S.W., (BCC89374, BBH47925).

*Secondary metabolites.* Stromata contain hypoxylone (**1**), BNT (**2**), two isobaric unknown compounds (**4**: [M + Na]^+^ = 258.10997 Da; C_13_H_17_NO_3_), and other unknown metabolite (**5**: [M + H]^+^ = 633.45371; C_30_H_60_N_6_O_8_) as major constituents (Figure S7 and Table S2).

*Notes. Pyrenopolyporus macrosporus* is clearly distinct from other members of the genus based on the phylogenetic placement as well as its morphological features. *Pyrenopolyporus macrosporus* is very similar to *P. symphyon* by having the stromatal surface with KOH-extractable purple color, the ascospores with narrowly to broadly rounded ends. However, *P. macrosporus* differs from *P. symphyon* by having larger ascospores [(14–) 16–17 × (6–) 7–8 *(P. macrosporus*) vs. 9.5–12 (−13) × 4–5 µm *(P. symphyon)*]. The original description of the type specimen shows tubular perithecia, 1.3 mm long, 0.3−0.4 mm broad, with dark brown, ovoid ascospores, 10 × 4–5 µm [43]. *Pyrenopolyporus macrosporus* differs from the previous descriptions of *P. symphyon* by Ju and Rogers [2] and Möller [43], and is confirmed as a new member of the genus.

**Figure 10:**
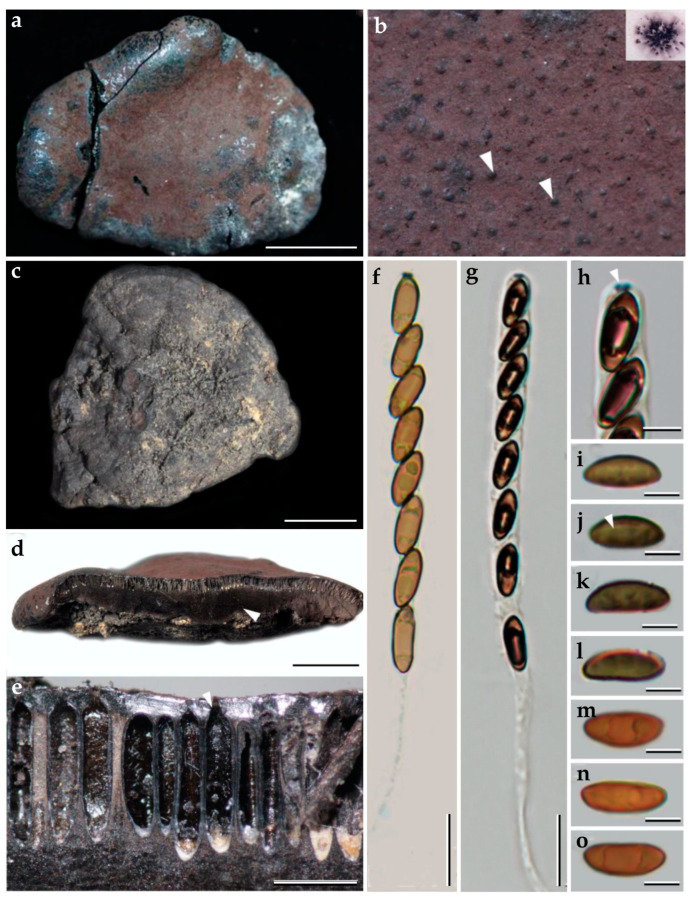
Morphological characteristics of *Pyrenopolyporus papillatus* (BBH15197). (**a**) Stroma; (**b**) stromatal surface showing ostiole (white arrows) and KOH-extractable pigment; (**c**) detail of stromal insertion point; (**d**) longitudinal section showing the tissue below the perithecial layer (arrow); (**e**) Perithecia (arrow); (**f**,**g**) asci in Melzer’s iodine regent; (**g**) apical apparatus bluing in Melzer’s reagent (arrow); (**i**–**l**) ascospores in 10% KOH with showing germ slit (arrow); (**m**,**n**) ascospore in distilled water. Scale bars: (**a**,**b**) = 1 cm; (**d**) = 5 mm; (**e**) = 0.5 mm (**f**,**g**) = 20 µm; (**h**–**o**) = 5 µm.

**Figure 11:**
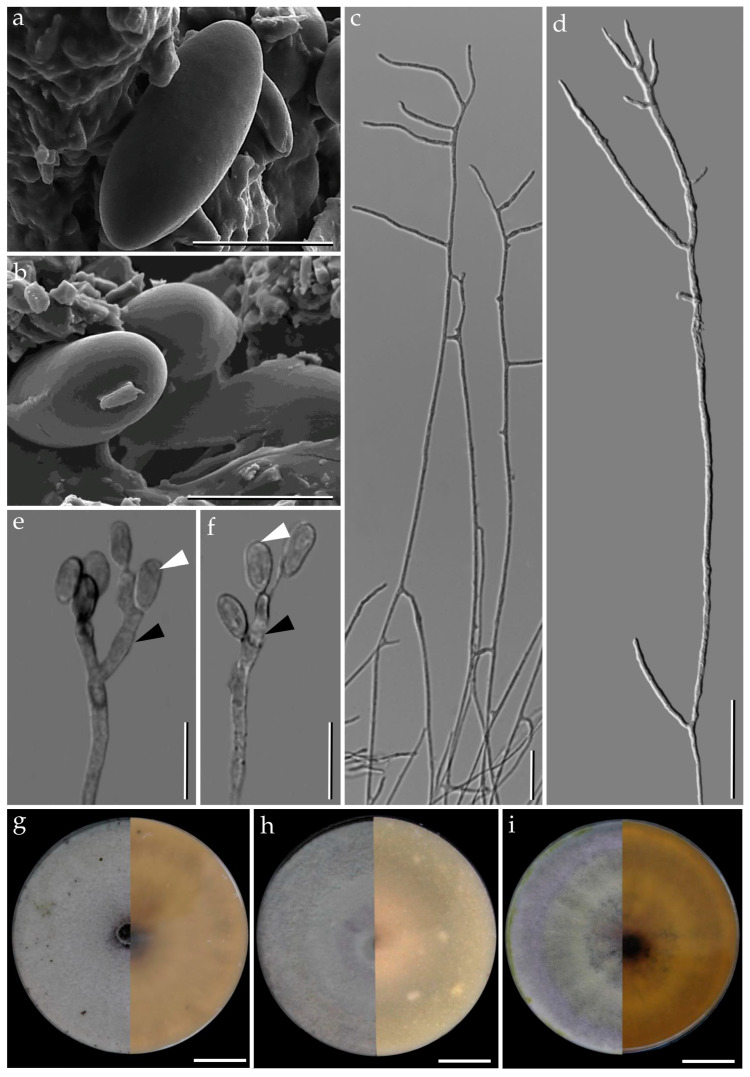
*Pyrenopolyporus papillatus* strain (BCC20324). (**a**,**b**) Ascospores by SEM; (**c**,**d**) aerial mycelium showing branching pattern; (**e**,**f**) conidia (white arrow) and conidiogenous cells (black arrow); (**g**) colony on PDA after one month; (**h**) colony on OA after one month; (**i**) colony on YMGA after on month. Scale bars: (**a**,**b**) = 5 µm; (**c**,**d**) = 20 µm; (**e**,**f**) = 10 µm; (**g**–**i**) = 2 cm.

***Pyrenopolyporus papillatus*** Srikitikulchai, Wongkanoun, M. Stadler and Luangsa-ard, sp. nov.                                                  Figure 10 and Figure 11.

*MycoBank.* MB846449.

*Etymology.* “*papillatus*” based on the papillated ostioles.

*Holotype*. Thailand: Nakhon Si Thammarat Province, Nopphitam, Khao Luang National Park, 8°22′07″ N, 99°44′06″ E, tropical rainforest, on decaying wood, 21 February 2006, P.S., (BBH15197).

*Ex-type culture*. BCC20324; DNA sequences of ex-type culture: (ITS = OP304854), (LSU = OP304874), (*RPB2* = OP981619), (*TUB2* = OQ101846).

*Teleomorph. Stromata* hemispherical to depressed-spherical, widely attached to the substrate, very rarely substipitate, smooth or with inconspicuous perithecial outlines, 25 –33 mm wide, 7–8 mm thick; surface Vinaceous Grey (116), Fuscous Black (104), and Mouse grey (116); blackish brown granules immediately beneath the stromatal surface, with KOH-extractable pigments Purplish Grey (126) or Vinaceous Grey (116); the tissue between perithecia greyish brown to brown, pithy to woody; the tissue below the perithecial layer massive, blackish brown or dark brown, 3.1–3.3 mm thick. *Perithecia* lanceolate, 1.1–1.4 mm long, 0.2–0.3 mm broad. *Ostioles* papillate. *Asci* cylindrical, eight-spored, 168−170 µm in length, the spore-bearing parts, 77−87 µm long, 7−8 µm broad; apical apparatus bluing in Melzer’s reagent, 0.9−1.2 µm long, 2.3−2.7 µm broad. *Ascospores* light brown, ellipsoid, slightly inequilateral, favorably variable, or irregularly shaped, narrowly rounded ends, (11–) 12–13 (–14) × 4–5, ($\overset{-}{x}$= 12.05 × 4.89, *n* = 25 µm); with straight to rarely slightly sigmoid germ slit much less than spore-length or nearly spore-length on the convex side; perispore indehiscent in 10% KOH, epispore smooth.

*Cultures and anamorph*. Colonies on OA covering a 9 cm Petri dish in 1 week, at first whitish becoming velvety to felty, azonate with distinct margins, Pale Green Grey (98); reverse Grey Olivaceous (107). Colonies on YMGA covering a 9 cm Petri dish in 1 week, at first whitish becoming velvety to felty, inconspicuous zonate, Pale Greenish Grey (123); reverse Grey Olivaceous (107). Colonies on PDA, covering a 9 cm Petri dish in 1 week, at first whitish, becoming Pale Greenish Grey (123); reverse Pale Vinaceous (85). *Conidiogenous structures* with virgariella-like branching patterns as defined in Ju and Rogers [2], main axis hyaline to pale brown, finely roughened. *Conidiogenous cells* cylindrical, hyaline, finely roughened, 9−10 × 4−5 µm. *Conidia* hyaline, smooth, ellipsoid, 6−11 × 3−4 µm.

*Additional specimens examined.* Thailand: Nakhon Si Thammarat, Khao Nan National Park, 8°46′14″ N, 99°48′20″ E, tropical rainforest, on decaying wood, 29 October 2008, P.S., (BCC33622, BBH25144).

*Secondary metabolites*. Stromata contain hypoxylone (**1**), BNT (**2**), two isobaric unknown compounds (**4**: [M + Na]^+^ = 258.10997 Da; C_13_H_17_NO_3_), and an unknown hydroxyl derivative of hypoxylone (**3**: [M + H]^+^ = 349.07041 Da; C_20_H_12_O_6_; Figure S8) as major constituents (Figure S7 and Table S2).

*Notes. Pyrenopolyporus papillatus* is morphologically similar to *P. nicaraguensis* by having purple KOH-extractable pigment from the stromatal surface but differs from the latter by having light brown to pale brown ascospores. *Pyrenopolyporus papillatus* differs from *P. nicaraguensis* by having smaller ascospores than *P. nicaraguensis* [(11–) 12–13 (–14) × 4–5 (*P. papillatus*) vs. (11–) 12–15 (–16) × 5.0–6.5 µm (*P. nicaraguensis*)]. Morphologically, *P. hunteri* closely resembles our new fungus but differs by the ascospore size range [11.5–14.0 × 5.0–5.5 (*P. hunteri*) vs. (11–) 12–13 (–14) × 4–5 µm (*P. papillatus*)]. Morphologically, our new species is quite similar to *P. hunteri* and *P. nicaraguensis*, but the molecular phylogeny clearly separates it from the previously reported species. The morphological features and secondary metabolites of *Pyrenopolyporus* species and the allied genus *Daldinia* are summarized in Table S1.

**Figure 12:**
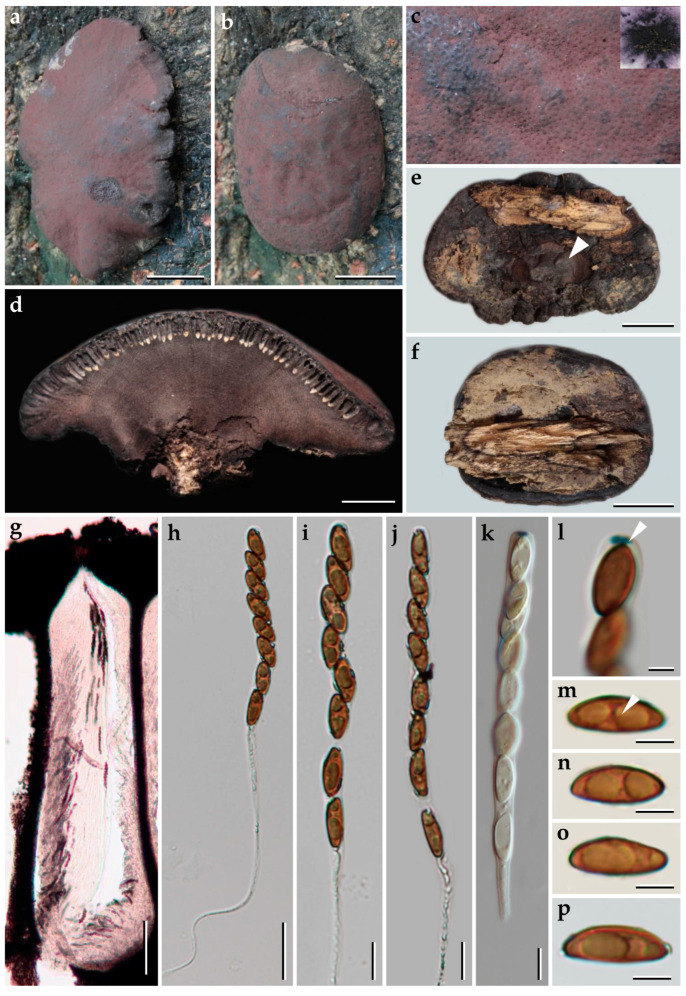
Morphological characteristics of *Pyrenopolyporus tonngachangensis* (BBH25392); (**a**–**d**) stroma on natural habit; (**c**) stromal surface and ostioles with KOH-extractable pigments in 10% KOH; (**d**) longitudinal section of stroma showing perithecia and the tissue below the perithecial layer; (**e**,**f**) detail of stromal insertion point (white arrow); (**g**) longitudinal section perithecia under the light microscope; (**h**–**j**) asci in distilled water; (**k**) young ascus in Melzer’s reagent; (**l**) apical apparatus, bluing in Melzer’s reagent (black arrow); (**m**) ascospore showing germ slit (white arrow); (**n**–**p**) ascospores. Scale bars: (**a**,**b**,**e**,**f**) = 1 cm; (**d**) = 0.5 cm; (**h**) = 20 µm; (**i**–**k**) = 10 µm (**l**–**p**) = 5 µm.

**Figure 13:**
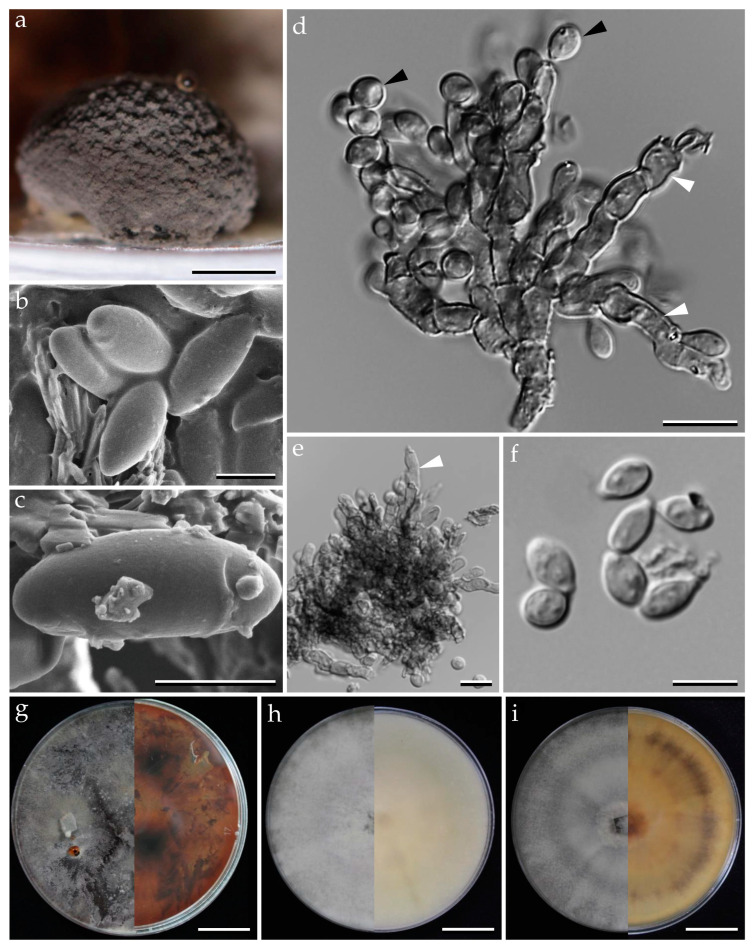
*Pyrenopolyporus tonngachangensis* strain BCC31553. (**a**) Primordia; (**b**,**c**) ascospores by SEM; (**d**,**e**) conidiogenous cells (indicated by white arrows) and conidia (indicated by black arrows); (**g**) colony on PDA after one month; (**h**) colony on OA after one month; (**i**) colony on YMGA after one month. Scale bars: (**a**) = 2 mm; (**b**,**c**) = 5 µm; (**d**–**f**) = 10 µm; (**g**–**i**) = 2 cm.

***Pyrenopolyporus tonngachangensis*** Srikitikulchai, Wongkanoun, Stadler and Luangsa-ard, sp. nov.                                            Figure 12 and Figure 13.

*MycoBank*. MB846674.

*Etymology*. “*tonngachangensis*” referring to the locality “Ton Nga Chang Wildlife Sanctuary” where the type specimen was collected.

*Holotype*. Thailand: Songkhla Province, Hat Yai, Ton Nga Chang Wildlife Sanctuary, 6°57′06” N, 100°13′57” E, tropical rainforest, on decaying wood in the forest, 10 August 2008, P.S., (BBH25392).

*Ex-type culture*. BCC31553; DNA sequences of ex-type culture: (ITS = OP304865), (LSU = OP304887), (*RPB2* = OP981632), (*TUB2* = OQ101847).

*Teleomorph*. *Stromata* solitary or coalescent, hemispherical to depressed-spherical, widely attached to the substrate, very rarely sub-stipitate, smooth or with inconspicuous perithecial outlines, 30–45 mm long, 24–30 mm wide, 5–7 mm thick; surface Purple Slate (102), Fuscous Black (104), Vinaceous Grey (116), and Mouse Grey (118); carbonaceous immediately beneath the stromatal surface, with KOH-extractable pigments Livid Violet (79) or Greyish Lavender (98) and Violet Slate (99); the tissue between perithecia is greyish brown to brown, pithy to woody; the tissues below the perithecial layer are greyish brown, soft-textured, with a lamellate structure consisting of densely intricate small black and golden-brown lines, 5.0–5.7 mm thick. *Perithecia* lanceolate, 1.1–1.2 mm high, 0.2–0.3 mm broad. *Ostioles* conspicuous, umbilicate. *Asci* cylindrical, eight-spored, 170.0−207.5 µm in length, the spore-bearing parts, 75−83 µm long, 7 µm broad; apical apparatus bluing in Melzer’s reagent, 1.5–1.7 µm long, 3.4–3.8 µm broad. *Ascospores* light brown, ellipsoid, slightly inequilateral or irregularly shaped, narrowly rounded ends, (12–) 13–14 (–16) × 4–5 ($\overset{-}{x}$ = 13.48 × 4.91 µm, *n* = 25); with straight germ slit covering ca. 2/3 length on the convex side; perispore indehiscent in 10% KOH, epispore smooth.

*Cultures and anamorph*. Colonies on OA covering a 9 cm Petri dish in 1 week, inconspicuous zonate with distinct margins, at first whitish becoming Pale Greenish Grey (123) and Pale Olivaceous Grey (120); reverse Pale Olivaceous Grey (120). Immature stromata hemispherical, 5.7 × 5 mm. Colonies on YMGA covering a 9 cm Petri dish in 1 week, zonate, at first aerial mycelium whitish becoming velvety to felty, Pale Greenish (123); reverse Green Olivaceous (107) and Smoke Grey (105). Colonies on PDA covering a 9 cm Petri dish in 1 week, inconspicuous zonate, at first aerial mycelium whitish becoming Sepia (63), Dark Vinaceous (82), and Dark Brick (66); reverse Brown Vinaceous (84). *Primordia* hemispherical, 5.7 × 5.0 mm. *Conidiophores* loosely arranged, branched, undetermined in length, 2–3 μm broad. *Conidiogenous cells* produced holoblastically, cymbiform, rarely subglobose to obovoid, hyaline, 9–10 × 4–5 μm, each cell producing one or several conidia. *Conidia* hyaline, smooth, subglobose, obovoid, ellipsoid with flattened base, (4–) 5–6 (–7) × (3–) 4–5 µm. ($\overset{-}{x}$ = 5.44 × 4.11 µm, *n* = 25).

*Additional specimens examined*. Thailand: Songkhla Province, Hat Yai, Ton Nga Chang Wildlife Sanctuary, 6°57′06″ N, 100°13′57″ E, tropical rainforest forest, on decaying wood, 10 August 2008, P.S., (BCC31555). Chiang Mai Province, Ban Saluang Nok Community Forest, 19°01′06″ N, 98°53′26″ E, hill evergreen forest, on decaying wood, 8 October 2019, P.S., (BCC91227).

*Secondary metabolites*. Stromata contain hypoxylone (**1**), BNT (**2**), and an unknown hydroxyl derivative of hypoxylone (**3**: [M + H]^+^ = 349.07041 Da; C_20_H_12_O_6_; Figure S8) as major constituents (Figure S7 and Table S2).

*Notes*. The morphological features of *Pyrenopolyporus tonngachangensis* closely resembles *P. hunteri* and *P. papillatus* with the light brown color of ascospores and produces dark livid purple KOH-extractable pigment on the stromatal surface. However, the morphological features of *P. tongngachangensis* differ from *P. hunteri* by having conspicuous umbilicate ostioles. The ascospores of *P. tonngachangensis* are also larger than *P. hunteri* [(12–)13–14(–16) × 4–5 for *P. tonngachangensis* vs. 11.5–14 × 5–5.5 µm for *P. hunteri*]. *Pyrenopolyporus papillatus* differs from *P. tonngachangenis* by showing conspicuous papillate ostioles. Our molecular phylogeny also confirmed the above phenotypic data.

### 3.2. Molecular Phylogeny (Figure 14)

**Figure 14:**
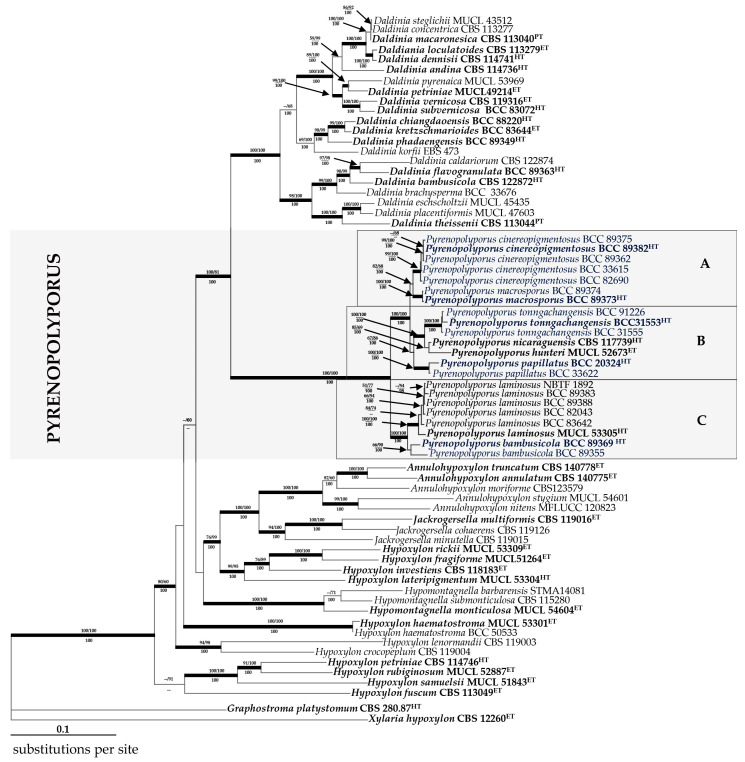
Phylogeny of the Hypoxylaceae. RAxML tree was generated based on multiple loci alignment of concatenated ribosomal (ITS and LSU) and proteinogenic (*TUB2* and *RPB2*) sequence data. Support values were calculated via MP, ML, and Bayesian analyses and are indicated above (MPBS/MLBS) and below (BPP) the respective branches. Branches of significant support (BS ≥ 70% and PP ≥ 100) are thickened. New species are indicated in blue and the clade comprising the sequences of the *Pyrenopolyporus* spp. is marked by a grey rectangle consisting of subclades A, B and C; ET indicates ex-epitype, HT ex-holotype, and PT ex-paratype strains are highlighted in **bold** letters.

After providing the full taxonomic description of the five novel species and a new record of *Pyrenopolyporus* sp. for Thailand, we have also confirmed their taxonomic position through multi-locus phylogenetic analyses as shown in Figure 14 and single-gene analyses as shown in Figures S1–S5. The 70 newly generated ITS, LSU, *RPB2*, and *TUB2* sequences were compared with data from the GenBank NCBI nucleotide database. This was performed to clarify the phylogenetic placement of newly collected Thai specimens of Hypoxylaceae and to distinguish them from other species and genera in the Xylariales (PCR amplifications yielded approximately 500 bp, 1000 bp, 800 bp, and 1000 bp of ITS rDNA, LSU rDNA, *RPB2*, *TUB2* sequences, respectively). The phylogenetic relationships were estimated using the MP, ML, and MB analyses. The dataset of the multi-locus DNA sequences included 66 taxa from the Hypoxylaceae: *Annulohypoxylon* (5), *Daldinia* (21), *Hypoxylon* (12), *Hypomontagnella* (3), *Jackrogersella* (3), and *Pyrenopolyporus* (22). The combined dataset consisted of 5131 characters, of which 2954 were constant, 1722 were parsimony informative, and 455 were uninformative. The MP analysis yielded 11650 trees with a CI of 0.333, a RI of 0.648, and a HI of 0.667. The best phylogenetic tree inferred from RAxML had a likelihood of −56675.811. The alignment had 2456 distinct alignment patterns, with 23.49% undetermined characters or gaps. Estimated base frequencies were as follows: A = 0.239, C = 0.264, G = 0.261, T = 0.234; substitution rates were AC = 1.713, AG = 4.654, AT = 1.599, CG = 1.104, CT = 7.949, GT = 1.000; gamma distribution shape parameter was α 0.841. The likelihood of the Bayesian tree was −65650.640. As shown in Figure 14, the sequences of the new Thai strains are well separated from the previously proposed *Pyrenopolyporus* species, while the Thai specimens of *P. laminosus* clustered with the holotype that was originally reported from the Caribbean by Kuhnert et al. [28]. As the topology of the RAxML tree is practically identical to the one presented by Wendt et al. [3], from which most DNA sequence data were included in our study and analyzed using essentially the same methodology, we restrict our discussion on the phylogenetic positions of the new taxa.

### 3.3. MALDI-TOF Mass Spectrometry (Figure 15, Figure 16 and Figure 17)

**Figure 15:**
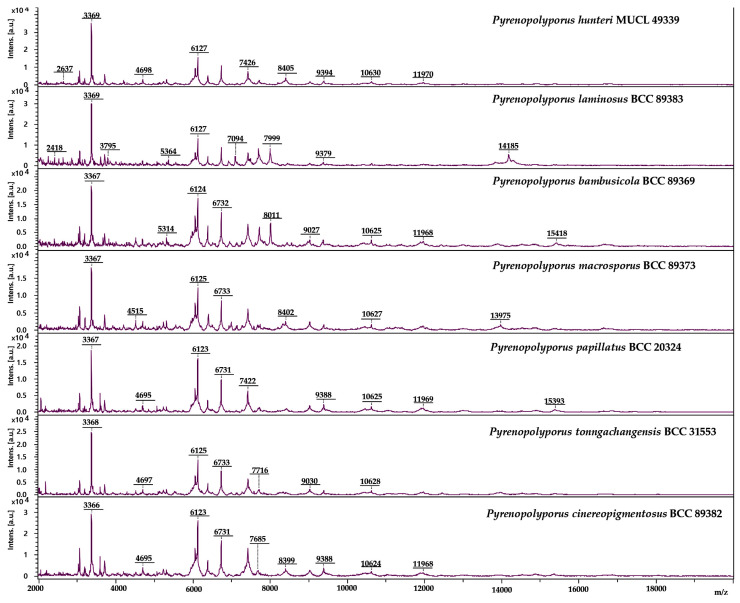
Schematic representation of MALDI-TOF chromatograms from mycelial peptide extracts of *Pyrenopolyporus* spp. evaluated in this study. All spectra shown are baseline-subtracted, smoothed and with the y-axis auto-scaled covering the mass range from 2 kDa to 20 kDa (with x-axis scale increments of 2 kDa).

We also investigated the peptide mass fingerprint (PMF) via MALDI-TOF MS of our samples after providing full taxonomic characterization to support our hypothesis regarding the discrimination of closely related species. Representative samples of each species from *Pyrenopolyporus*, including the ex-epitype culture of the type species of the genus (*P. hunteri*; Figure 15), were analyzed using MALDI-TOF MS; three *Daldinia* spp. isolates were also included for this comparison. All samples delivered high quality MALDI spectra (peak rich) as shown in Figure S6. The principal component analysis (PCA) of the 82 final peaks (after denoising, recalibration, and negative-control subtraction) showed a clear difference between the genera *Pyrenopolyporus* and *Daldinia*; statistical analyses based on 2D peak distribution using the software ClinProTools gave results in agreement with the PCA mentioned above by revealing two proteomic markers that can be used for discriminating *Pyrenopolyporus* spp. from *Daldinia* spp. at 6734 and 3592 Da (Figure 17a).

**Figure 16:**
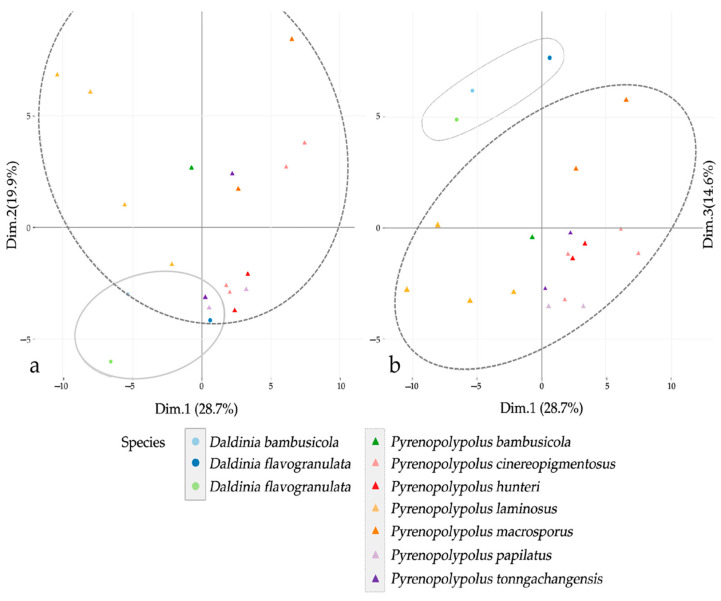
Principal component analysis (PCA) of denoised, recalibrated, and negative-control subtracted 82 MALDI-TOF mass spectra; (**a**) the coordinate plane between the first (Dim.1) and second (Dim.2) components; (**b**) the coordinate plane between the first (Dim.1) and the third components (Dim.3). The ovals on the figures represent 95% confidence concentration ellipses.

**Figure 17:**
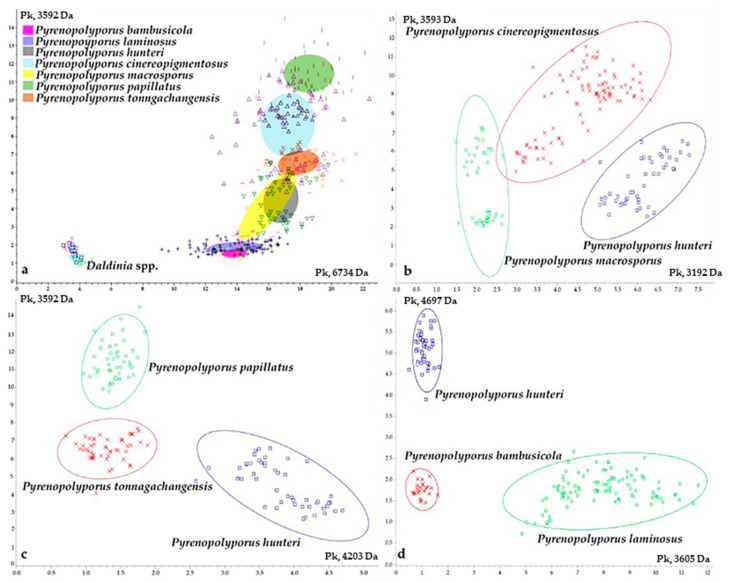
Principal component analysis (PCA) of the two most discriminant mass spectra peaks derived from the fungal isolates included in this study; (**a**) PCA for all *Pyrenopolyporus* and *Daldinia* strains. *P. bambusicola* (pink), *P. laminosus* (purple), *P. hunteri* (grey), *P. cinereopigmentosus* (greyish blue), *P. macrosporus* (yellow), *P. papillatus* (green), *P. tonngachangensis* (orange); (**b**) PCA including only *P. cinereopigmentosus* (red), *P. hunteri* (blue), and *P. macrosporus* (green); (**c**) PCA including only *P. hunteri* (blue), *P. papillatus* (green), and *P. tonngachangensis* (red); (**d**) PCA including only *P. bambusicola* (red), *P. hunteri* (blue), and *P. laminosus* (green).The ovals on the figures represent 95%-confidence concentration ellipses.

Despite an overall similarity between *Pyrenopolyporus* species as revealed by the PCA, ClinProTools was able to give the two most discriminating molecules between some pairs of species (Figure 17b–d). The species within *Pyrenopolyporus* appeared more or less overlapped except for *P. laminosus* and *P. macrosporus* (Figure 16). By dividing the samples into three groups following taxonomic position of our gene multi loci analyses, group **A** comprised *P. cinereopigmentosus* and *P. macrosporus*; group **B** comprised *P. hunteri*, *P. papillatus*, and *P. tonngachangensis*; group **C** comprised *P. bambusicola* and *P. laminosus*. There is an increased resolution in the discrimination of the species.

Group **A** (*Pyrenopolyporus cinereopigmentosus* and *P. macrosporus*) shared common peaks at 6734 Da while they can be discriminated from each other by the averaged peak area/intensity distribution pattern of the molecules at 3192 and 3594 Da (Figure 17b). The molecular phylogenetic placement was also confirmed in that *P. cinereopigmentosus* was clearly distinct from *P. macrosporus*, with full supports for all phylogenetic inferences (MP, ML, MB).

Group **B** (*Pyrenopolyporus hunteri*, *P. papillatus*, and *P. tonngachangensis*) shared common peaks at 6734 Da; they can be clearly discriminated from each other by the averaged peak area/intensity distribution pattern of the molecules at 3592 and 4203 Da (Figure 17c). Our molecular phylogeny also confirmed that *P. papillatus* and *P. tonngachangensis* were clearly distinct from *P. hunteri* with full support for all phylogenetic inferences (MP, ML, MB).

**Figure 18:**
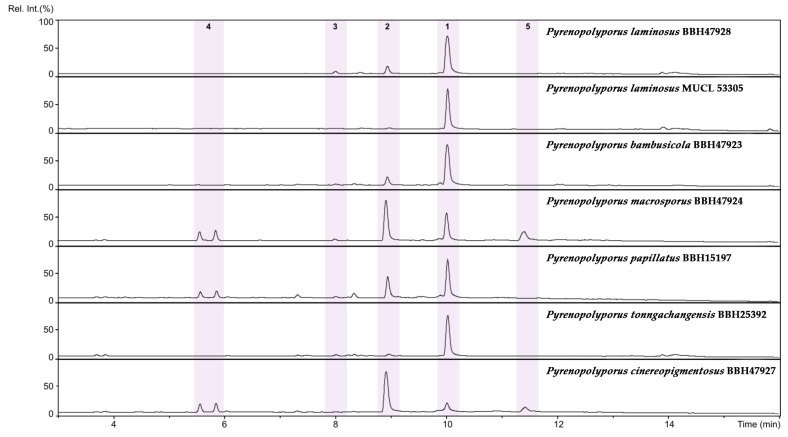
HPLC–UV/vis chromatograms (210 nm) of stromatal extracts of the evaluated *Pyrenopolyporus* spp. in this study. **1**: Hypoxylone [M + H]^+^: 333.07588 Da; C_20_H_12_O_5_; **2**: BNT [M + H]^+^: 319.09643 Da; C_20_H_14_O_4_; **3**: Unknown hypoxylone derivative [M + H]^+^: 349.07041 Da; C_20_H_12_O_6_; **4**: Unknown isobaric metabolites [M + H]^+^: 258.10997 Da; C_13_H_17_NO_3_; **5**: Unknown compound [M + H]^+^: 633.45371 Da.

Group **C** was phylogenetically segregated from other species with high statistical support (Clade C, Figure 14). The two species within this group (*Pyrenopolyporus bambusicola* and *P. laminosus*) are very challenging for identification using morphological features. The proteomic profiling also showed the similarity between these two species; they seemed to share common peaks at 6734 and 3592 Da while being discriminated by the two molecules at 3605 and 4697 Da (Figure 17d). In general, this resolution was much better than the chemotaxonomic study by HPLC-DAD/MS (Figure 18).

**Dichotomous key to the species of *Pyrenopolyporus***
**1a.** Ascospores highly variable in shape, ellipsoid to slightly ellipsoid-inequilateral……...**2**
**1b.** Ascospores less variable in shape, ellipsoid-inequilateral ….............................................**4**
**2a.** KOH-extractable pigments without purple shades; ascospores 9.5–14(–16) × 4–6 µm with straight to slightly sigmoid germ slit; much less than spore length on the more convex side ....................................................................................................................……***P. tortisporus***
**2b**. KOH-extractable pigments purple, ascospores with straight germ slit less than spore length………………………………...……………………………………………………………**3**
**3a**. Ascospores 9.5–12 (−13) × 4–5 µm with straight germ slit less than spore length frequently on the more flattened side..........................................................................***P. symphyon***
**3b.** Ascospores (14–) 16–17 × (6–) 7–8 µm with straight germ slit covering full spore length on convex side…………………………………………………………………... ***P. macrosporus***
**4a.** Ascospores pale brown to light brown……………..………………………....…………....**5**
**4b.** Ascospores brown to dark brown…………………………………………..……...……….**7**
**5a.** Ostioles umbilicate; ascospores (12–) 13–14 (–16) × 4–5 µm with straight to rarely slightly sigmoid germ slit much less than spore-length or nearly spore-length on the convex side…...…………..…………………..……………...………..….….…***P. tonngachangensis***
**5b.** Ostioles lower than stromatal surface, punctiform, papillate ……………...…………….**6**
**6a.** Ostioles punctiform, slightly lower than the stromal surface, ascospores 11.5–14 × 5–5.5 µm with straight germ slit much less than spore-length……………..……………..***P. hunteri***
**6b.** Ostioles papillate; ascospores, (11–) 12–13 (–14) × 4–5 µm with straight germ slit much less than spore-length ……..……………………...………………………...…….. ***P. papillatus***
**7a.** Species occurring on bamboos………………………….………...….……………..……….**8**
**7b.** Species on woody, dicot substrates…………………………………………………………**9**
**8a.** Stromata found in fire-damaged areas; ostioles conspicuous umbilicate; perithecia long tubular, 0.75–0.9 mm high; ascospores 10–11 (–12) × (3–) 4–5 µm ………..…***P. bambusicola***
**8b.** Stromata not found in fire-damaged area; ostioles umbilicate black to inconspicuous; perithecia long tubular 0.75–0.90 mm high, ascospores 11.0–13.5 × 4.2–4.5 µm...............................................................................................................................***P. laminosus***
**9a.** KOH-extractable pigment Dark Livid and Livid Purple; perithecia 0.8–1.5 mm high, ascospores (11.5–) 12–15(−16) × 5–6.5 μm…………………….……..………..***P. nicaraguensis***
**9b.** KOH-extractable pigment Dark Mouse Grey or Iron Grey; perithecia tubular 0.9–1.1 mm high ascospores (12–) 13–14 (–15) × 6–7 μm ……………………...***P. cinereopigmentosus***

## 4. Discussion

Most *Pyrenopolyporus* species are morphologically highly similar, which makes species delimitation and identification based on morphology alone difficult and confusing [2]. Recently, much progress has been achieved thanks to DNA sequence data, particularly of protein-coding genes such as *RPB2* or *TUB2*, which have superior resolution compared to ITS or LSU [3,8]. However, an obstacle for an improved species delimitation and identification is the lack of sequences for type materials or well-identified reference specimens in GenBank. *Pyrenopolyporus hunteri*, the type species of the genus is a good example of these problems. Its taxonomy has been re-investigated by Wendt et al. [3]. In this study, we examined the phylogenetic relationships of our fresh collections with the species of *Pyrenopolyporus* spp. for which multi-gene sequence data are available. We have performed a multi-gene analysis using ITS, LSU, *RPB2*, and *TUB2* sequence data to determine the phylogenetic placement of our specimens. *Pyrenopolyporus* clearly forms a monophyletic clade within Hypoxylaceae, distinct from the genus *Daldinia*, which is in accordance with the extensive results of Wendt et al. [3]. Considering our molecular phylogenetic analyses, the clade *Pyrenopolyporus* is split into three strongly supported subclades and formed a sister group to the genus *Daldinia*.

Subclade **A** is comprised of *Pyrenopolyporus cinereopingmentosus* and *P. macrosporus*, which share similar morphological features such as having darker ascospore color. Considering the molecular phylogeny, the two new species including are closely related, but strongly segregated into two distinct monophyletic clades with high supports. The morphological comparisons between these two new species demonstrates very similar features with dark brown ascospores and purple stromal KOH-extractable pigment, as well as similar stromatal secondary metabolites. However, *P. macrosporus* has the largest ascospores with very diverse forms compared to *P. cinereopigmentosus*. Hence, our combination between morphological characterization and multi-locus phylogeny supports the status of distinct species between them. However, the proteomics and metabolomics data could not allow a clear distinction to closely differentiate between both species. Morphologically, *P. macrosporus* is also similar to *P. tortisporus* and *P. symphyon* but differs by its ascospore morphology and stromatal KOH-extractable pigments that we have already mentioned in the notes accompanying the species description. The pantropical species, *P. tortisporus*, was first reported by Ju and Rogers [2]. The type specimen of this species originated from NY as specimen no. WSP69643. The phenotypic features of this fungus are clearly distinctive from *P. macrosporus* and other species by having frequently deformed ascospores and producing an olivaceous pigment in 10% KOH solution. Fournier et al. [44] also provided a new illustration of a specimen discovered in the French West Indies, whose morphological features fitted well with Ju and Rogers’ description. *Pyrenopolyporus symphyon* was first reported by Möller [43] but has no appropriate specimen for reexamination since Ju and Rogers [2] reported the type specimen to be immature. The fungus thus needs to be collected in a fresh state for epitypification of the species.

Subclade **B** is comprised of the type species *Pyrenopolyporus hunteri* along with its sister species *P. nicaraguensis* and other closely related species including *P. tonngachangensis* and *P. papillatus. Pyrenopolyporus hunteri* and *P. nicaraguensis* closely resemble *P. papillatus* and *P. tonngachangensis* regarding the appearance of the morphological characterization (see in the notes of taxonomic description). Kuhnert et al. [28] found hypoxylone (a naphthoquinone) from fresh specimens of *P. laminosus*, similar to the finding in *P. hunteri* and *P*. *nicaraguensis* Bitzer et al. [6]. This naphthoquinone could represent an additional chemotaxonomic marker for the species group comprising *P. laminosus* and its closely related species. *Pyrenopolyporus hunteri* and *P. nicaraguensis* were listed (under the epithets *H. polyporum* and *H. nicaraguense*) by Ju and Rogers [2] as members of the genus *Hypoxylon*, and regarded as closely related to *Hypoxylon sclerophaeum* [45]. These species were considered as part of the “*H. placentiforme* line” circumscribed by Ju and Rogers [2], characterized by massive semiglobose to peltate stromata with a solid lamellate interior at times with radiating black strands, in contrast to the interior zonate characteristic of the genus *Daldinia*. Despite morphological differences, *H. placentiforme* has been transferred to *Daldinia* (as *D. placentiformis*) based on the phylogenetic analyses by Hsieh et al. [25], corroborated by chemotaxonomic evidence [5].

The molecular phylogenetic analyses also showed that the species within the subclade **B** (*P. hunteri*, *P. nicaraguensis*, *P. papillatus*, and *P. tonngachangensis*) were clearly segregated from the other species. We did not have any axenic cultures from *P*. *nicaraguensis* to test whether its proteomic profile would be different from the other species of this subclade; whereas, the MALDI-TOF/MS data allowed a distinction between *P. hunteri*, *P. papillatus* and *P. tonngachangensis*, consistent with the molecular phylogenetic results. Therefore, our study, through the MALDI-TOF/MS data, does not only confirm the distinction between *Daldinia* and *Pyrenopolyporus*, but also the differences between the species within the subclade **B** in those for whom cultures are available. In contrast, the stromatal metabolite profile for *P. papillatus* and *P. tonngachangensis* showed high similarity to the profiles obtained for species in the subclade C.

Subclade **C** consists exclusively of bambusicolous species, *Pyrenopolyporus bambusicola* and *P. laminosus*. *Pyrenopolyporus laminosus* is well discriminated by daldinioid stromata with violet KOH-extractable pigments and light brown ascospores with a spore-lengthed germ slit and indehiscent perispore in 10% KOH, and by the occurrence on bamboos. Amongst *Pyrenopolyporus* spp. having peltate stromata with violet KOH-extractable pigments, *P. nicaraguensis* is the most similar to *P. laminosus* as it has ascospores with a germ slit covering almost the entire spore length. The lamellate structure of the interior tissue of the stroma of *P. nicaraguensis* has a similar appearance to that of *P. laminosus*. However, *P. nicaraguensis* differs in having raised discoid ostioles and broader ellipsoid ascospore and it has been almost exclusively reported to occur on dicotyledons [2,28]. Our novel species, *P. bambusicola,* was found only on bamboos and is distinguished from other members of this genus by deeply umbilicate ostioles and the ascospore size range. The phylogenetic analyses clearly support its distinctiveness. Furthermore, the MALDI-TOF/MS seems to support its difference from the other *Pyrenopolyporus* species. In particular, *P. laminosus* can be discriminated from its sister species *P. bambusicola*. These two sister species are highly similar for their morphology.

MALDI-TOF/MS has been demonstrated to be a highly adaptable approach for efficient identification and classification of bacteria and yeasts in clinical laboratories, presenting a complementary technique to traditional microscopic and molecular biology methods [11,12]. However, this technique has not been used extensively for the identification or classification of filamentous fungi for various reasons, including the difficulty of fungal protein extraction and the necessary high capital investment. In this study, the technique served well to discriminate a complicated species complex and provided corroborating evidence to other data that were obtained by studying classical morphology, chemotaxonomy, and molecular phylogeny. The MALDI-TOF/MS analysis allowed a good discrimination between the different *Pyrenopolyporus* species, and particularly high resolution between *Daldinia* and *Pyrenopolyporus*, coherently with our phylogenetic analysis. Although the sampling might be limited in our study, the data encourage further investigations with more samples of the same genus, or even other species complexes in the Hypoxylaceae in order to further prove the robustness of the technique.

Moreover, our study advocates that the PMF obtained via MALDI-TOF/MS can be used as a reliable tool for species discrimination in *Pyrenopolyporus*. Despite the highly similar morphological traits of the fungi in this group, the PMF data demonstrated that *Pyrenopolyporus* species are distinguishable, consistent with molecular phylogenetic data. Cryptic morphology in fungal species complexes has long been a problem for taxonomists. Our study showed that molecular phylogenies based on multi-locus analyses and PMF could contribute to resolve species identification.

Recently, some strains representing important lineages of the Hypoxylaceae have been selected for a phylogenomic study relying on high quality genomes [9], revealing the occurrence of ITS polymorphisms [46] and thus the necessity to use more than ITS for species identification and classification in this family and the order Xylariales in general. This accomplishment has offered a significant starting point for the development of a stable phylogeny of this order, as well as studies on evolution, ecological guilds, and natural product biosynthesis.

## Figures and Tables

**Table 1:** List of all taxa used in the current phylogenetic study.

Taxa	Strain/Status	Origin	GenBank Accession Number				Reference
			ITS	LSU	*RPB2*	*TUB2*	
*Annulohypoxylon annulatum*	CBS 140775/ET	Texas	KY610418	KY610418	KY624263	KX376353	ITS, LSU, *RPB2*: [3]; TUB2: [23]
*A. moriforme*	CBS 123579	Martinique	KX376321	KY610425	KY624289	KX271261	ITS, *TUB2*: [23]; *RPB2*, LSU: [3]
*A. nitens*	MFLUCC 12.0823	Thailand	KJ934991	KJ934992	KJ934994	KJ934993	[24]
*A. stygium*	MUCL 54601	French Guiana	KY610409	KY610475	KY624292	KX271263	[3]
*A. truncatum*	CBS 140778/ET	Texas	KY610419	KY610419	KY624277	KX376352	*TUB2*: [23]; ITS, LSU, *RBP2*: [3]
*Daldinia andina*	CBS 114736/HT	Ecuador	AM749918	KY610430	KY624239	KC977259	ITS: [5]; *TUB2*: [23]; LSU, *RPB2*: [3]
*D. bambusicola*	CBS 122872/HT	Thailand	KY610385	KY610431	KY624241	AY951688	*TUB2*: [25]; ITS, LSU, *RPB2*: [3]
*D. brachysperma*	BCC33676	Thailand	MN153854	MN153871	N/a	MN172205	[26]
*D. caldariorum*	CBS122874	USA	KU683756	KU683756	KU684289	KU684128	[27]
*D. chiangdaoensis*	BCC88220/HT	Thailand	MN153850	MN153867	MN172208	MN172197	[26]
*D. concentrica*	CBS 113277	Germany	AY616683	KY610434	KY624243	KC977274	ITS: [7]; *TUB2*: [28]; LSU, *RPB2*: [3]
*D. dennisii*	CBS 114741/HT	Australia	JX658477	KY610435	KY624244	KC977262	ITS: [29]; *TUB2*: [28]; LSU, *RPB2*: [3]
*D. eschscholtzii*	MUCL 45435	Benin	JX658484	KY610437	KY624246	KC977266	ITS: [29]; *TUB2*: [28]; LSU, *RPB2*: [3]
*D. flavogranulata*	BCC89363/HT	Thailand	MN153856	MN153873	MN172211	MN172200	[26]
*D. korfii*	EBS 067	Argentina	KY204018	N/A	N/A	KY204014	[5]
*D. kretzschmarioides*	TBRC 8875/ET	Thailand	MH938531	MH938540	MK165425	MK165416	[30]
*D. loculatoides*	CBS 113279/ET	UK	AF176982	KY610438	KY624247	KX271246	ITS: [31]; LSU, *RPB2*, *TUB2*: [3]
*D. macaronesica*	CBS 113040/PT	Spain	KY610398	KY610477	KY624294	KX271266	[3]
*D. padaengensis*	BCC89349/HT	Thailand	MN153852	MN153869	MN172206	MN172195	[26]
*D. petriniae*	MUCL 49214/ET	Austria	AM749937	KY610439	KY624248	KC977261	ITS: [5]; *TUB2*: [28]; LSU, *RPB2*: [3]
*D. placentiformis*	MUCL 47603	Mexico	AM749921	KY610440	KY624249	KC977278	ITS: [5]; *TUB2*: [28]; LSU, *RPB2*: [3]
*D. pyrenaica*	MUCL 53969	France	KY610413	KY610413	KY624274	KY624312	[3]
*D. steglichii*	MUCL 43512	Papua New Guinea	KY610399	KY610479	KY624250	KX271269	[3]
*D. subvernicosa*	TBRC 8877/HT	Thailand	MH938533	MH938542	MK165430	MK165421	[30]
*D. theissenii*	CBS 113044/PT	Argentina	KY610388	KY610441	KY624251	KX271247	[3]
*D. vernicosa*	CBS 119316/ET	Germany	KY610395	KY610442	KY624252	KC977260	*TUB2*: [28]; ITS, LSU, *RPB2*: [3]
*Graphostroma platystomum*	CBS 270.87/ET	France	JX658535	DQ836906	KY624296	HG934108	ITS: [29]; LSU: [32]; *TUB2*: [33], *RPB2*: [3]
*Hypomontagnella barbarensis*	STMA 14081/HT	Argentina	MK131720	MK131718	MK135891	MK135893	[34]
*Hy. monticulosa*	MUCL 54604/ET	French Guiana	KY610404	KY610487	KY624305	KX271273	[34]
*Hy. submonticulosa*	CBS 115280	France	KC968923	KY610457	KY624226	KC977267	ITS, *TUB2*: [28]; LSU, *RPB2*: [3]
*Hypoxylon crocopeplum*	CBS 119004	France	KC968907	KY610445	KY624255	KC977268	ITS, *TUB2*: [28]; LSU, *RPB2*: [3]
*H. fragiforme*	MUCL 51264/ET	Germany	KC477229	KM186295	KM186296	KX271282	ITS: [35]; LSU, *RPB2*: [13]; *TUB2*: [3]
*H. fuscum*	CBS 113049/ET	France	KY610401	KY610482	KY624299	KX271271	[3]
*H. haematostroma*	MUCL 53301/ET	Martinique	KC968911	KY610484	KY624301	KC977291	ITS, *TUB2*: [28]; LSU, *RPB2*: [3]
*H. haematostroma*	BCC50533	Thailand	MN153866	MN153883	MN172221	MN172204	[30]
*H. investiens*	CBS 118183/ET	Malaysia	KC968925	KY610450	KY624259	KC977270	ITS, *TUB2*: [28]; LSU, *RPB2*: [3]
*H. lateripigmentum*	MUCL 53304/HT	Martinique	KC968933	KY610486	KY624304	KC977290	ITS, *TUB2*: [28]; LSU, *RPB2*: [3]
*H. lenormandii*	CBS 119003	Ecuador	KC968943	KY610452	KY624261	KC977273	ITS, *TUB2*: [28]; LSU, *RPB2*: [3]
*H. petriniae*	CBS 114746/HT	France	KY610405	KY610491	KY624279	KX271274	*TUB2*: [28]; ITS, LSU, RPB2, TUB2: [3]
*H. rickii*	MUCL 53309/ET	Martinique	KC968932	KY610416	KY624281	KC977288	ITS, *TUB2*: [28]; LSU, *RPB2*: [3]
*H.rubiginosum*	MUCL 52887/ET	Germany	KC477232	KY610469	KY624266	KY624311	ITS: [35]; LSU, *RPB2*, *TUB2*: [3]
*H. samuelsii*	MUCL 51843/ET	Guadeloupe	KC968916	KY610466	KY624269	KC977286	ITS, *TUB2*: [28]; LSU, *RPB2*: [3]
*J. cohaerens*	CBS 119126	Germany	KY610396	KY610497	KY624270	KY624314	[3]
*J. minutella*	CBS 119015	Portugal	KY610381	KY610424	KY624235	KX271240	TUB2: [28]; ITS, LSU, RPB2: [3]
*J. multiformis*	CBS 119016/ET	Germany	KC477234	KY610473	KY624290	KX271262	ITS: [28]; *TUB2*: [23]; LSU, *RPB2*: [3]
***Pyrenopolyporus bambusicola***	**BCC89355/HT**	**Thailand**	**OP304856**	**OP304876**	**OP981624**	**OQ101839**	**This study**
***P. bambusicola***	**BCC89369**	**Thailand**	**OP304858**	**OP304878**	**OP981623**	**OQ101840**	**This study**
***P. cinereopigmentosus***	**BCC89362**	**Thailand**	**OP304857**	**OP304877**	**OP981625**	**OQ101841**	**This study**
***P. cinereopigmentosus***	**BCC89375**	**Thailand**	**OP304859**	**OP304881**	**OP981626**	**OQ101842**	**This study**
***P. cinereopigmentosus***	**BCC89382/HT**	**Thailand**	**OP304860**	**OP304882**	**OP981627**	**OQ101843**	**This study**
***P. cinereopigmentosus***	**BCC33615**	**Thailand**	**OP304867**	**OP304889**	**OP981628**	**OQ101839**	**This study**
***P. cinereopigmentosus***	**BCC82690**	**Thailand**	**OP304868**	**OP304890**	**OP981629**	**OQ101840**	**This study**
*P. hunteri*	MUCL 52673/ET	Ivory Coast	KY610421	KY610472	KY624309	KU159530	*TUB2*: [20]; ITS, LSU, *RPB2*: [3]
*P. laminosus*	MUCL 53305	Martinique	KC968934	KY610485	KY624303	KC977292	ITS, *TUB2*: [28]; LSU, *RPB2*: [3]
*P. laminosus*	NBTF1892	Thailand	OP304864	OQ123731	N/A	OQ032514	This study
*P. laminosus*	BCC89383	Thailand	MN153855	MN153872	MN172210	MN172199	[26]
*P. laminosus*	BCC89388	Thailand	OP304861	OP304883	OP981634	OQ032513	This study
*P. laminosus*	BCC82043	Thailand	OP304855	OP304875	OP981633	OQ032515	This study
*P. laminosus*	BCC83642	Thailand	OP304863	OP304885	OP981635	OQ032516	This study
***P. macrosporus***	**BCC89373/HT**	**Thailand**	**OP304870**	**OP304879**	**OP981621**	**OQ101844**	**This study**
***P. macrosporus***	**BCC89374**	**Thailand**	**OP304871**	**OP304880**	**OP981622**	**OQ101845**	**This study**
*P. nicaraguensis*	CBS 117739/HT	Burkina Faso	AM749922	KY610489	KY624307	KC977272	ITS: [5]; *TUB*: [28]; LSU, *RPB2*: [3]
***P. papillatus***	**BCC20324/HT**	**Thailand**	**OP304854**	**OP304874**	**OP981619**	**OQ101846**	**This study**
***P. papillatus***	**BCC33622**	**Thailand**	**OP304869**	**OP304891**	**OP981620**	**N/A**	**This study**
***P. tonngachangensis***	**BCC31553/HT**	**Thailand**	**OP304865**	**OP304887**	**OP981632**	**OQ101847**	**This study**
***P. tonngachangensis***	**BCC31555**	**Thailand**	**OP304866**	**OP304888**	**OP981630**	**OQ101848**	**This study**
***P. tonngachangensis***	**BCC91226**	**Thailand**	**OP304862**	**OP304884**	**OP981631**	**OQ101849**	**This study**
*Xylaria hypoxylon*	CBS12260/HT	Sweden	KY610407	KY610495	KY624231	KX271279	*TUB2*: [36]; ITS, LSU, *RPB2*: [3]

New taxa proposed in this study are in bold. ET indicates epitype, HT holotype, and PT paratype. N/A, Data not available. Acronyms of culture collections: BCC, BIOTEC Culture Collection, Pathum Thani, Thailand; CBS, Centraalbureau voor Schimmelcultures, CBS-KNAW Culture, Utrecht, Netherlands; EBS, Fundación Miguel Lillo, San Miguel de Tucumán, Argentina; MFLUCC, Mae Fah Luang culture collection; MUCL, Laboratory of Mycology, which is part of the Earth and Life Institute (ELI), in particular the Pole of Applied Microbiology (ELIM) of the Université catholique de Louvain (UCLouvain); NBTF, National Biobank of Thailand, Pathum Thani, Thailand; STMA, HZI culture collection, Helmholtz Centre for Infection Research, Braunschweig, Germany.

## Data Availability

All newly generated sequences have been submitted to the public domain as GenBank.

## Supplementary Materials

The following supporting information can be downloaded at https://www.mdpi.com/article/10.3390/jof9040429/s1, Figure S1: Phylogeny of the Hypoxylaceae. The phylogenetic relationships inferred from Bayesian analysis based on multiple genetic loci of nuclear ribosomal LSU and proteinogenic (*TUB2* and *RPB2*) sequences**.** Support values of more than 50% (MPBS/MLBS) or 95 (BPP) were calculated via MP, ML, and Bayesian analysis and are indicated above (MPBS/MLBS) and below (BPP) the respective branches. Branches of significant support (MPBS, MLBS = 70% and BPP = 95) are thickened. New species are indicated in blue and the clade comprising the sequences of the *Pyrenopolyporus* spp. which is marked by a grey rectangle, and ET indicates ex-epitype, HT ex-holotype, and PT ex-paratype strains; Figure S2: Phylogeny of the Hypoxylaceae. The phylogenetic relationships depicted as maximum parsimony tree generated based on ITS sequences. Support values of more than 50% (MPBS/MLBS) or 95 (BPP) were calculated via MP, ML, and Bayesian analysis and are indicated above (MPBS/MLBS) and below (BPP) the respective branches. Branches of significant support (MPBS, MLBS = 70% and BPP = 95) are thickened. New species are indicated in blue and the clade comprising the sequences of the *Pyrenopolyporus* spp. is marked by a grey rectangle, and ET indicates ex-epitype, HT ex-holotype, and PT ex-paratype strains; Figure S3: Phylogeny of the Hypoxylaceae. The phylogenetic relationships depicted as maximum parsimony tree are generated based on LSU sequences. Support values of more than 50% (MPBS/MLBS) or 95 (BPP) were calculated via MP, ML, and Bayesian analysis and are indicated above (MPBS/MLBS) and below (BPP) the respective branches. Branches of significant support (MPBS, MLBS =70% and BPP = 95) are thickened. New species are indicated in blue and the clade comprising the sequences of the *Pyrenopolyporus* spp. is marked by a grey rectangle, and ET indicates ex-epitype, HT ex-holotype, and PT ex-paratype strains; Figure S4: Phylogeny of the Hypoxylaceae. The phylogenetic relationships inferred from Bayesian analysis based on proteinogenic (*RPB2)* sequences**.** Support values of more than 50% (MPBS/MLBS) or 95 (BPP) were calculated via MP, ML, and Bayesian analysis and are indicated above (MPBS/MLBS) and below (BPP) the respective branches. Branches of significant support (MPBS, MLBS = 70% and BPP = 95) are thickened. New species are indicated in blue and the clade comprising the sequences of the *Pyrenopolyporus* spp. is marked by a grey rectangle, and ET indicates ex-epitype, HT ex-holotype, and PT ex-paratype strains; Figure S5: Phylogeny of the Hypoxylaceae. The phylogenetic relationships inferred from Bayesian analysis based on proteinogenic (*TUB2)* sequences**.** Support values of more than 50% (MPBS/MLBS) or 95 (BPP) were calculated via MP, ML, and Bayesian analysis and are indicated above (MPBS/MLBS) and below (BPP) the respective branches. Branches of significant support (MPBS, MLBS = 70% and BPP = 95) are thickened. New species are indicated in blue and the clade comprising the sequences of the *Pyrenopolyporus* spp. which is marked by a grey rectangle, and ET indicates ex-epitype, HT ex-holotype, and PT ex-paratype strains; Figure S6: MALDI-TOF MASS SPECTRA; Figure S7: DAD spectra of the major metabolites (1−5) depicted in the stromata of the evaluated *Pyrenopolyporus* spp.; Figure S8: MS/MS spectra comparison between hypoxylone (1) and compound **3**. The MS/MS similarity score between the two metabolites is <500, but both spectra share analogous neutral losses for the major fragment ions; Table S1: Comparison of morphological and chemotaxonomic features of *Hypoxylaceae* species with massive stroma and long tubular perithecia; Table S2: Dereplicated metabolites from the stromatal extracts of the *Pyrenopolyporus* spp. and in-house standards.
