# Supplementary material for: Studies on the Genus Pyrenopolyporus (Hypoxylaceae) in Thailand Using a Polyphasic Taxonomic Approach
Source: J Fungi (Basel). 2023 Mar 30;9(4):429. doi: 10.3390/jof9040429 (PMC10145029; doi:10.3390/jof9040429)
Supplement: Supplementary file 1 [file jof-09-00429-s001.zip › jof-2228465-supplementary.pdf]

Article

Studies on the genus *Pyrenopolyporus* (Hypoxylaceae) in Thailand using a polyphasic taxonomic approach

Sarunyou Wongkanoun [1], Boonchuai Chainuwong [1], Noppol Kobmoo [2], Sittiruk Roytrakul [3], Sayanh Somrithipol [2], Jennifer Luangsa-ard [2], Esteban Charria-Girón [4,5], Prasert Srikitikulchai [1,*] and Marc Stadler [4,5,*]

[1] National Biobank of Thailand (NBT), National Science and Technology Development Agency (NSTDA), 111 Thailand Science Park, Phahonyothin Road, Khlong Nueng, Khlong Luang, Pathum Thani 12120, Thailand; sarunyou.won@nstda.or.th (S.W.)

[2] Plant Microbe Interaction Research Team (APMT), Integrative Crop Biotechnology and Management Research Group, National Center for Genetic Engineering and Biotechnology (BIOTEC), 113 Thailand Science Park, Phahonyothin Road, Khlong Nueng, Khlong Luang, Pathum Thani 12120, Thailand; noppol.kob@biotec.or.th (N.K.)

[3] Functional Proteomics Technology ((IFPT), Functional Ingredients and Food Innovation Research Group, National Center for Genetic Engineering and Biotechnology, 113 Thailand Science Park, Phahonyothin Road, Khlong Nueng, Khlong Luang 12120, Pathum Thani, Thailand

[4] Department of Microbial Drugs, Helmholtz Centre for Infection Research GmbH (HZI), and German Centre for Infection Research Association (DZIF), Hannover-Braunschweig, Inhoffenstraße 7, Braunschweig 38124, Germany

[5] Institute of Microbiology, Technische Universität Braunschweig, Spielmannstraße 7, Braunschweig 38106, Germany

* Correspondence: prasert@nstda.or.th (P.S.); marc.stadler@helmholtz-hzi.de (M.S.)

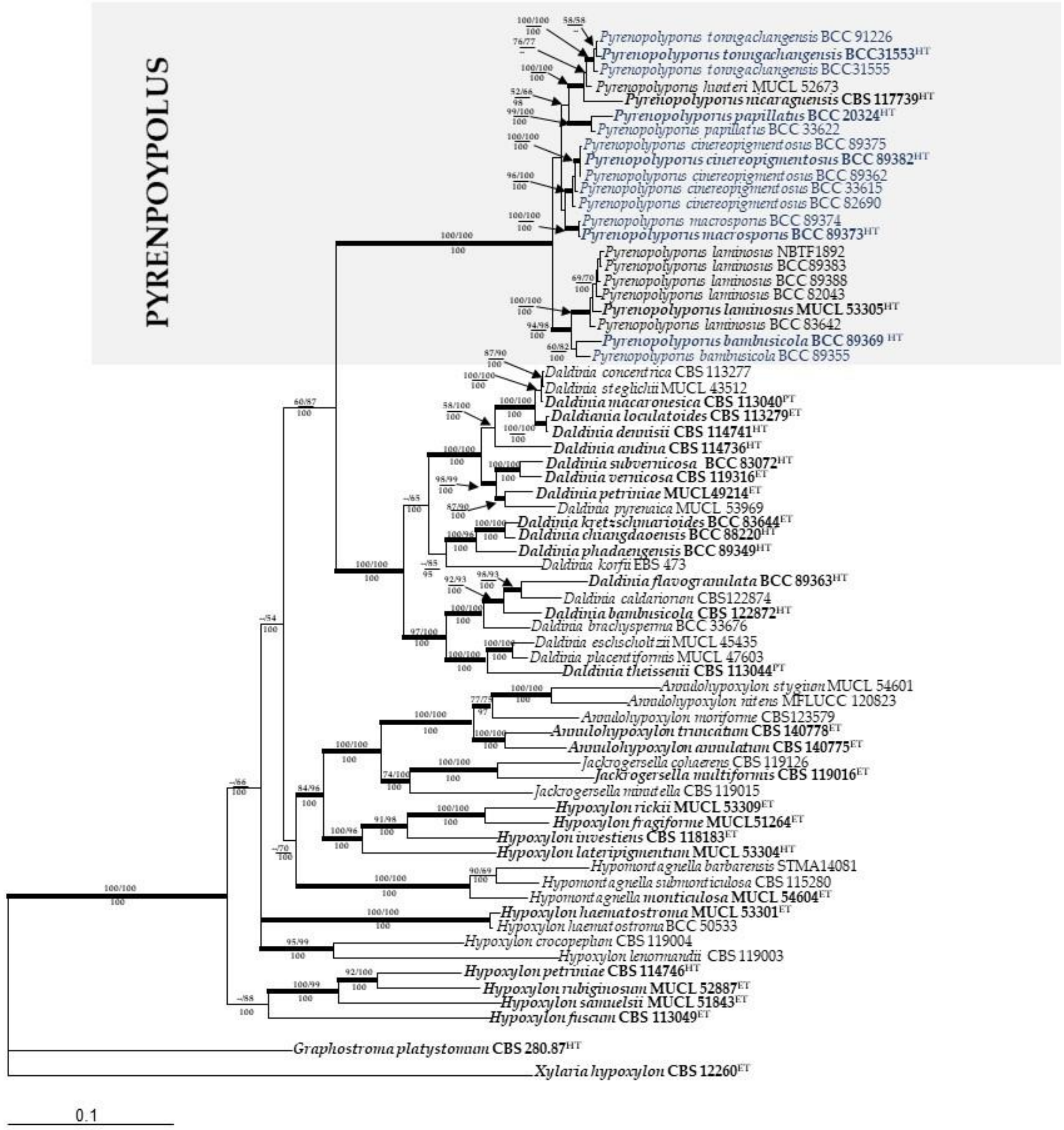

Figure S1. Phylogeny of the Hypoxylaceae. The phylogenetic relationships inferred from Bayesian analysis based on multiple genetic loci of nuclear ribosomal LSU and proteinogenic (*TUB2* and *RPB2*) sequences**.** Support values of more than 50% (MPBS/MLBS) or 0.95 (BPP) were calculated via MP, ML and Bayesian analysis and are indicated above (MPBS/MLBS) and below (BPP) the respective branches. Branches of significant support (MPBS, MLBS =70% and BPP =95) are thickened. New species are indicated in blue and the clade comprising the sequences of the *Pyrenopolyporus* spp. is marked by a grey rectangle, ET indicates ex-epitype, HT ex-holotype, and PT ex-paratype strains.

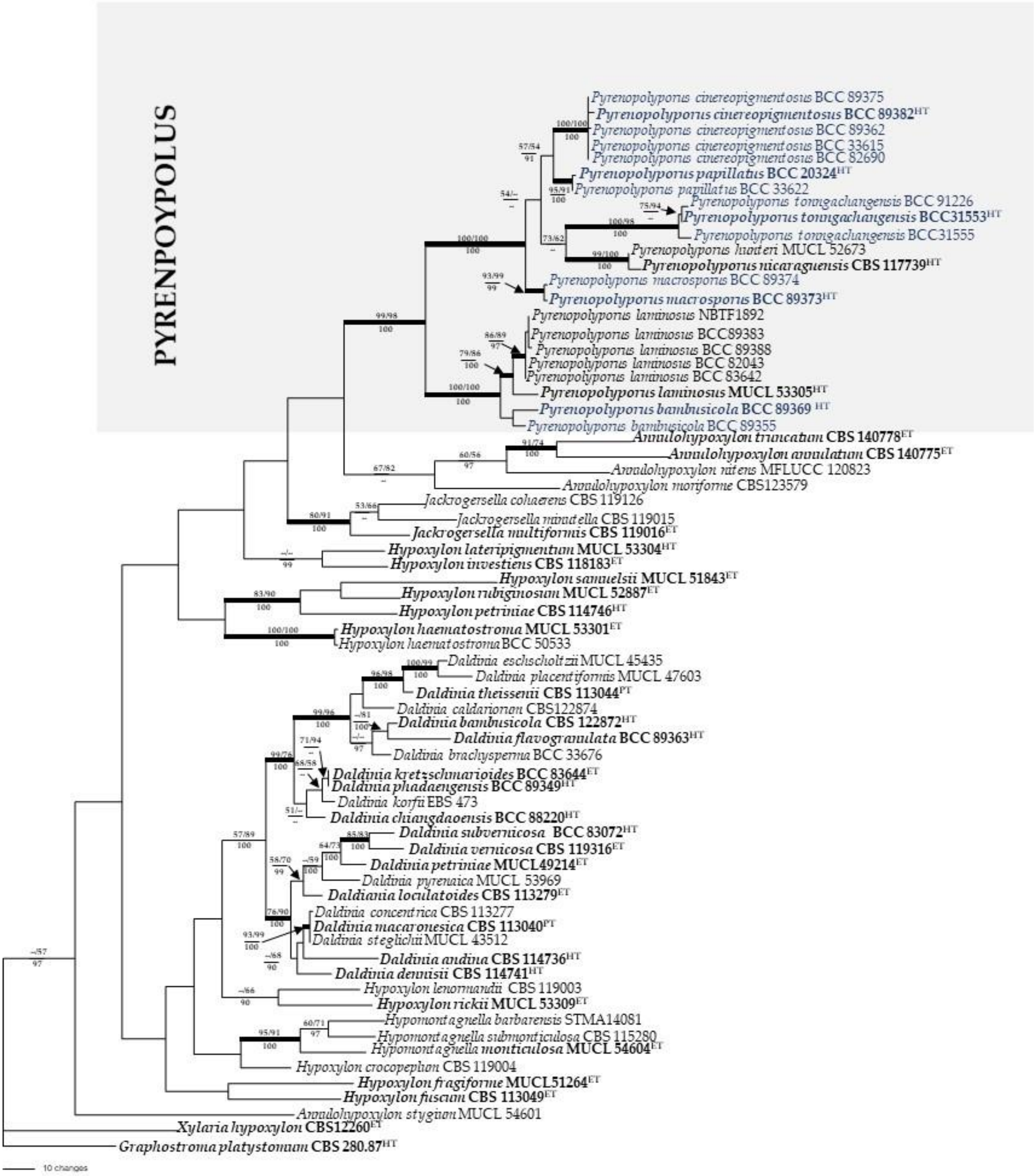

Figure S2. Phylogeny of the Hypoxylaceae. The phylogenetic relationships depicted as Maximum parsimony tree generated based on ITS sequences. Support values of more than 50% (MPBS/MLBS) or 95 (BPP) were calculated via MP, ML and Bayesian analysis and are indicated above (MPBS/MLBS) and below (BPP) the respective branches. Branches of significant support (MPBS, MLBS =70% and BPP =95) are thickened. New species are indicated in blue and the clade comprising the sequences of the *Pyrenopolyporus* spp. is marked by a grey rectangle, ET indicates ex-epitype, HT ex-holotype, and PT ex-paratype strains.

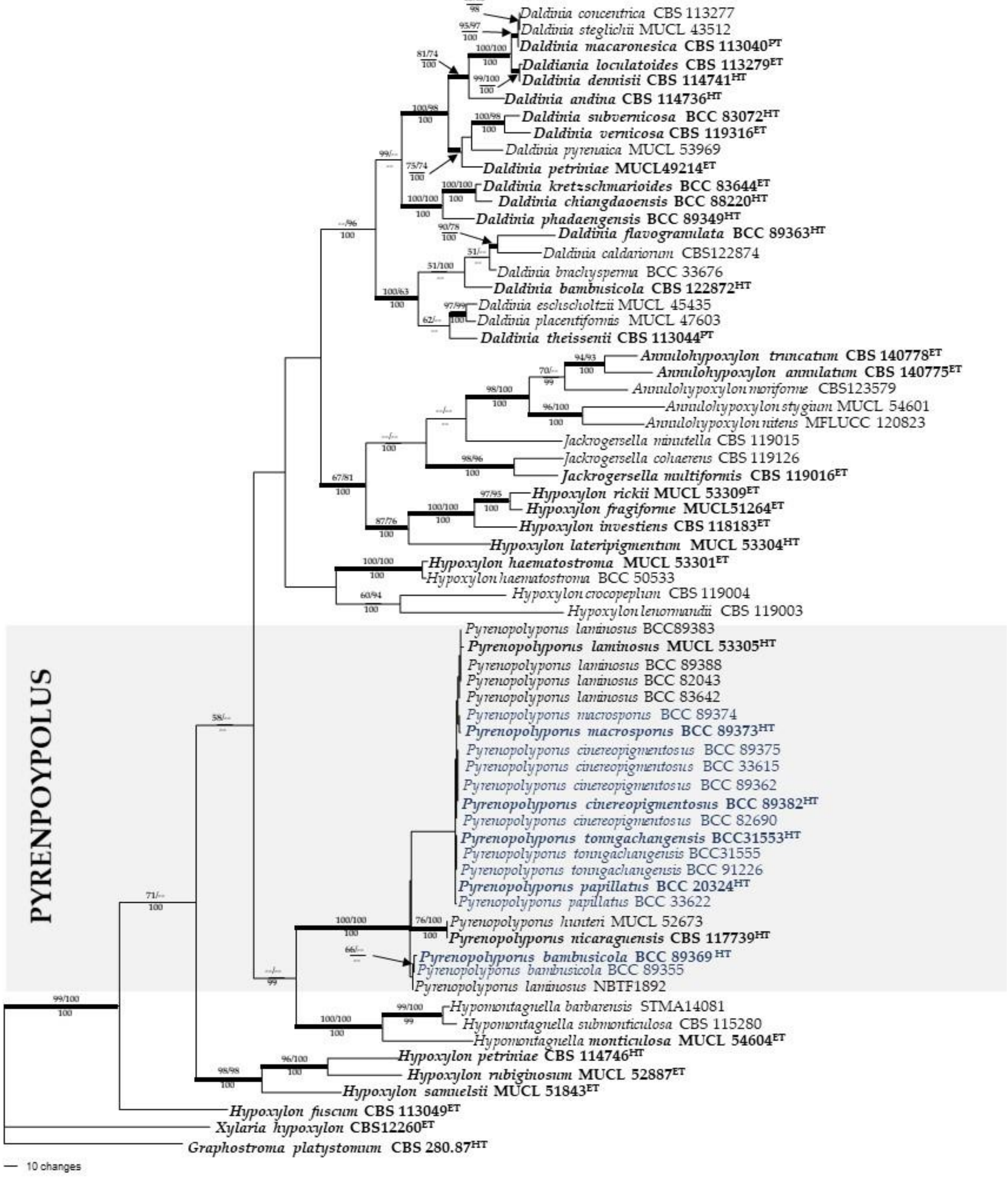

Figure S3. Phylogeny of the Hypoxylaceae. The phylogenetic relationships depicted as Maximum parsimony tree generated based on LSU sequences. Support values of more than 50% (MPBS/MLBS) or 95 (BPP) were calculated via MP, ML and Bayesian analysis and are indicated above (MPBS/MLBS) and below (BPP) the respective branches. Branches of significant support (MPBS, MLBS =70% and BPP =95) are thickened. New species are indicated in blue and the clade comprising the sequences of the *Pyrenopolyporus* spp. is marked by a grey rectangle, ET indicates ex-epitype, HT ex-holotype, and PT ex-paratype strains.

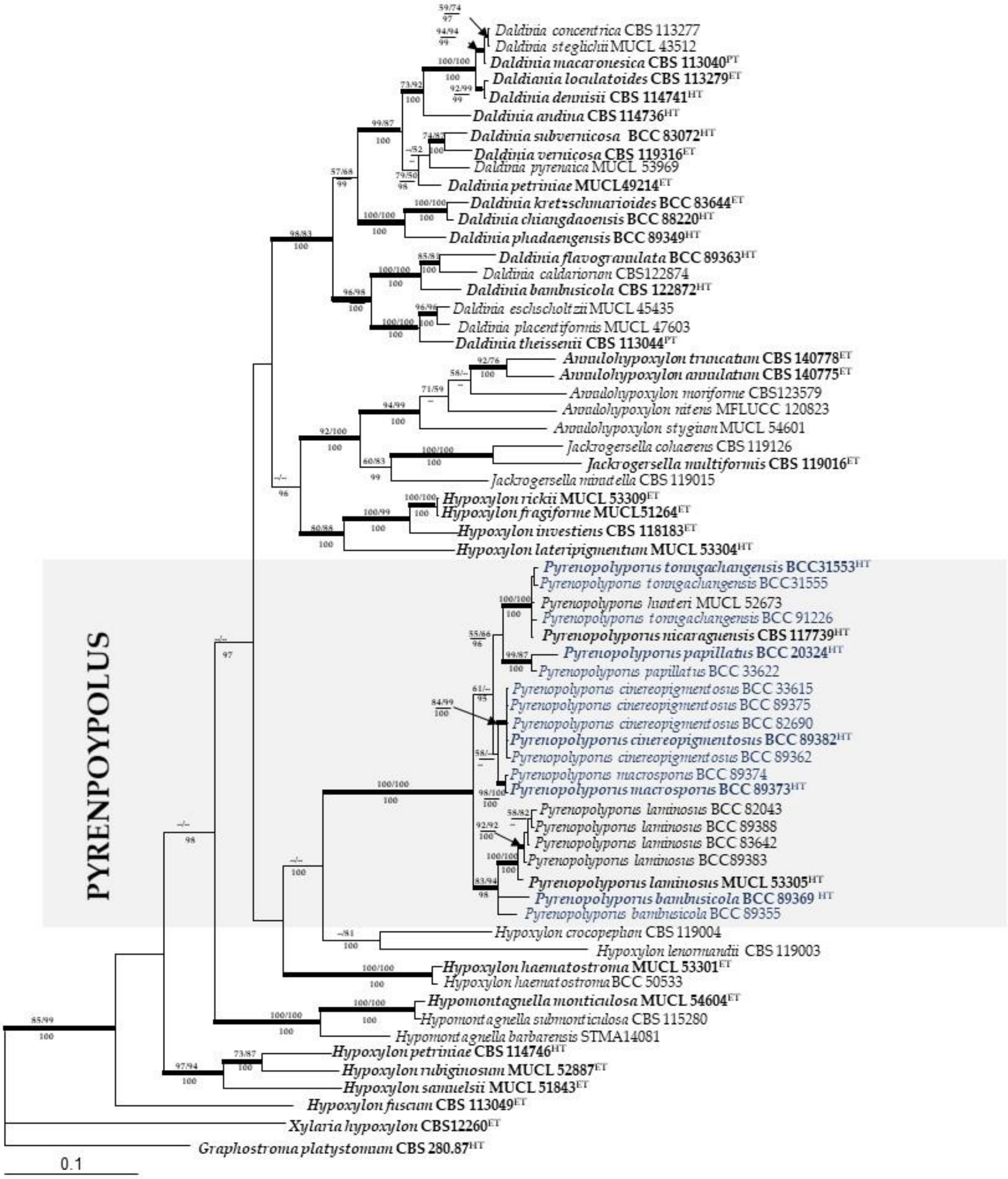

Figure S4. Phylogeny of the Hypoxylaceae. The phylogenetic relationships inferred from Bayesian analysis based on proteinogenic (*RPB2)* sequences**.** Support values of more than 50% (MPBS/MLBS) or 0.95 (BPP) were calculated via MP, ML and Bayesian analysis and are indicated above (MPBS/MLBS) and below (BPP) the respective branches. Branches of significant support (MPBS, MLBS =70% and BPP =95) are thickened. New species are indicated in blue and the clade comprising the sequences of the *Pyrenopolyporus* spp. is marked by a grey rectangle, ET indicates ex-epitype, HT ex-holotype, and PT ex-paratype strains.

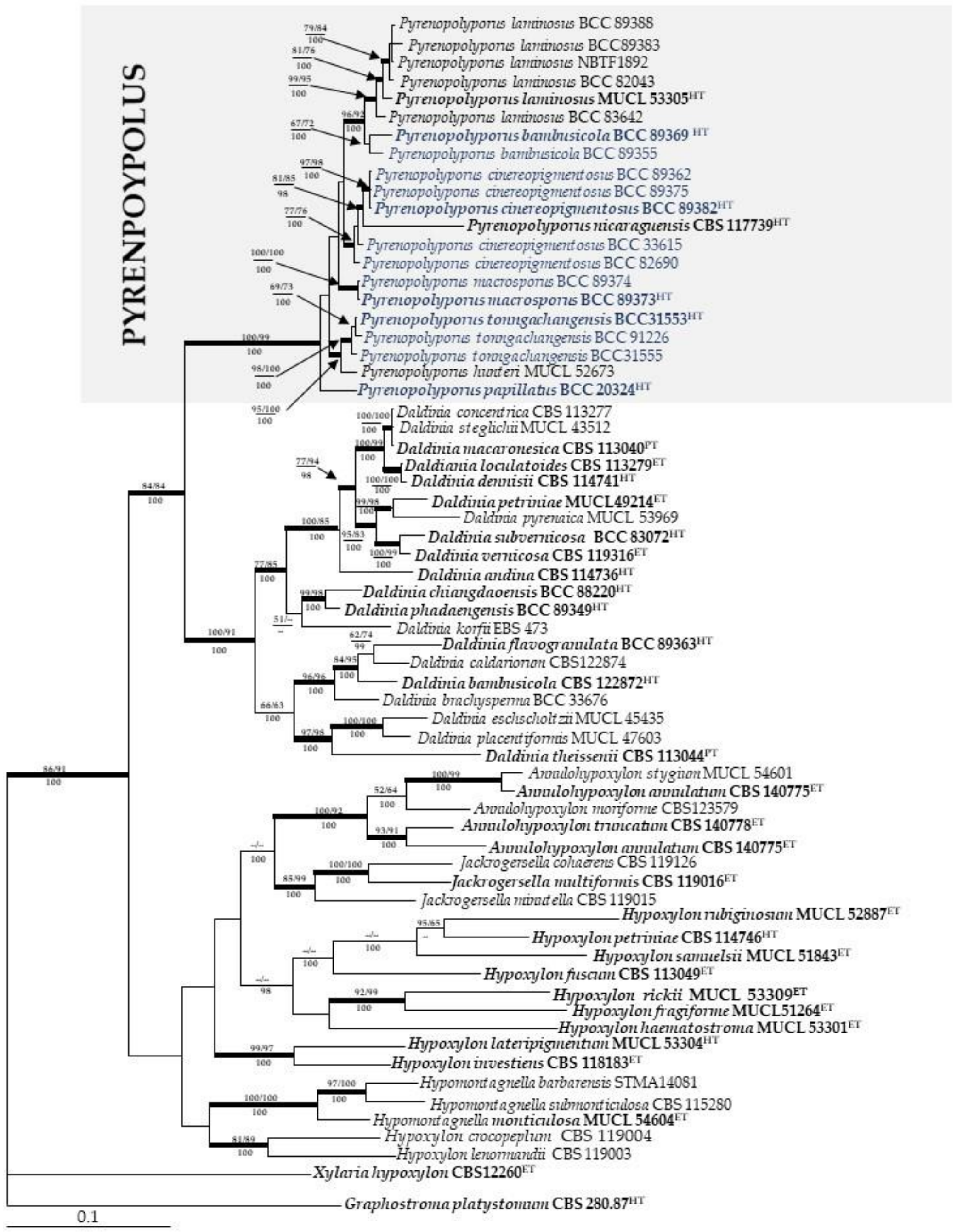

Figure S5. Phylogeny of the Hypoxylaceae. The phylogenetic relationships inferred from Bayesian analysis based on proteinogenic (*TUB2)* sequences**.** Support values of more than 50% (MPBS/MLBS) or 0.95 (BPP) were calculated via MP, ML and Bayesian analysis and are indicated above (MPBS/MLBS) and below (BPP) the respective branches. Branches of significant support (MPBS, MLBS =70% and BPP =95) are thickened. New species are indicated in blue and the clade comprising the sequences of the *Pyrenopolyporus* spp. is marked by a grey rectangle, ET indicates ex-epitype, HT ex-holotype, and PT ex-paratype strains.

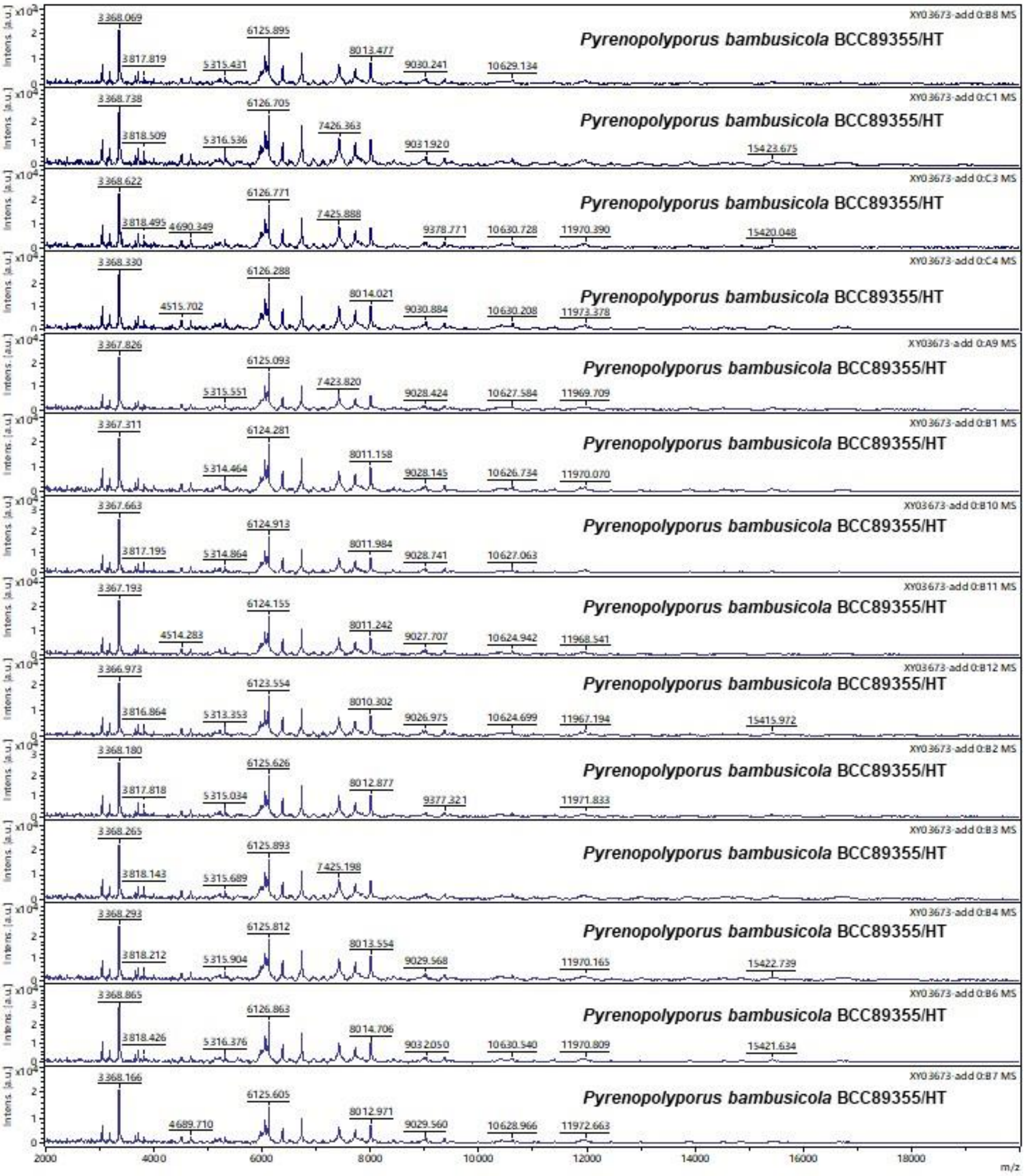

XY03673-add 0:B8 MS
Pyrenopolyporus bambusicola BCC89355/HT
3368.069
3817.819
5315.431
6125.895
8013.477
9030.241
10629.134
XY03673-add 0:C1 MS
Pyrenopolyporus bambusicola BCC89355/HT
3368.738
3818.509
5316.536
6126.705
7426.363
9031.920
15423.675
XY03673-add 0:C3 MS
Pyrenopolyporus bambusicola BCC89355/HT
3368.622
3818.495
4690.349
6126.771
7425.888
9378.771
10630.728
11970.390
15420.048
XY03673-add 0:C4 MS
Pyrenopolyporus bambusicola BCC89355/HT
3368.330
4515.702
6126.288
8014.021
9030.884
10630.208
11973.378
XY03673-add 0:A9 MS
Pyrenopolyporus bambusicola BCC89355/HT
3367.826
5315.551
6125.093
7423.820
9028.424
10627.584
11969.709
XY03673-add 0:B1 MS
Pyrenopolyporus bambusicola BCC89355/HT
3367.311
5314.464
6124.281
8011.158
9028.145
10626.734
11970.070
XY03673-add 0:B10 MS
Pyrenopolyporus bambusicola BCC89355/HT
3367.663
3817.195
5314.864
6124.913
8011.984
9028.741
10627.063
XY03673-add 0:B11 MS
Pyrenopolyporus bambusicola BCC89355/HT
3367.193
4514.283
6124.155
8011.242
9027.707
10624.942
11968.541
XY03673-add 0:B12 MS
Pyrenopolyporus bambusicola BCC89355/HT
3366.973
3816.864
5313.353
6123.554
8010.302
9026.975
10624.699
11967.194
15415.972
XY03673-add 0:B2 MS
Pyrenopolyporus bambusicola BCC89355/HT
3368.180
3817.818
5315.034
6125.626
8012.877
9377.321
11971.833
XY03673-add 0:B3 MS
Pyrenopolyporus bambusicola BCC89355/HT
3368.265
3818.143
5315.689
6125.893
7425.198
XY03673-add 0:B4 MS
Pyrenopolyporus bambusicola BCC89355/HT
3368.293
3818.212
5315.904
6125.812
8013.554
9029.568
11970.165
15422.739
XY03673-add 0:B6 MS
Pyrenopolyporus bambusicola BCC89355/HT
3368.865
3818.426
5316.376
6126.863
8014.706
9032.050
10630.540
11970.809
15421.634
XY03673-add 0:B7 MS
Pyrenopolyporus bambusicola BCC89355/HT
3368.166
4689.710
6125.605
8012.971
9029.560
10628.966
11972.663
Intens. [a.u.]
x10^4
2000
4000
6000
8000
10000
12000
14000
16000
18000
m/z

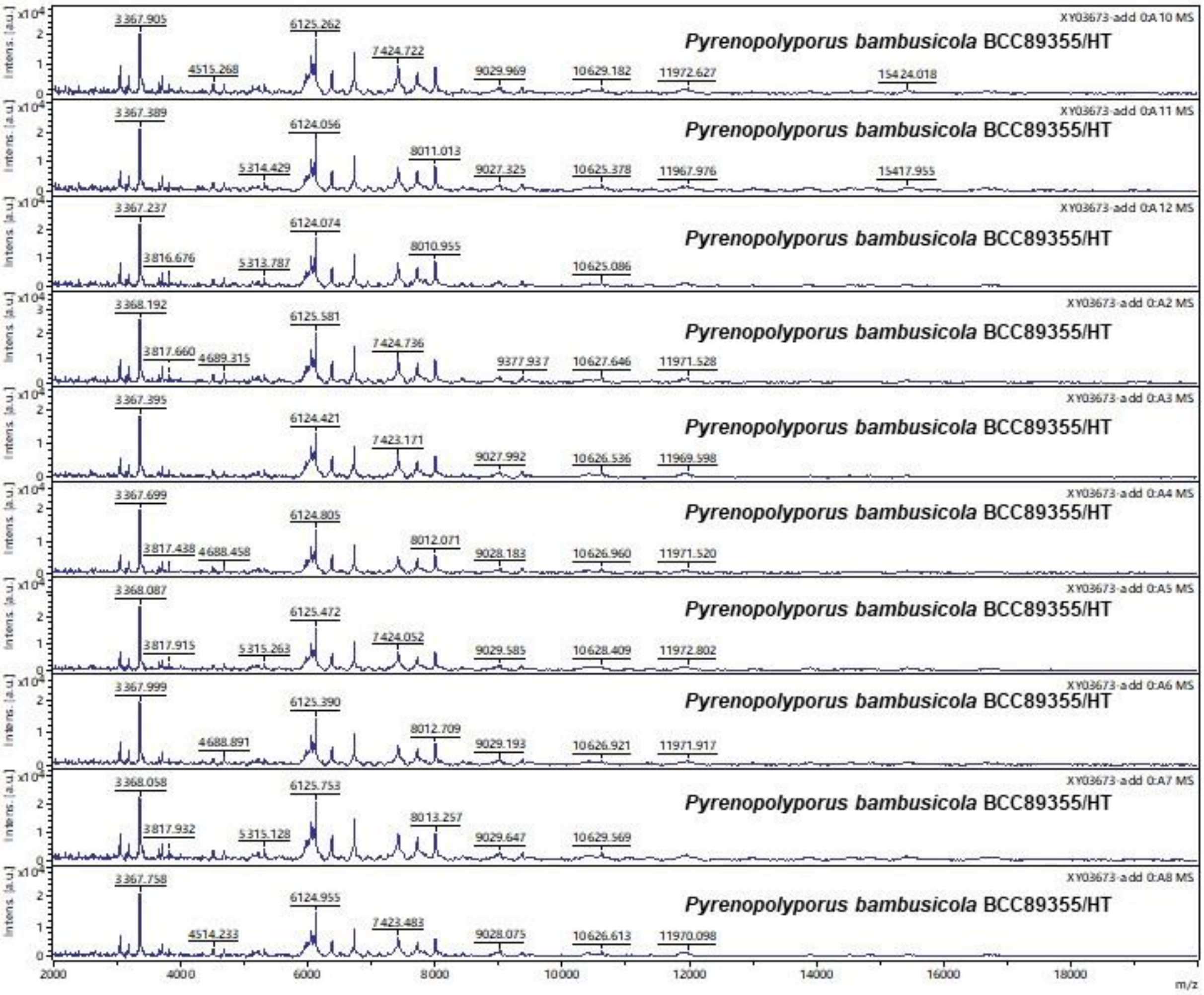
XY03673-add 0:A10 MS
Pyrenopolyporus bambusicola BCC89355/HT
3367.905
4515.268
6125.262
7424.722
9029.969
10629.182
11972.627
15424.018
XY03673-add 0:A11 MS
Pyrenopolyporus bambusicola BCC89355/HT
3367.389
5314.429
6124.056
8011.013
9027.325
10625.378
11967.976
15417.955
XY03673-add 0:A12 MS
Pyrenopolyporus bambusicola BCC89355/HT
3367.237
3816.676
5313.787
6124.074
8010.955
10625.086
XY03673-add 0:A2 MS
Pyrenopolyporus bambusicola BCC89355/HT
3368.192
3817.660
4689.315
6125.581
7424.736
9377.937
10627.646
11971.528
XY03673-add 0:A3 MS
Pyrenopolyporus bambusicola BCC89355/HT
3367.395
6124.421
7423.171
9027.992
10626.536
11969.598
XY03673-add 0:A4 MS
Pyrenopolyporus bambusicola BCC89355/HT
3367.699
3817.438
4688.458
6124.805
8012.071
9028.183
10626.960
11971.520
XY03673-add 0:A5 MS
Pyrenopolyporus bambusicola BCC89355/HT
3368.087
3817.915
5315.263
6125.472
7424.052
9029.585
10628.409
11972.802
XY03673-add 0:A6 MS
Pyrenopolyporus bambusicola BCC89355/HT
3367.999
4688.891
6125.390
8012.709
9029.193
10626.921
11971.917
XY03673-add 0:A7 MS
Pyrenopolyporus bambusicola BCC89355/HT
3368.058
3817.932
5315.128
6125.753
8013.257
9029.647
10629.569
XY03673-add 0:A8 MS
Pyrenopolyporus bambusicola BCC89355/HT
3367.758
4514.233
6124.955
7423.483
9028.075
10626.613
11970.098
Intens. [a.u.]
x10^4
2000
4000
6000
8000
10000
12000
14000
16000
18000
m/z

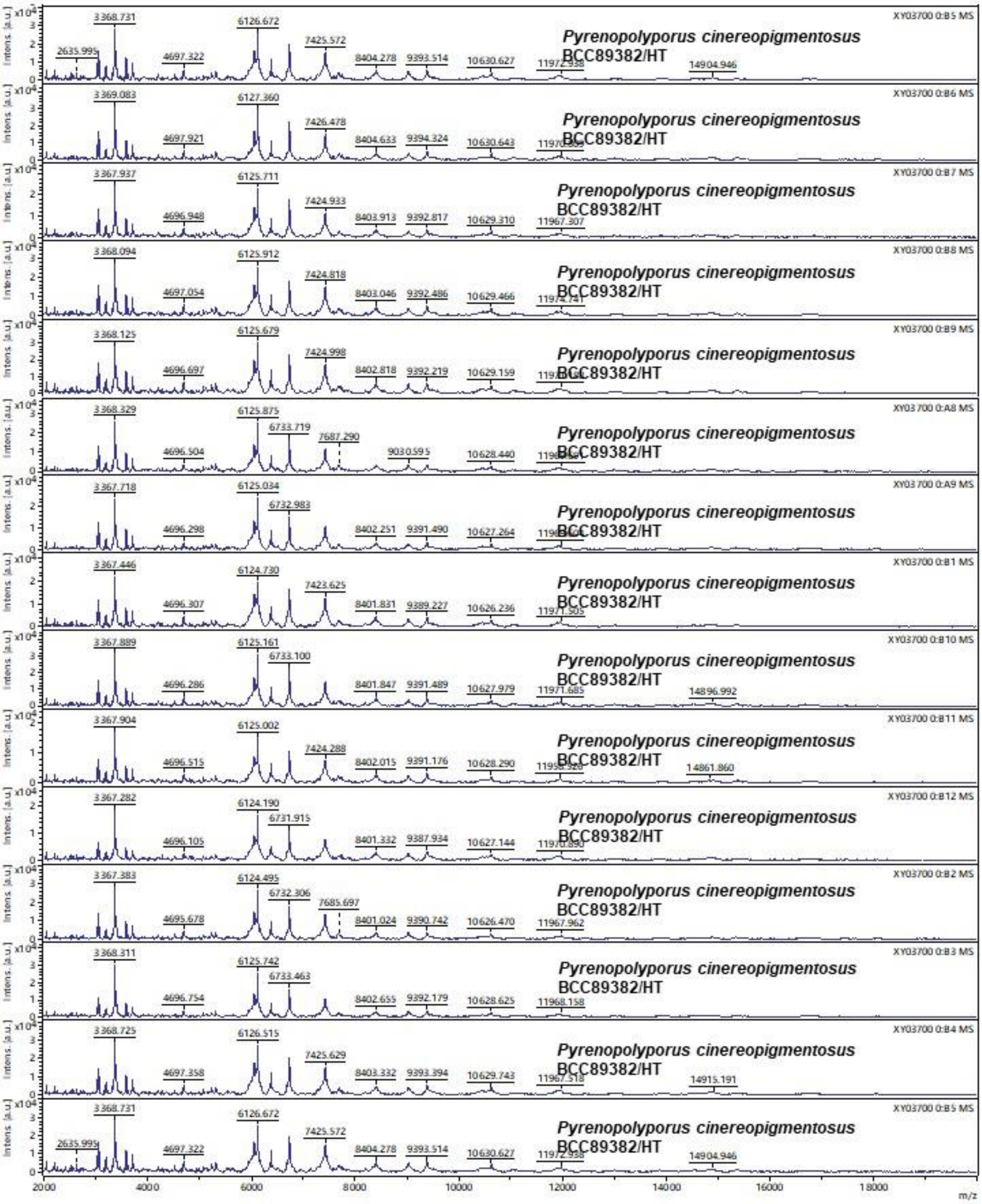
Pyrenopolyporus cinereopigmentosus
BCC89382/HT
m/z

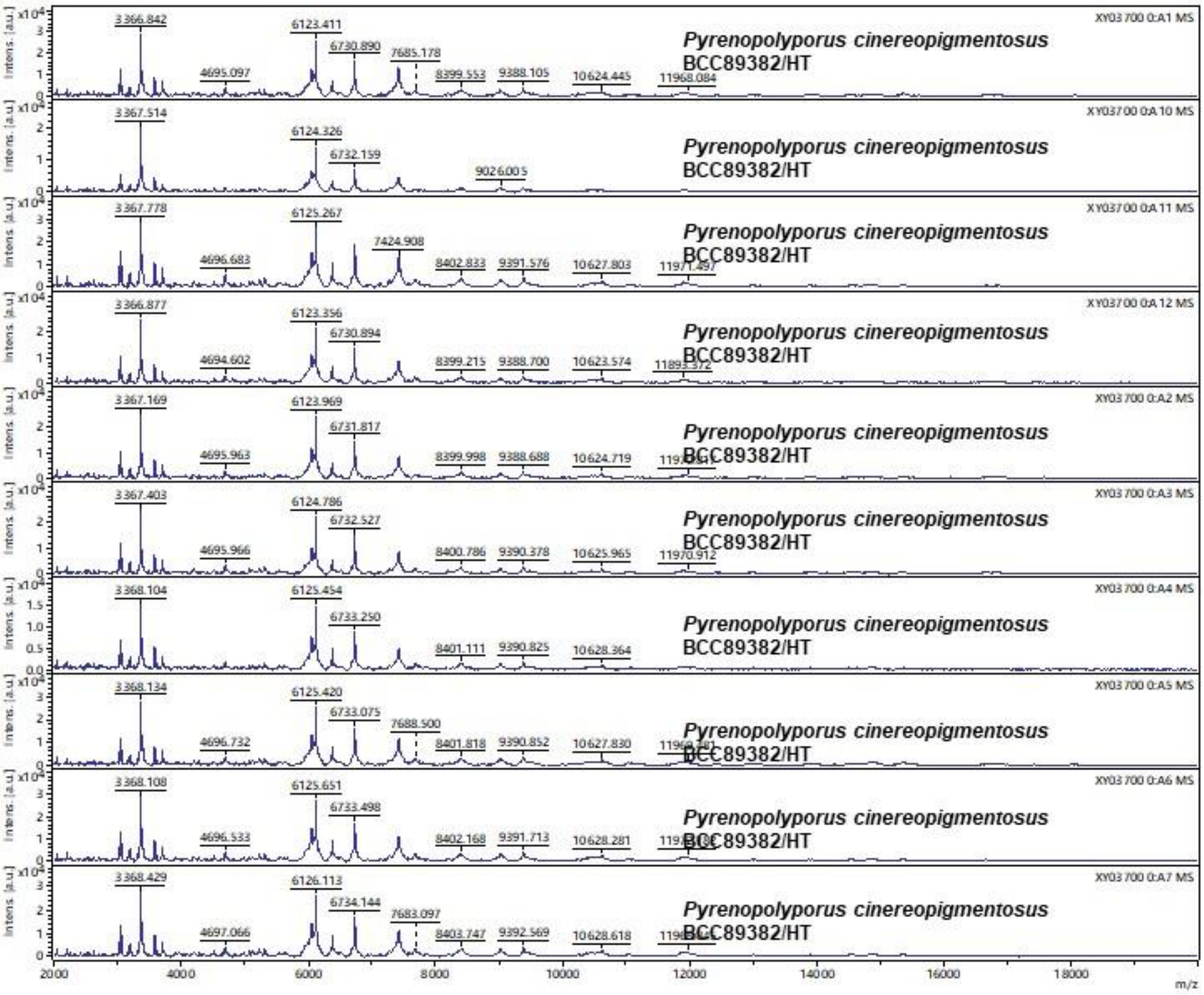
XY03700 0:A1 MS
XY03700 0:A10 MS
XY03700 0:A11 MS
XY03700 0:A12 MS
XY03700 0:A2 MS
XY03700 0:A3 MS
XY03700 0:A4 MS
XY03700 0:A5 MS
XY03700 0:A6 MS
XY03700 0:A7 MS
Pyrenopolyporus cinereopigmentosus
BCC89382/HT
Intens. [a.u.]
x10^4
m/z

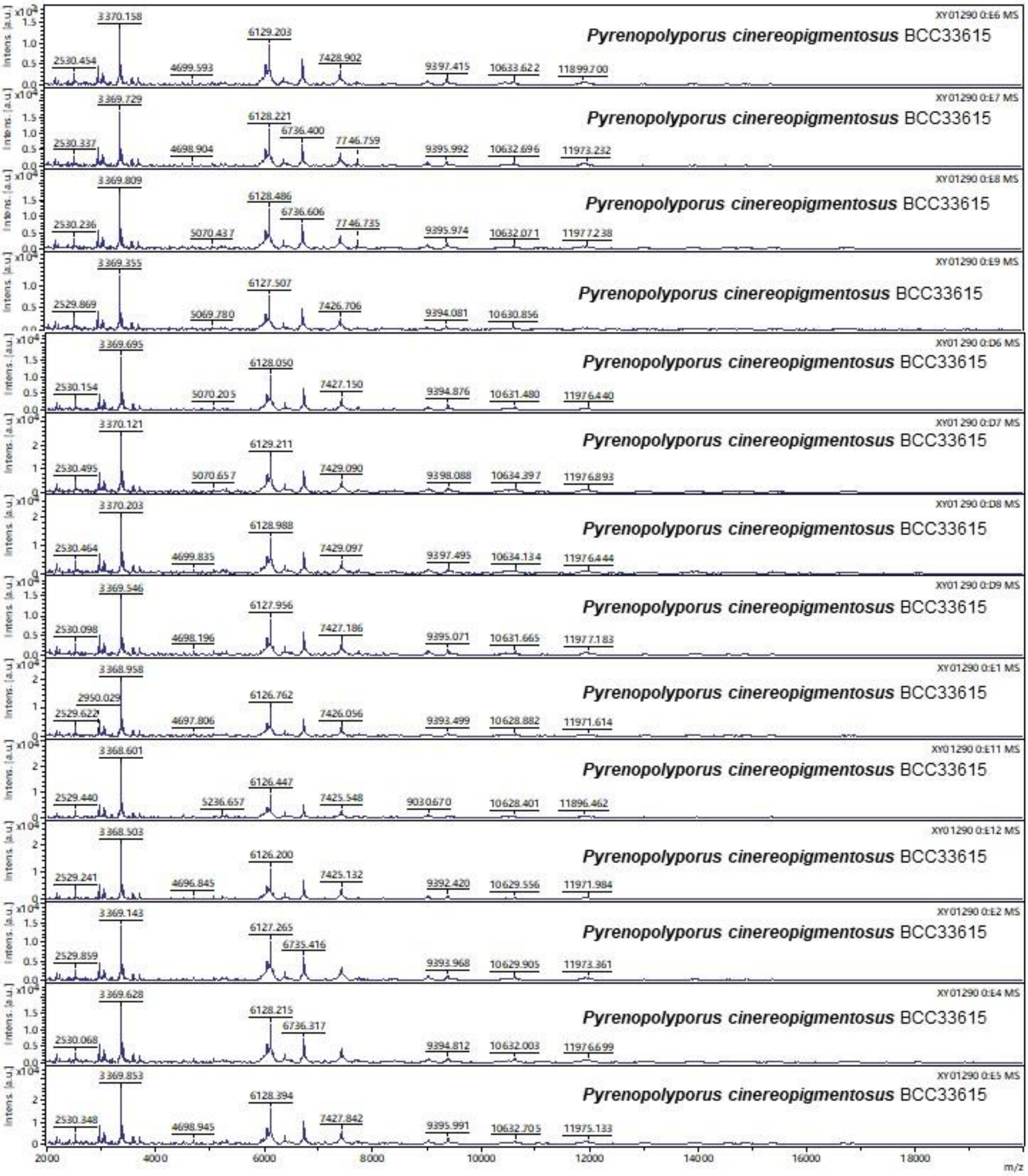

XY01290 0:E6 MS
Pyrenopolyporus cinereopigmentosus BCC33615
XY01290 0:E7 MS
Pyrenopolyporus cinereopigmentosus BCC33615
XY01290 0:E8 MS
Pyrenopolyporus cinereopigmentosus BCC33615
XY01290 0:E9 MS
Pyrenopolyporus cinereopigmentosus BCC33615
XY01290 0:D6 MS
Pyrenopolyporus cinereopigmentosus BCC33615
XY01290 0:D7 MS
Pyrenopolyporus cinereopigmentosus BCC33615
XY01290 0:D8 MS
Pyrenopolyporus cinereopigmentosus BCC33615
XY01290 0:D9 MS
Pyrenopolyporus cinereopigmentosus BCC33615
XY01290 0:E1 MS
Pyrenopolyporus cinereopigmentosus BCC33615
XY01290 0:E11 MS
Pyrenopolyporus cinereopigmentosus BCC33615
XY01290 0:E12 MS
Pyrenopolyporus cinereopigmentosus BCC33615
XY01290 0:E2 MS
Pyrenopolyporus cinereopigmentosus BCC33615
XY01290 0:E4 MS
Pyrenopolyporus cinereopigmentosus BCC33615
XY01290 0:E5 MS
Pyrenopolyporus cinereopigmentosus BCC33615
Intens. [a.u.]
m/z

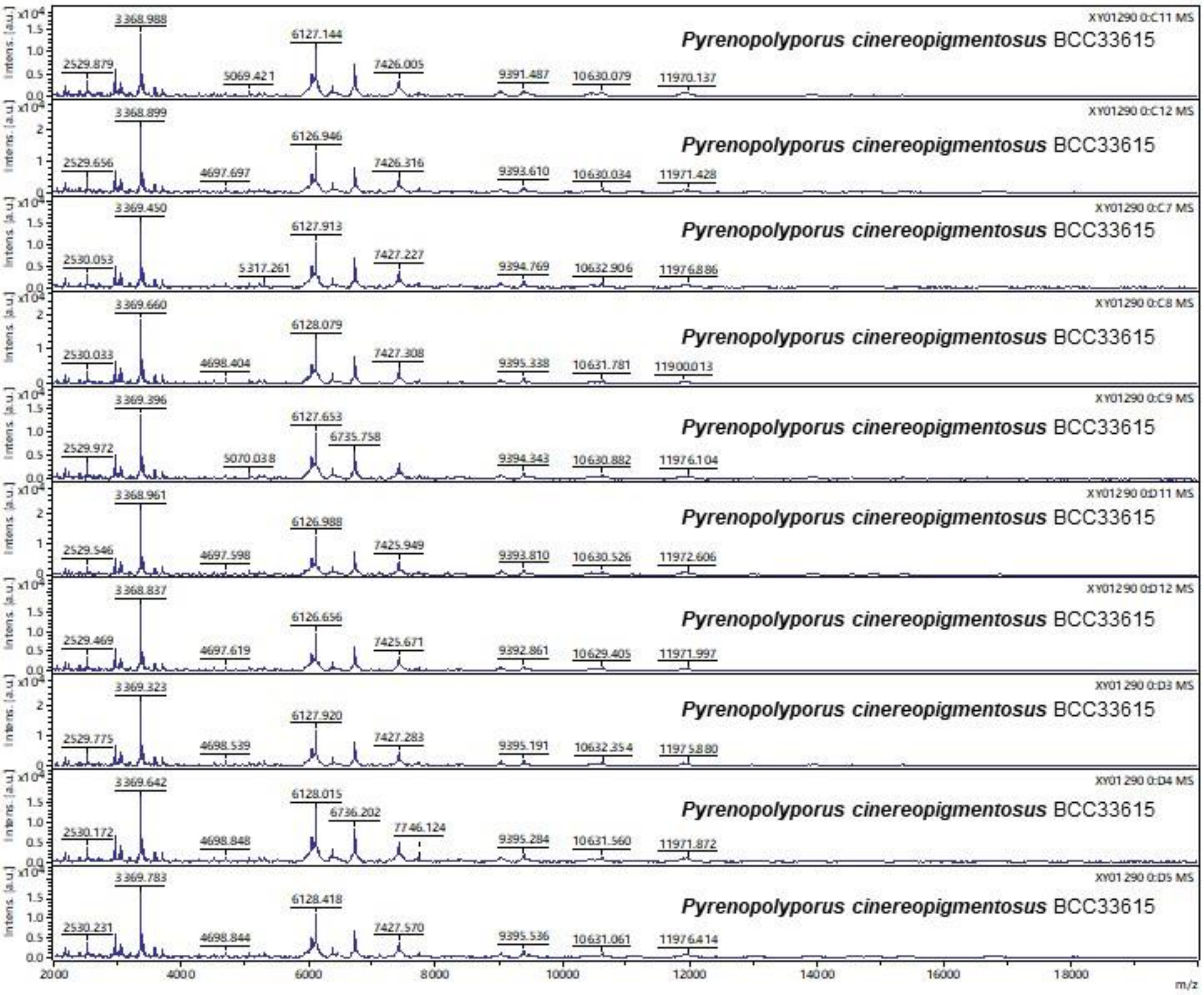

XY01290 0:C11 MS
Pyrenopolyporus cinereopigmentosus BCC33615
3368.988
2529.879
5069.421
6127.144
7426.005
9391.487
10630.079
11970.137
XY01290 0:C12 MS
Pyrenopolyporus cinereopigmentosus BCC33615
3368.899
2529.656
4697.697
6126.946
7426.316
9393.610
10630.034
11971.428
XY01290 0:C7 MS
Pyrenopolyporus cinereopigmentosus BCC33615
3369.450
2530.053
5317.261
6127.913
7427.227
9394.769
10632.906
11976.886
XY01290 0:C8 MS
Pyrenopolyporus cinereopigmentosus BCC33615
3369.660
2530.033
4698.404
6128.079
7427.308
9395.338
10631.781
11900.013
XY01290 0:C9 MS
Pyrenopolyporus cinereopigmentosus BCC33615
3369.396
2529.972
5070.038
6127.653
6735.758
9394.343
10630.882
11976.104
XY01290 0:D11 MS
Pyrenopolyporus cinereopigmentosus BCC33615
3368.961
2529.546
4697.598
6126.988
7425.949
9393.810
10630.526
11972.606
XY01290 0:D12 MS
Pyrenopolyporus cinereopigmentosus BCC33615
3368.837
2529.469
4697.619
6126.656
7425.671
9392.861
10629.405
11971.997
XY01290 0:D3 MS
Pyrenopolyporus cinereopigmentosus BCC33615
3369.323
2529.775
4698.539
6127.920
7427.283
9395.191
10632.354
11975.880
XY01290 0:D4 MS
Pyrenopolyporus cinereopigmentosus BCC33615
3369.642
2530.172
4698.848
6128.015
6736.202
7746.124
9395.284
10631.560
11971.872
XY01290 0:D5 MS
Pyrenopolyporus cinereopigmentosus BCC33615
3369.783
2530.231
4698.844
6128.418
7427.570
9395.536
10631.061
11976.414
Intens. [a.u.]
x10^4
2000
4000
6000
8000
10000
12000
14000
16000
18000
m/z

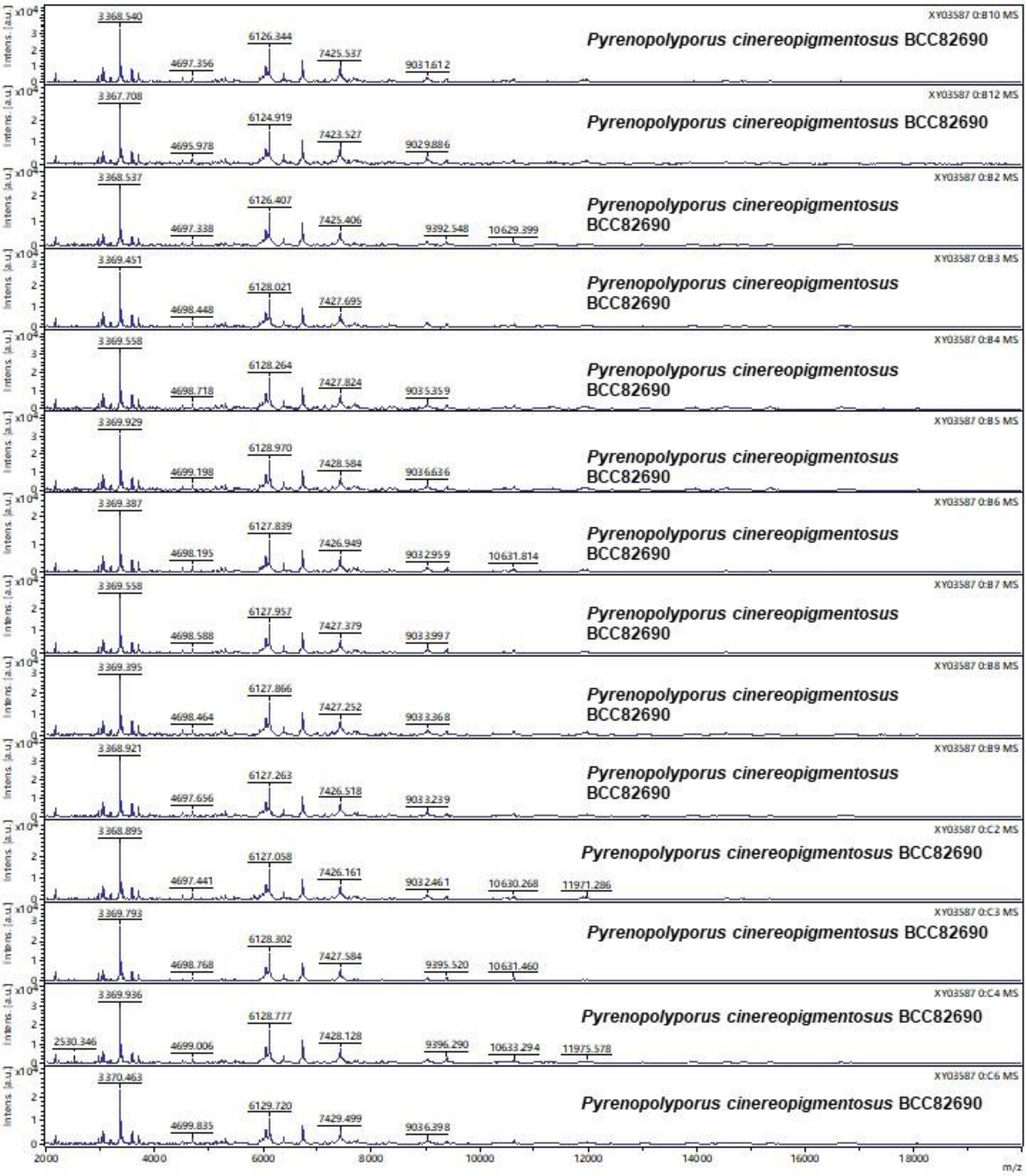
XY03587 0:B10 MS
Pyrenopolyporus cinereopigmentosus BCC82690
3368.540
4697.356
6126.344
7425.537
9031.612
XY03587 0:B12 MS
Pyrenopolyporus cinereopigmentosus BCC82690
3367.708
4695.978
6124.919
7423.527
9029.886
XY03587 0:B2 MS
Pyrenopolyporus cinereopigmentosus BCC82690
3368.537
4697.338
6126.407
7425.406
9392.548
10629.399
XY03587 0:B3 MS
Pyrenopolyporus cinereopigmentosus BCC82690
3369.451
4698.448
6128.021
7427.695
XY03587 0:B4 MS
Pyrenopolyporus cinereopigmentosus BCC82690
3369.558
4698.718
6128.264
7427.824
9035.359
XY03587 0:B5 MS
Pyrenopolyporus cinereopigmentosus BCC82690
3369.929
4699.198
6128.970
7428.584
9036.636
XY03587 0:B6 MS
Pyrenopolyporus cinereopigmentosus BCC82690
3369.387
4698.195
6127.839
7426.949
9032.959
10631.814
XY03587 0:B7 MS
Pyrenopolyporus cinereopigmentosus BCC82690
3369.558
4698.588
6127.957
7427.379
9033.997
XY03587 0:B8 MS
Pyrenopolyporus cinereopigmentosus BCC82690
3369.395
4698.464
6127.866
7427.252
9033.368
XY03587 0:B9 MS
Pyrenopolyporus cinereopigmentosus BCC82690
3368.921
4697.656
6127.263
7426.518
9033.239
XY03587 0:C2 MS
Pyrenopolyporus cinereopigmentosus BCC82690
3368.895
4697.441
6127.058
7426.161
9032.461
10630.268
11971.286
XY03587 0:C3 MS
Pyrenopolyporus cinereopigmentosus BCC82690
3369.793
4698.768
6128.302
7427.584
9395.520
10631.460
XY03587 0:C4 MS
Pyrenopolyporus cinereopigmentosus BCC82690
3369.936
2530.346
4699.006
6128.777
7428.128
9396.290
10633.294
11975.578
XY03587 0:C6 MS
Pyrenopolyporus cinereopigmentosus BCC82690
3370.463
4699.835
6129.720
7429.499
9036.398
Intens. [a.u.]
x10^4
m/z
2000
4000
6000
8000
10000
12000
14000
16000
18000

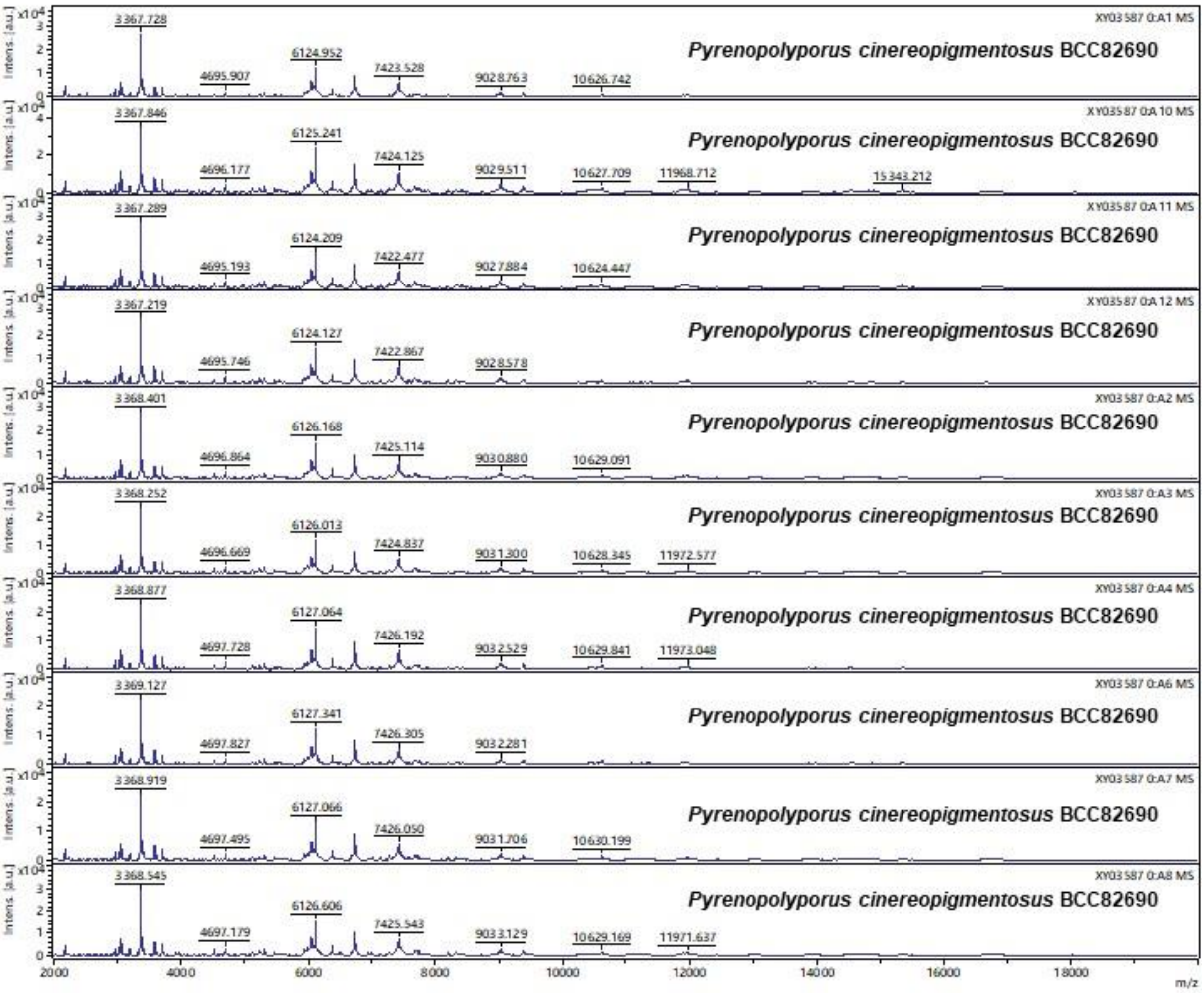

XY03587 0:A1 MS
Pyrenopolyporus cinereopigmentosus BCC82690
3367.728
4695.907
6124.952
7423.528
9028.763
10626.742
XY03587 0:A10 MS
Pyrenopolyporus cinereopigmentosus BCC82690
3367.846
4696.177
6125.241
7424.125
9029.511
10627.709
11968.712
15343.212
XY03587 0:A11 MS
Pyrenopolyporus cinereopigmentosus BCC82690
3367.289
4695.193
6124.209
7422.477
9027.884
10624.447
XY03587 0:A12 MS
Pyrenopolyporus cinereopigmentosus BCC82690
3367.219
4695.746
6124.127
7422.867
9028.578
XY03587 0:A2 MS
Pyrenopolyporus cinereopigmentosus BCC82690
3368.401
4696.864
6126.168
7425.114
9030.880
10629.091
XY03587 0:A3 MS
Pyrenopolyporus cinereopigmentosus BCC82690
3368.252
4696.669
6126.013
7424.837
9031.300
10628.345
11972.577
XY03587 0:A4 MS
Pyrenopolyporus cinereopigmentosus BCC82690
3368.877
4697.728
6127.064
7426.192
9032.529
10629.841
11973.048
XY03587 0:A6 MS
Pyrenopolyporus cinereopigmentosus BCC82690
3369.127
4697.827
6127.341
7426.305
9032.281
XY03587 0:A7 MS
Pyrenopolyporus cinereopigmentosus BCC82690
3368.919
4697.495
6127.066
7426.050
9031.706
10630.199
XY03587 0:A8 MS
Pyrenopolyporus cinereopigmentosus BCC82690
3368.545
4697.179
6126.606
7425.543
9033.129
10629.169
11971.637
Intens. [a.u.]
x10⁴
m/z
2000
4000
6000
8000
10000
12000
14000
16000
18000

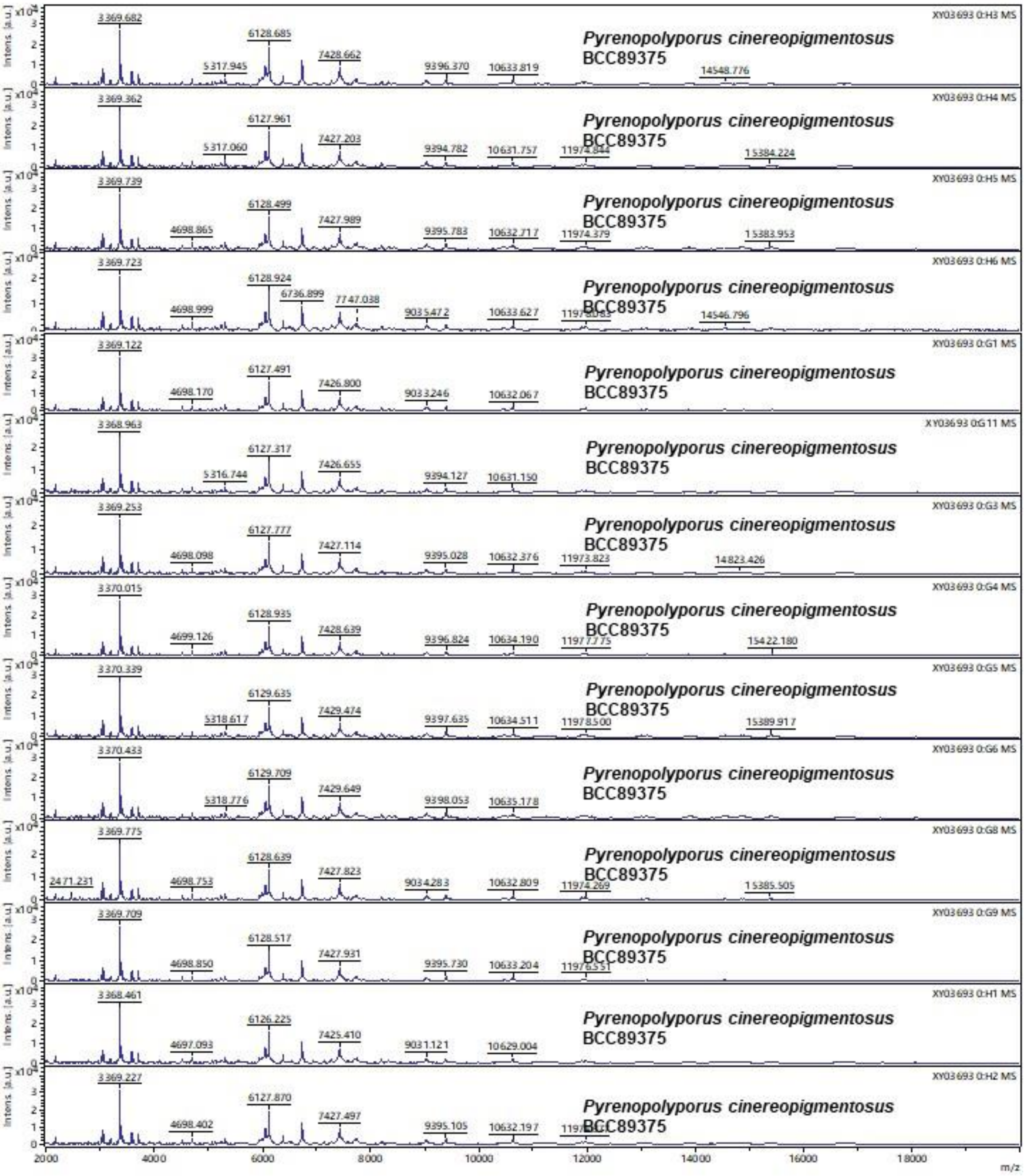
XY03693 0:H3 MS
Pyrenopolyporus cinereopigmentosus
BCC89375
3369.682
6128.685
7428.662
5317.945
9396.370
10633.819
14548.776
XY03693 0:H4 MS
3369.362
6127.961
7427.203
5317.060
9394.782
10631.757
15384.224
XY03693 0:H5 MS
3369.739
6128.499
7427.989
4698.865
9395.783
10632.717
11974.379
15383.953
XY03693 0:H6 MS
3369.723
6128.924
6736.899
7747.038
4698.999
9035.472
10633.627
14546.796
XY03693 0:G1 MS
3369.122
6127.491
7426.800
4698.170
9033.246
10632.067
XY03693 0:G11 MS
3368.963
6127.317
7426.655
5316.744
9394.127
10631.150
XY03693 0:G3 MS
3369.253
6127.777
7427.114
4698.098
9395.028
10632.376
11973.823
14823.426
XY03693 0:G4 MS
3370.015
6128.935
7428.639
4699.126
9396.824
10634.190
15422.180
XY03693 0:G5 MS
3370.339
6129.635
7429.474
5318.617
9397.635
10634.511
11978.500
15389.917
XY03693 0:G6 MS
3370.433
6129.709
7429.649
5318.776
9398.053
10635.178
XY03693 0:G8 MS
3369.775
6128.639
7427.823
2471.231
4698.753
9034.283
10632.809
11974.269
15385.505
XY03693 0:G9 MS
3369.709
6128.517
7427.931
4698.850
9395.730
10633.204
XY03693 0:H1 MS
3368.461
6126.225
7425.410
4697.093
9031.121
10629.004
XY03693 0:H2 MS
3369.227
6127.870
7427.497
4698.402
9395.105
10632.197
Intens. [a.u.]
x10^4
m/z
2000
4000
6000
8000
10000
12000
14000
16000
18000

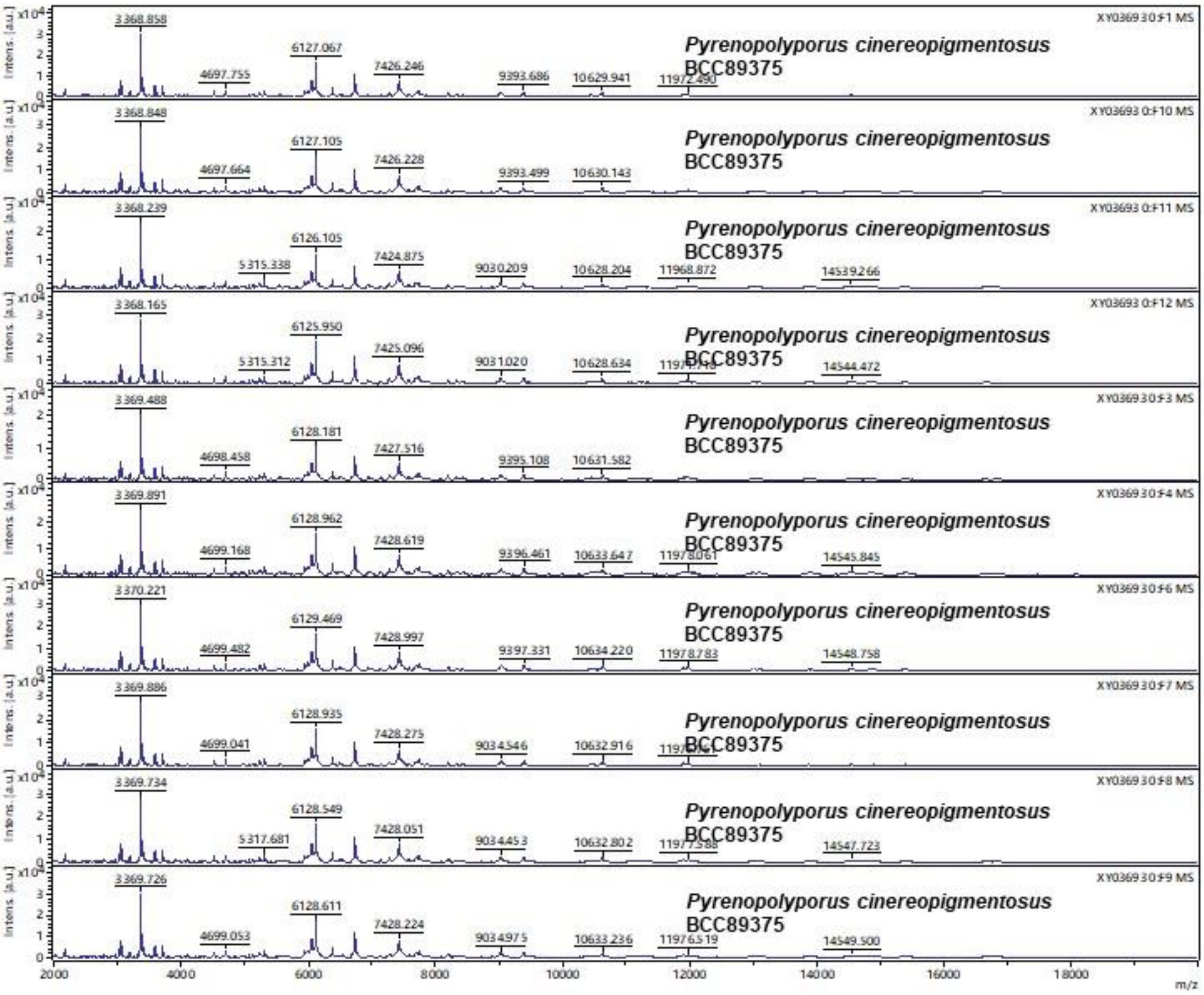

Pyrenopolyporus cinereopigmentosus
BCC89375
Intens. [a.u.]
x10^4
m/z
3368.858
4697.755
6127.067
7426.246
9393.686
10629.941
3368.848
4697.664
6127.105
7426.228
9393.499
10630.143
3368.239
5315.338
6126.105
7424.875
9030.209
10628.204
11968.872
14539.266
3368.165
5315.312
6125.950
7425.096
9031.020
10628.634
14544.472
3369.488
4698.458
6128.181
7427.516
9395.108
10631.582
3369.891
4699.168
6128.962
7428.619
9396.461
10633.647
14545.845
3370.221
4699.482
6129.469
7428.997
9397.331
10634.220
11978.783
14548.758
3369.886
4699.041
6128.935
7428.275
9034.546
10632.916
3369.734
5317.681
6128.549
7428.051
9034.453
10632.802
14547.723
3369.726
4699.053
6128.611
7428.224
9034.975
10633.236
11976.519
14549.500
2000
4000
6000
8000
10000
12000
14000
16000
18000

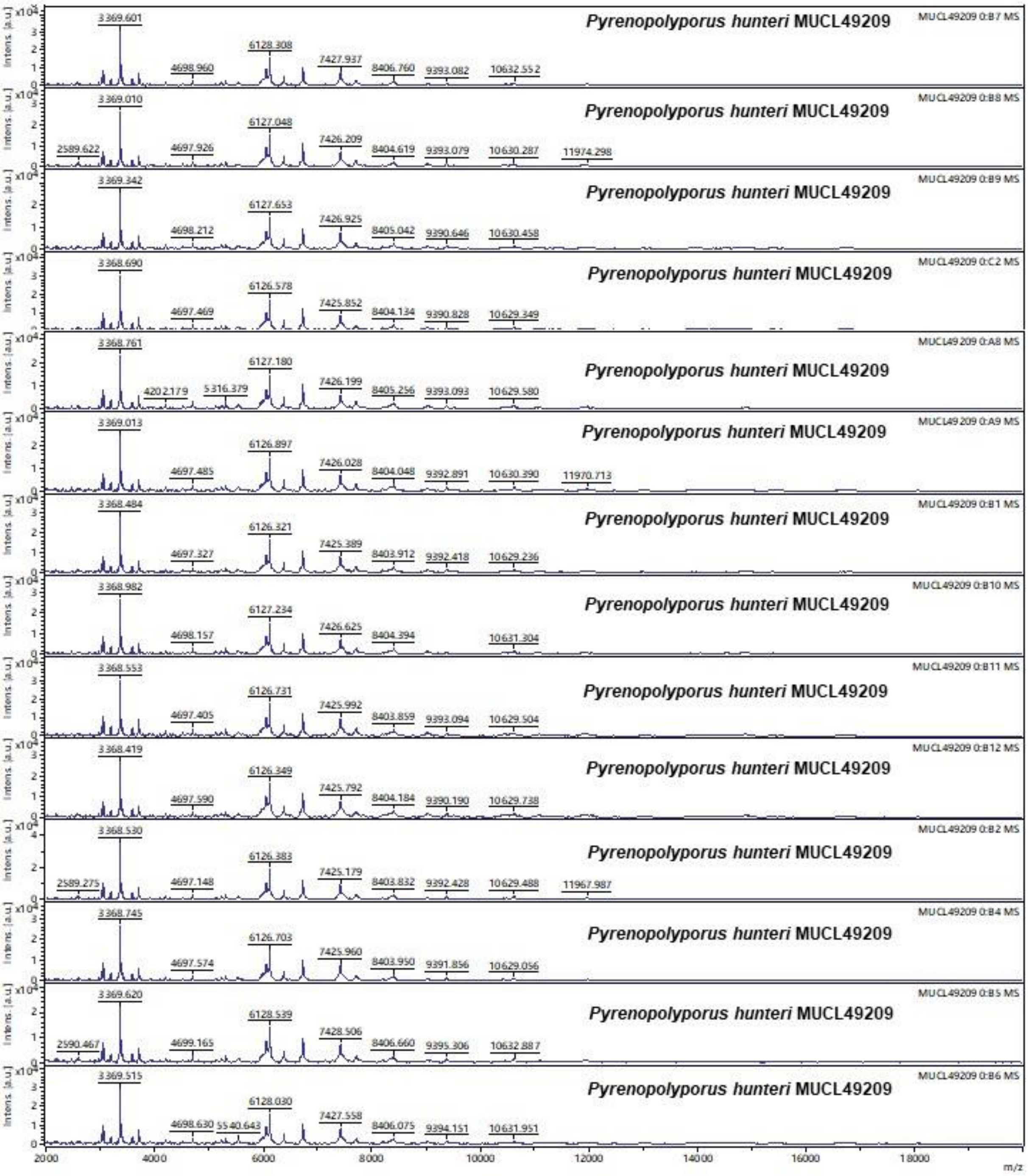
Pyrenopolyporus hunteri MUCL49209
MUCL49209 0:B7 MS
3369.601 4698.960 6128.308 7427.937 8406.760 9393.082 10632.552
MUCL49209 0:B8 MS
3369.010 2589.622 4697.926 6127.048 7426.209 8404.619 9393.079 10630.287 11974.298
MUCL49209 0:B9 MS
3369.342 4698.212 6127.653 7426.925 8405.042 9390.646 10630.458
MUCL49209 0:C2 MS
3368.690 4697.469 6126.578 7425.852 8404.134 9390.828 10629.349
MUCL49209 0:A8 MS
3368.761 4202.179 5316.379 6127.180 7426.199 8405.256 9393.093 10629.580
MUCL49209 0:A9 MS
3369.013 4697.485 6126.897 7426.028 8404.048 9392.891 10630.390 11970.713
MUCL49209 0:B1 MS
3368.484 4697.327 6126.321 7425.389 8403.912 9392.418 10629.236
MUCL49209 0:B10 MS
3368.982 4698.157 6127.234 7426.625 8404.394 10631.304
MUCL49209 0:B11 MS
3368.553 4697.405 6126.731 7425.992 8403.859 9393.094 10629.504
MUCL49209 0:B12 MS
3368.419 4697.590 6126.349 7425.792 8404.184 9390.190 10629.738
MUCL49209 0:B2 MS
3368.530 2589.275 4697.148 6126.383 7425.179 8403.832 9392.428 10629.488 11967.987
MUCL49209 0:B4 MS
3368.745 4697.574 6126.703 7425.960 8403.950 9391.856 10629.056
MUCL49209 0:B5 MS
3369.620 2590.467 4699.165 6128.539 7428.506 8406.660 9395.306 10632.887
MUCL49209 0:B6 MS
3369.515 4698.630 5540.643 6128.030 7427.558 8406.075 9394.151 10631.951
Intens. [a.u.]
x10^4
2000 4000 6000 8000 10000 12000 14000 16000 18000
m/z

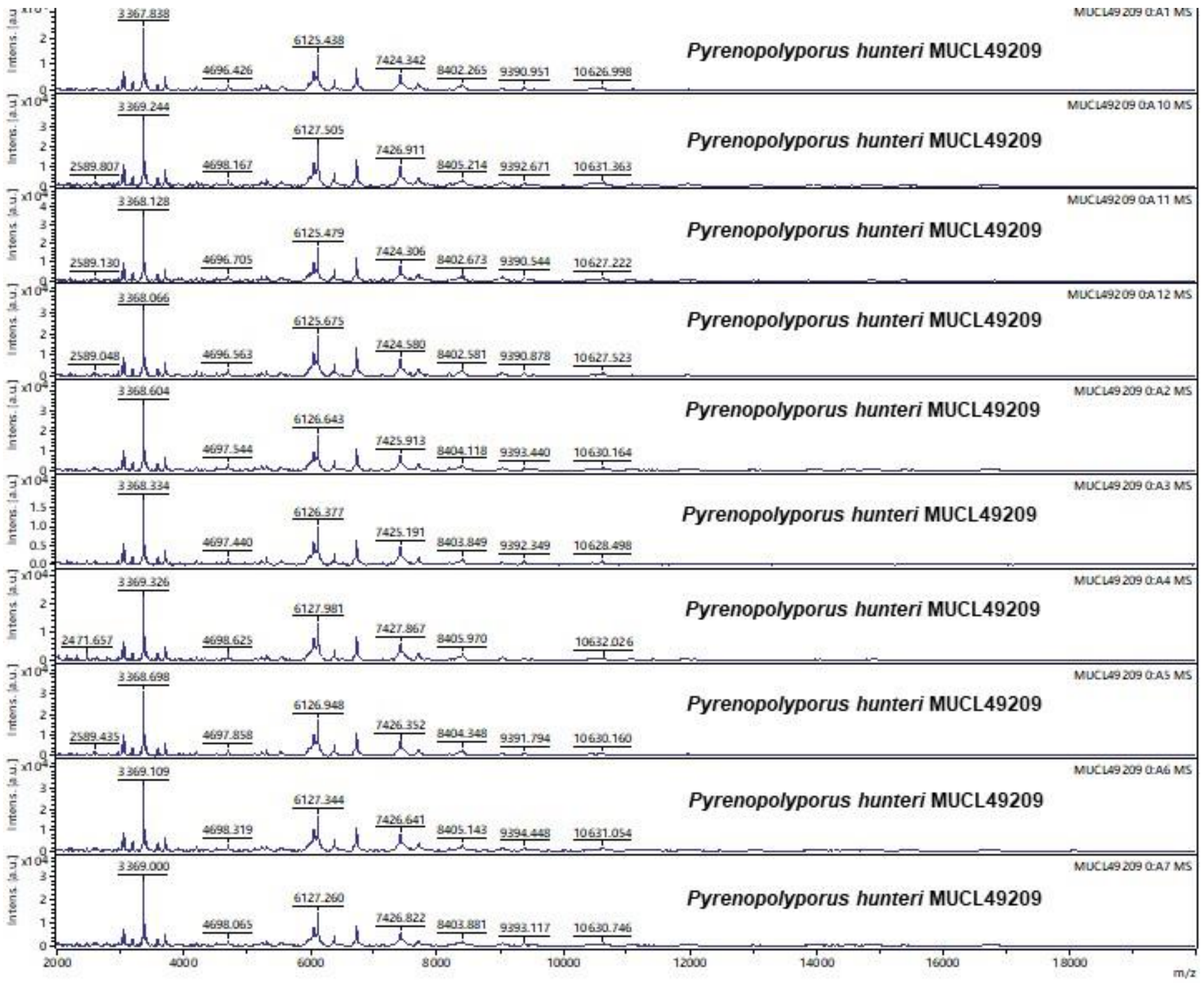

MUCL49209 0:A1 MS
Pyrenopolyporus hunteri MUCL49209
3367.838
6125.438
4696.426
7424.342
8402.265
9390.951
10626.998
MUCL49209 0:A10 MS
Pyrenopolyporus hunteri MUCL49209
3369.244
6127.505
7426.911
2589.807
4698.167
8405.214
9392.671
10631.363
MUCL49209 0:A11 MS
Pyrenopolyporus hunteri MUCL49209
3368.128
6125.479
7424.306
2589.130
4696.705
8402.673
9390.544
10627.222
MUCL49209 0:A12 MS
Pyrenopolyporus hunteri MUCL49209
3368.066
6125.675
7424.580
2589.048
4696.563
8402.581
9390.878
10627.523
MUCL49209 0:A2 MS
Pyrenopolyporus hunteri MUCL49209
3368.604
6126.643
7425.913
4697.544
8404.118
9393.440
10630.164
MUCL49209 0:A3 MS
Pyrenopolyporus hunteri MUCL49209
3368.334
6126.377
7425.191
4697.440
8403.849
9392.349
10628.498
MUCL49209 0:A4 MS
Pyrenopolyporus hunteri MUCL49209
3369.326
6127.981
7427.867
2471.657
4698.625
8405.970
10632.026
MUCL49209 0:A5 MS
Pyrenopolyporus hunteri MUCL49209
3368.698
6126.948
7426.352
2589.435
4697.858
8404.348
9391.794
10630.160
MUCL49209 0:A6 MS
Pyrenopolyporus hunteri MUCL49209
3369.109
6127.344
7426.641
4698.319
8405.143
9394.448
10631.054
MUCL49209 0:A7 MS
Pyrenopolyporus hunteri MUCL49209
3369.000
6127.260
7426.822
4698.065
8403.881
9393.117
10630.746
Intens. [a.u.]
x10^4
2000
4000
6000
8000
10000
12000
14000
16000
18000
m/z

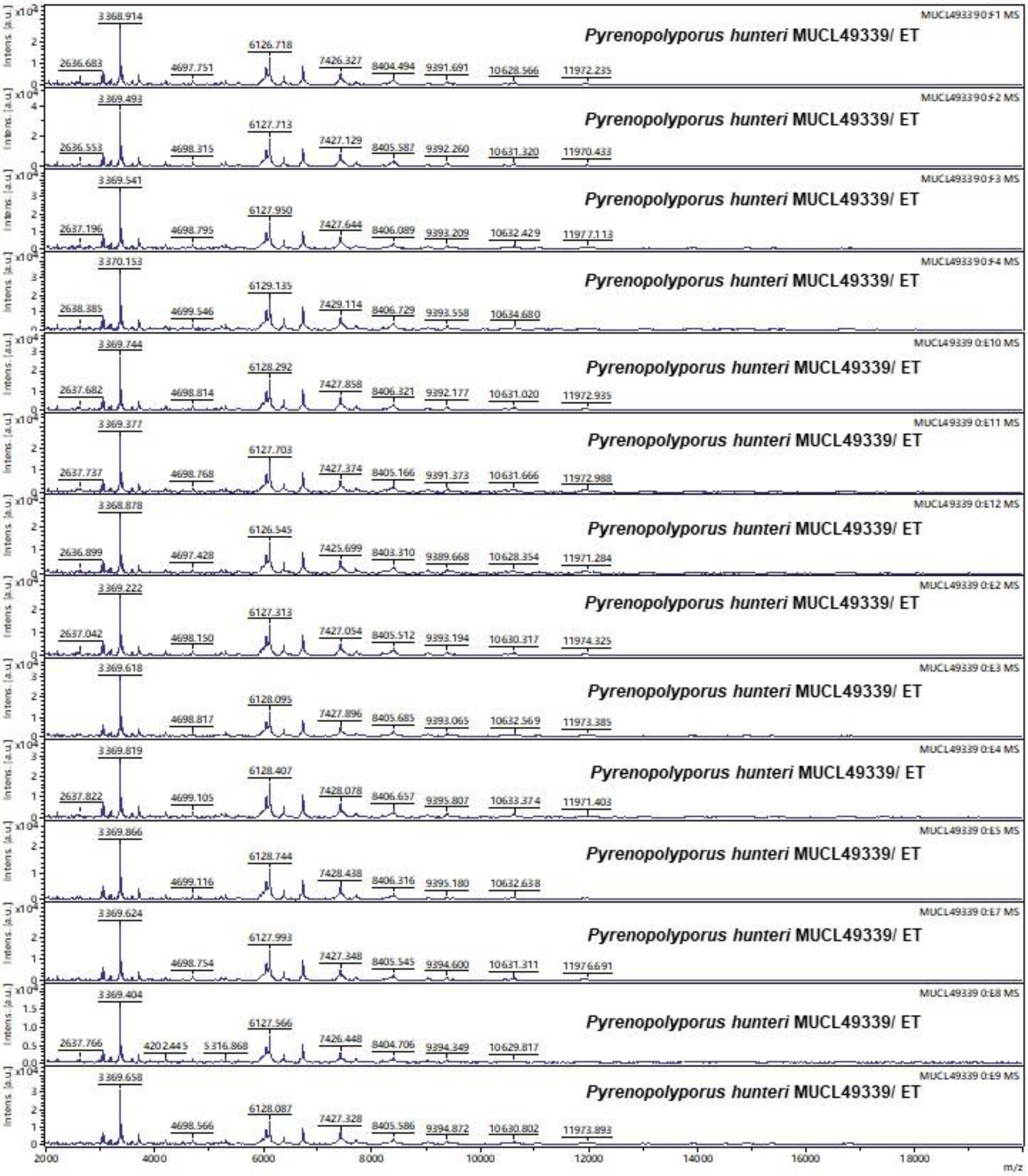
Pyrenopolyporus hunteri MUCL49339/ ET
Intens. [a.u.]
m/z

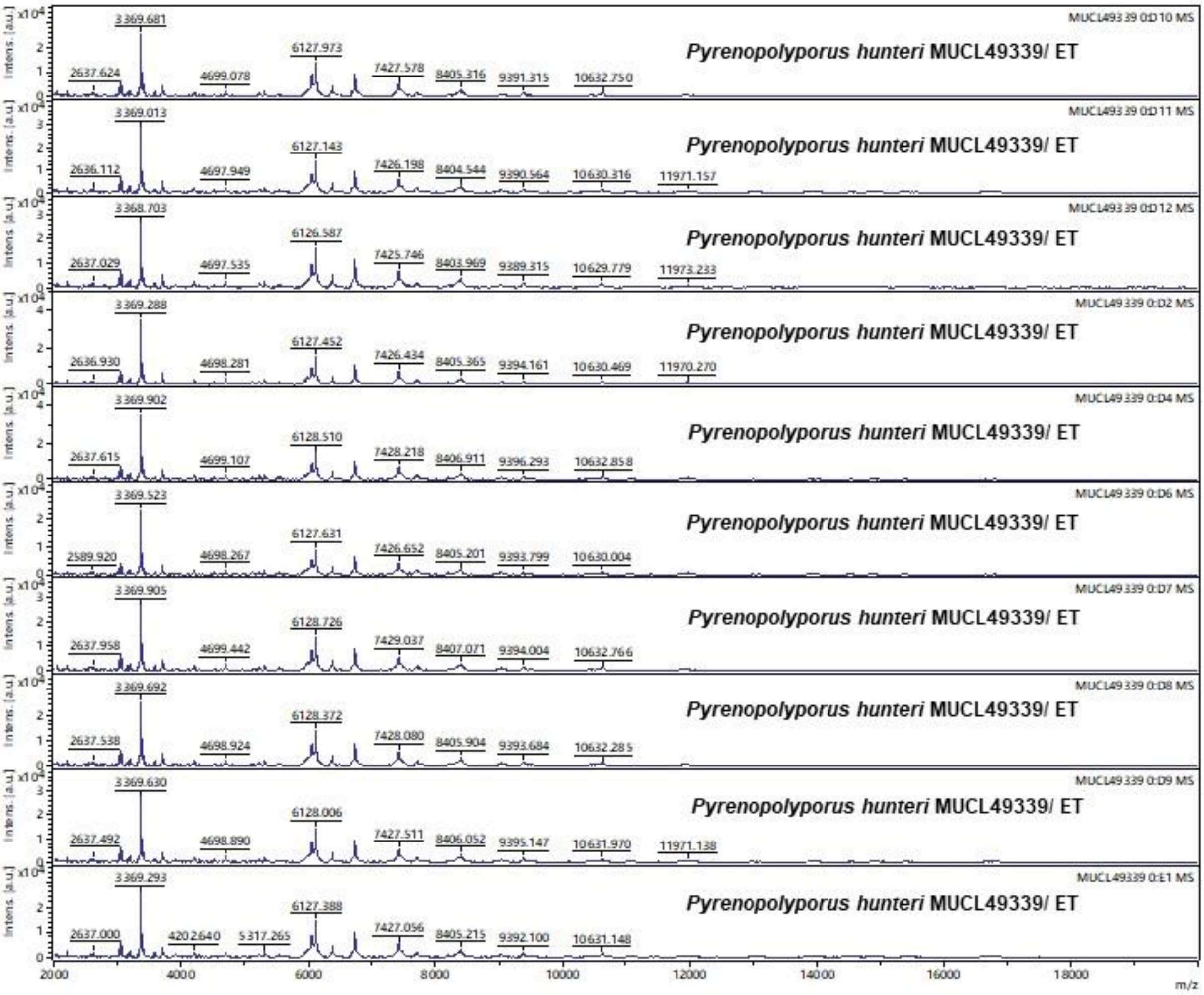
Pyrenopolyporus hunteri MUCL49339/ ET
MUCL49339 0:D10 MS
3369.681
2637.624
4699.078
6127.973
7427.578
8405.316
9391.315
10632.750
MUCL49339 0:D11 MS
3369.013
2636.112
4697.949
6127.143
7426.198
8404.544
9390.564
10630.316
11971.157
MUCL49339 0:D12 MS
3368.703
2637.029
4697.535
6126.587
7425.746
8403.969
9389.315
10629.779
11973.233
MUCL49339 0:D2 MS
3369.288
2636.930
4698.281
6127.452
7426.434
8405.365
9394.161
10630.469
11970.270
MUCL49339 0:D4 MS
3369.902
2637.615
4699.107
6128.510
7428.218
8406.911
9396.293
10632.858
MUCL49339 0:D6 MS
3369.523
2589.920
4698.267
6127.631
7426.652
8405.201
9393.799
10630.004
MUCL49339 0:D7 MS
3369.905
2637.958
4699.442
6128.726
7429.037
8407.071
9394.004
10632.766
MUCL49339 0:D8 MS
3369.692
2637.538
4698.924
6128.372
7428.080
8405.904
9393.684
10632.285
MUCL49339 0:D9 MS
3369.630
2637.492
4698.890
6128.006
7427.511
8406.052
9395.147
10631.970
11971.138
MUCL49339 0:E1 MS
3369.293
2637.000
4202.640
5317.265
6127.388
7427.056
8405.215
9392.100
10631.148
Intens. [a.u.]
x10^4
2000
4000
6000
8000
10000
12000
14000
16000
18000
m/z

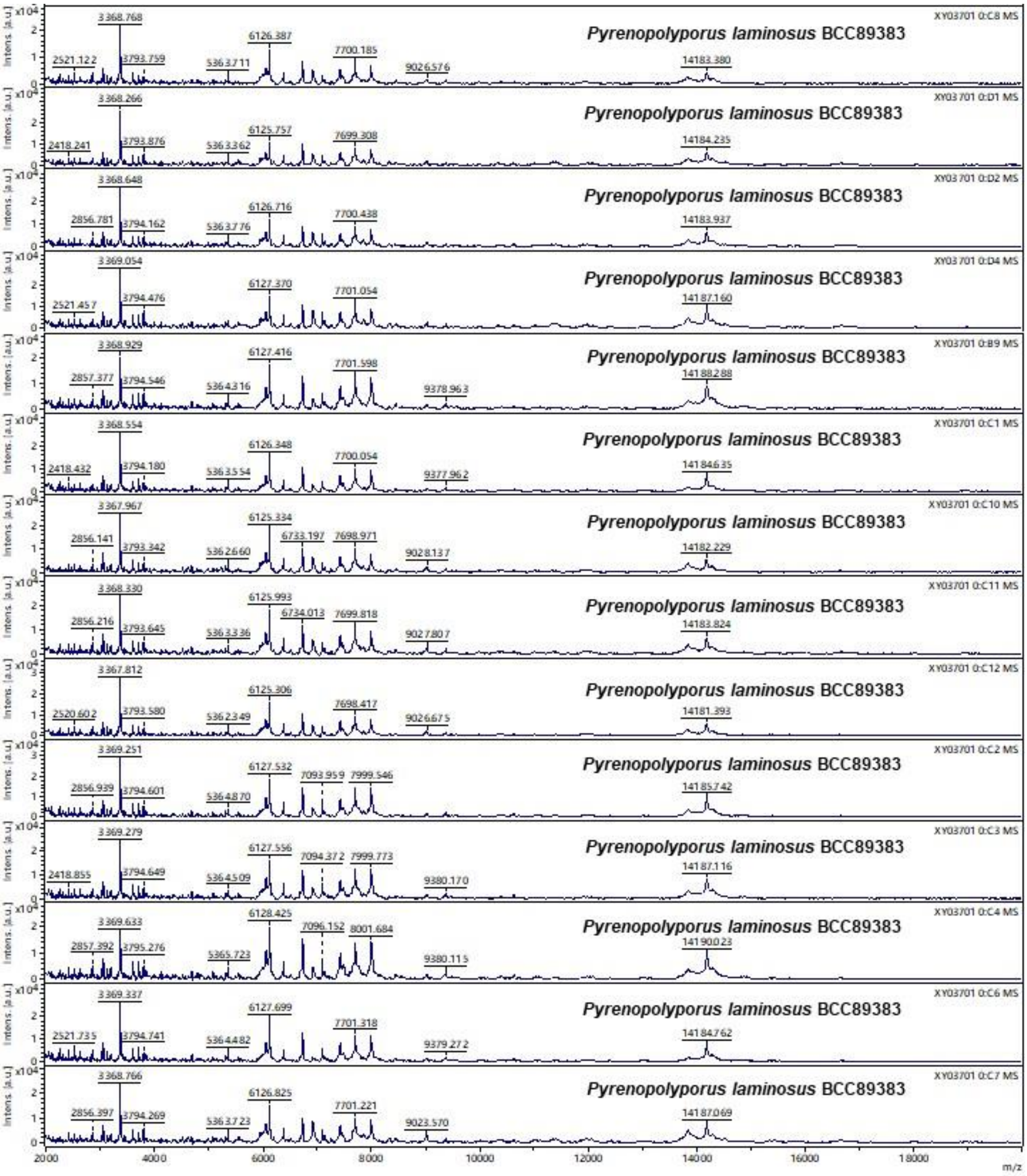
Pyrenopolyporus laminosus BCC89383
XY03701 0:C8 MS
3368.768
6126.387
7700.185
2521.122
3793.759
5363.711
9026.576
14183.380
XY03701 0:D1 MS
3368.266
6125.757
7699.308
2418.241
3793.876
5363.362
14184.235
XY03701 0:D2 MS
3368.648
6126.716
7700.438
2856.781
3794.162
5363.776
14183.937
XY03701 0:D4 MS
3369.054
6127.370
7701.054
2521.457
3794.476
14187.160
XY03701 0:B9 MS
3368.929
6127.416
7701.598
2857.377
3794.546
5364.316
9378.963
14188.288
XY03701 0:C1 MS
3368.554
6126.348
7700.054
2418.432
3794.180
5363.554
9377.962
14184.635
XY03701 0:C10 MS
3367.967
6125.334
6733.197
7698.971
2856.141
3793.342
5362.660
9028.137
14182.229
XY03701 0:C11 MS
3368.330
6125.993
6734.013
7699.818
2856.216
3793.645
5363.336
9027.807
14183.824
XY03701 0:C12 MS
3367.812
6125.306
7698.417
2520.602
3793.580
5362.349
9026.675
14181.393
XY03701 0:C2 MS
3369.251
6127.532
7093.959
7999.546
2856.939
3794.601
5364.870
14185.742
XY03701 0:C3 MS
3369.279
6127.556
7094.372
7999.773
2418.855
3794.649
5364.509
9380.170
14187.116
XY03701 0:C4 MS
6128.425
3369.633
7096.152
8001.684
2857.392
3795.276
5365.723
9380.115
14190.023
XY03701 0:C6 MS
3369.337
6127.699
7701.318
2521.735
3794.741
5364.482
9379.272
14184.762
XY03701 0:C7 MS
3368.766
6126.825
7701.221
2856.397
3794.269
5363.723
9023.570
14187.069
Intens. [a.u.]
x10^4
2000
4000
6000
8000
10000
12000
14000
16000
18000
m/z

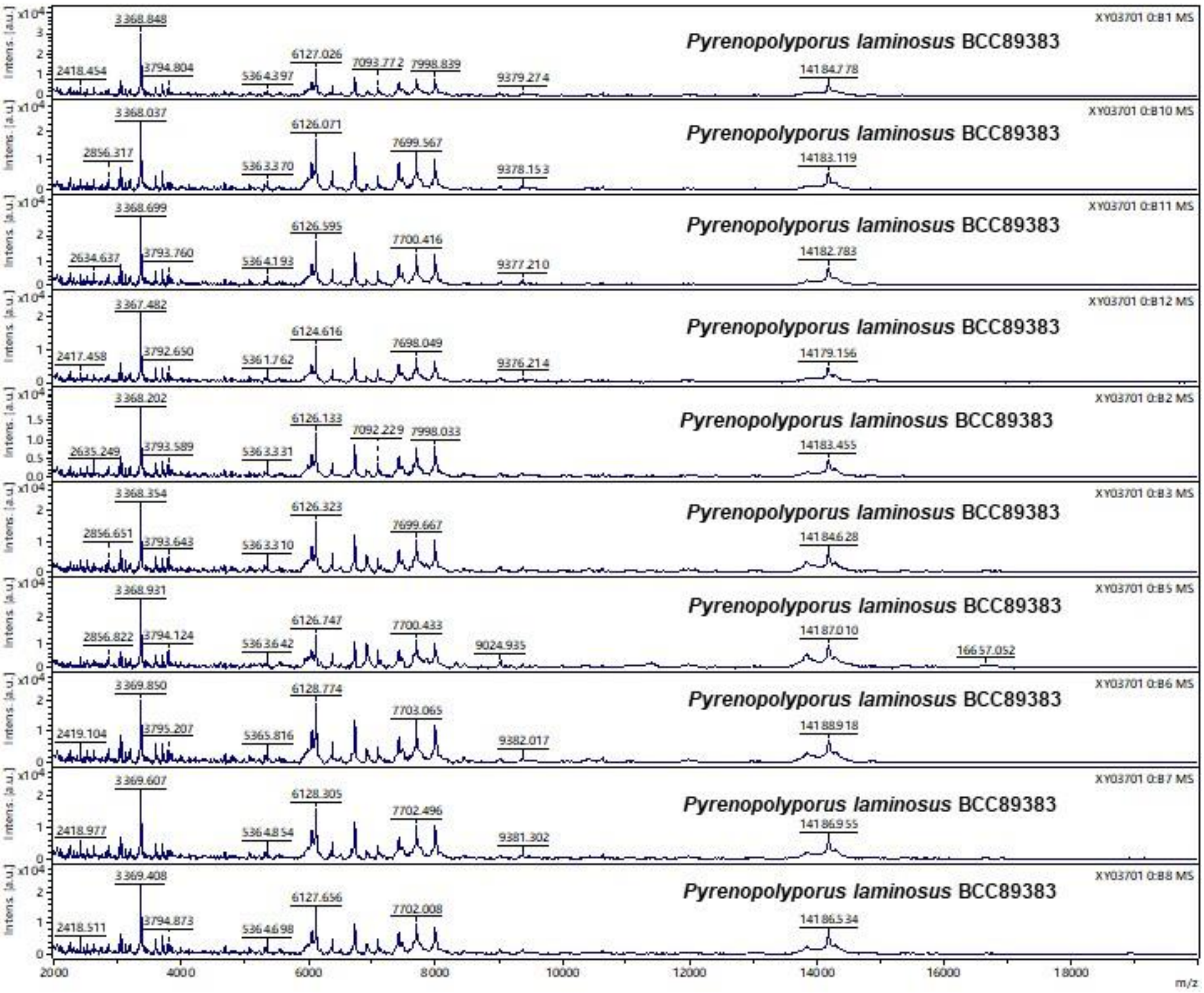
Pyrenopolyporus laminosus BCC89383
XY03701 0:B1 MS
XY03701 0:B10 MS
XY03701 0:B11 MS
XY03701 0:B12 MS
XY03701 0:B2 MS
XY03701 0:B3 MS
XY03701 0:B5 MS
XY03701 0:B6 MS
XY03701 0:B7 MS
XY03701 0:B8 MS
Intens. [a.u.]
x10^4
m/z
2000
4000
6000
8000
10000
12000
14000
16000
18000

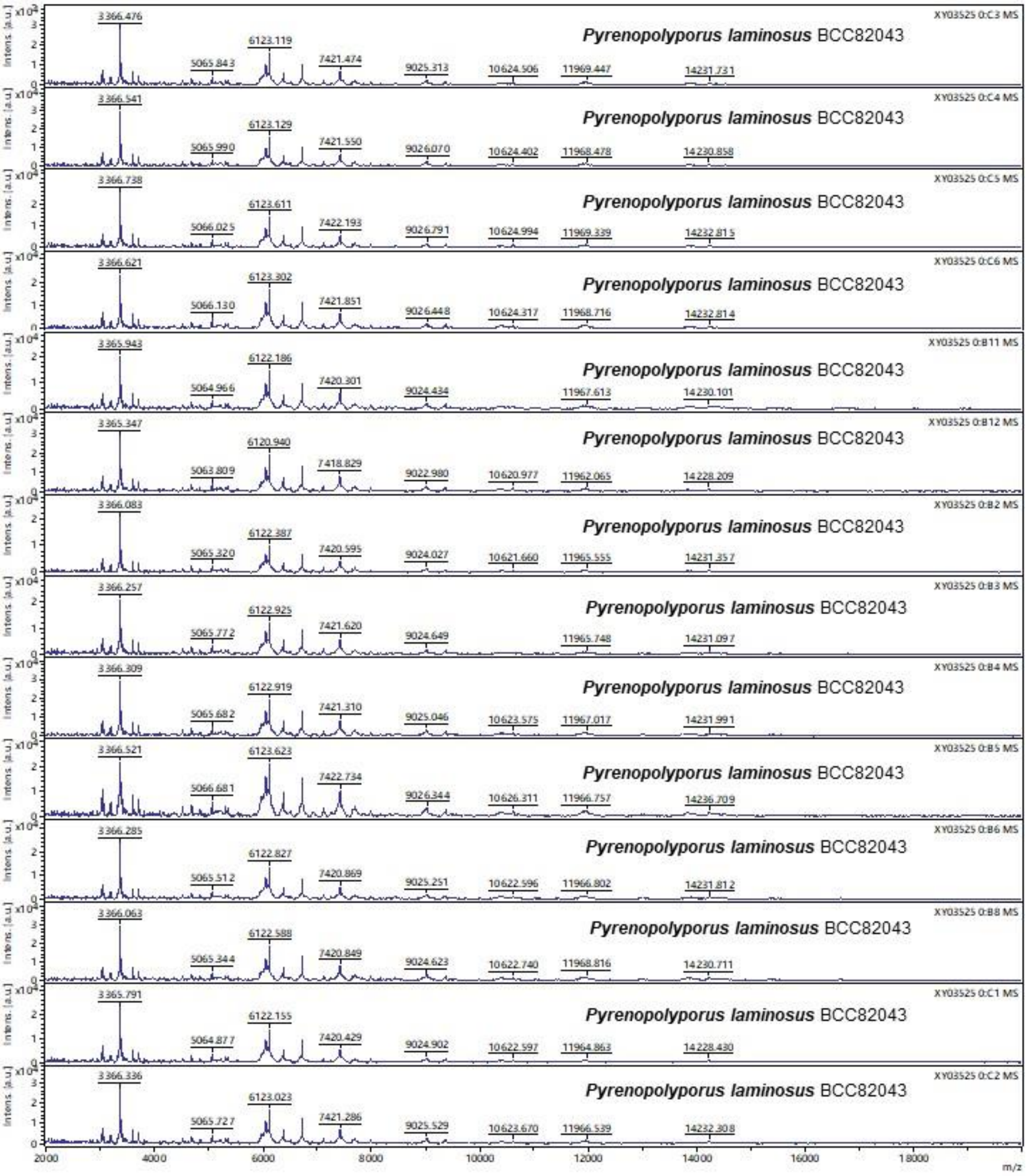
Intens. [a.u.]
x10^4
Pyrenopolyporus laminosus BCC82043
XY03525 0:C3 MS
3366.476
5065.843
6123.119
7421.474
9025.313
10624.506
11969.447
14231.731
XY03525 0:C4 MS
3366.541
5065.990
6123.129
7421.550
9026.070
10624.402
11968.478
14230.858
XY03525 0:C5 MS
3366.738
5066.025
6123.611
7422.193
9026.791
10624.994
11969.339
14232.815
XY03525 0:C6 MS
3366.621
5066.130
6123.302
7421.851
9026.448
10624.317
11968.716
14232.814
XY03525 0:B11 MS
3365.943
5064.966
6122.186
7420.301
9024.434
11967.613
14230.101
XY03525 0:B12 MS
3365.347
5063.809
6120.940
7418.829
9022.980
10620.977
11962.065
14228.209
XY03525 0:B2 MS
3366.083
5065.320
6122.387
7420.595
9024.027
10621.660
11965.555
14231.357
XY03525 0:B3 MS
3366.257
5065.772
6122.925
7421.620
9024.649
11965.748
14231.097
XY03525 0:B4 MS
3366.309
5065.682
6122.919
7421.310
9025.046
10623.575
11967.017
14231.991
XY03525 0:B5 MS
3366.521
5066.681
6123.623
7422.734
9026.344
10626.311
11966.757
14236.709
XY03525 0:B6 MS
3366.285
5065.512
6122.827
7420.869
9025.251
10622.596
11966.802
14231.812
XY03525 0:B8 MS
3366.063
5065.344
6122.588
7420.849
9024.623
10622.740
11968.816
14230.711
XY03525 0:C1 MS
3365.791
5064.877
6122.155
7420.429
9024.902
10622.597
11964.863
14228.430
XY03525 0:C2 MS
3366.336
5065.727
6123.023
7421.286
9025.529
10623.670
11966.539
14232.308
2000
4000
6000
8000
10000
12000
14000
16000
18000
m/z

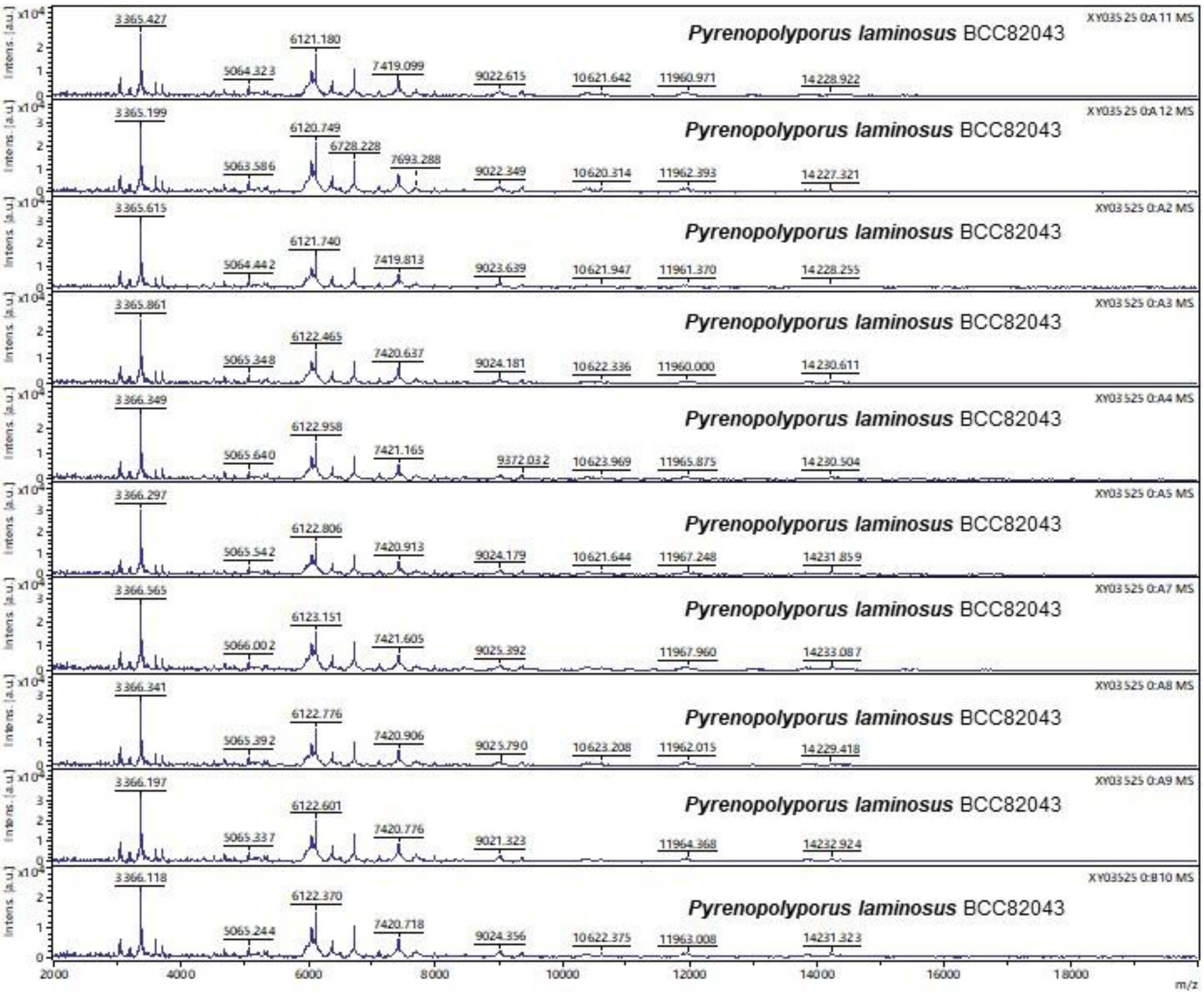

Pyrenopolyporus laminosus BCC82043
XY03525 0:A11 MS
3365.427
6121.180
5064.323
7419.099
9022.615
10621.642
11960.971
14228.922
XY03525 0:A12 MS
3365.199
6120.749
6728.228
7693.288
5063.586
9022.349
10620.314
11962.393
14227.321
XY03525 0:A2 MS
3365.615
6121.740
5064.442
7419.813
9023.639
10621.947
11961.370
14228.255
XY03525 0:A3 MS
3365.861
6122.465
7420.637
5065.348
9024.181
10622.336
11960.000
14230.611
XY03525 0:A4 MS
3366.349
6122.958
5065.640
7421.165
9372.032
10623.969
11965.875
14230.504
XY03525 0:A5 MS
3366.297
6122.806
7420.913
5065.542
9024.179
10621.644
11967.248
14231.859
XY03525 0:A7 MS
3366.565
6123.151
7421.605
5066.002
9025.392
11967.960
14233.087
XY03525 0:A8 MS
3366.341
6122.776
7420.906
5065.392
9025.790
10623.208
11962.015
14229.418
XY03525 0:A9 MS
3366.197
6122.601
7420.776
5065.337
9021.323
11964.368
14232.924
XY03525 0:B10 MS
3366.118
6122.370
7420.718
5065.244
9024.356
10622.375
11963.008
14231.323
Intens. [a.u.]
x10^4
m/z
2000
4000
6000
8000
10000
12000
14000
16000
18000

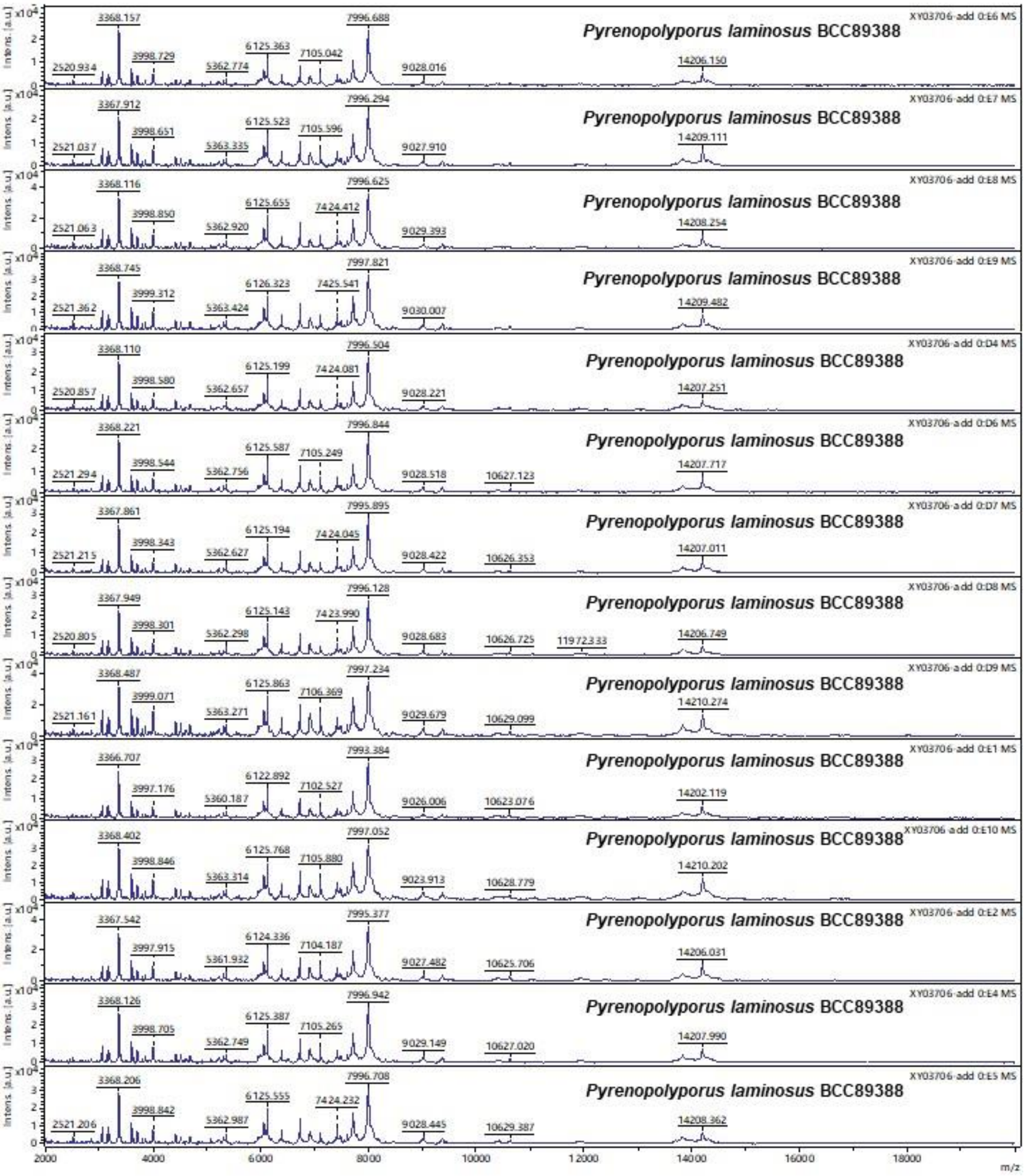

Intens. [a.u.]
x10^4
Pyrenopolyporus laminosus BCC89388
XY03706-add 0:E6 MS
3368.157 3998.729 2520.934 5362.774 6125.363 7105.042 7996.688 9028.016 14206.150
XY03706-add 0:E7 MS
3367.912 3998.651 2521.037 5363.335 6125.523 7105.596 7996.294 9027.910 14209.111
XY03706-add 0:E8 MS
3368.116 3998.850 2521.063 5362.920 6125.655 7424.412 7996.625 9029.393 14208.254
XY03706-add 0:E9 MS
3368.745 3999.312 2521.362 5363.424 6126.323 7425.541 7997.821 9030.007 14209.482
XY03706-add 0:D4 MS
3368.110 3998.580 2520.857 5362.657 6125.199 7424.081 7996.504 9028.221 14207.251
XY03706-add 0:D6 MS
3368.221 3998.544 2521.294 5362.756 6125.587 7105.249 7996.844 9028.518 10627.123 14207.717
XY03706-add 0:D7 MS
3367.861 3998.343 2521.215 5362.627 6125.194 7424.045 7995.895 9028.422 10626.353 14207.011
XY03706-add 0:D8 MS
3367.949 3998.301 2520.805 5362.298 6125.143 7423.990 7996.128 9028.683 10626.725 11972.333 14206.749
XY03706-add 0:D9 MS
3368.487 3999.071 2521.161 5363.271 6125.863 7106.369 7997.234 9029.679 10629.099 14210.274
XY03706-add 0:E1 MS
3366.707 3997.176 5360.187 6122.892 7102.527 7993.384 9026.006 10623.076 14202.119
XY03706-add 0:E10 MS
3368.402 3998.846 5363.314 6125.768 7105.880 7997.052 9023.913 10628.779 14210.202
XY03706-add 0:E2 MS
3367.542 3997.915 5361.932 6124.336 7104.187 7995.377 9027.482 10625.706 14206.031
XY03706-add 0:E4 MS
3368.126 3998.705 5362.749 6125.387 7105.265 7996.942 9029.149 10627.020 14207.990
XY03706-add 0:E5 MS
3368.206 3998.842 2521.206 5362.987 6125.555 7424.232 7996.708 9028.445 10629.387 14208.362
2000 4000 6000 8000 10000 12000 14000 16000 18000
m/z

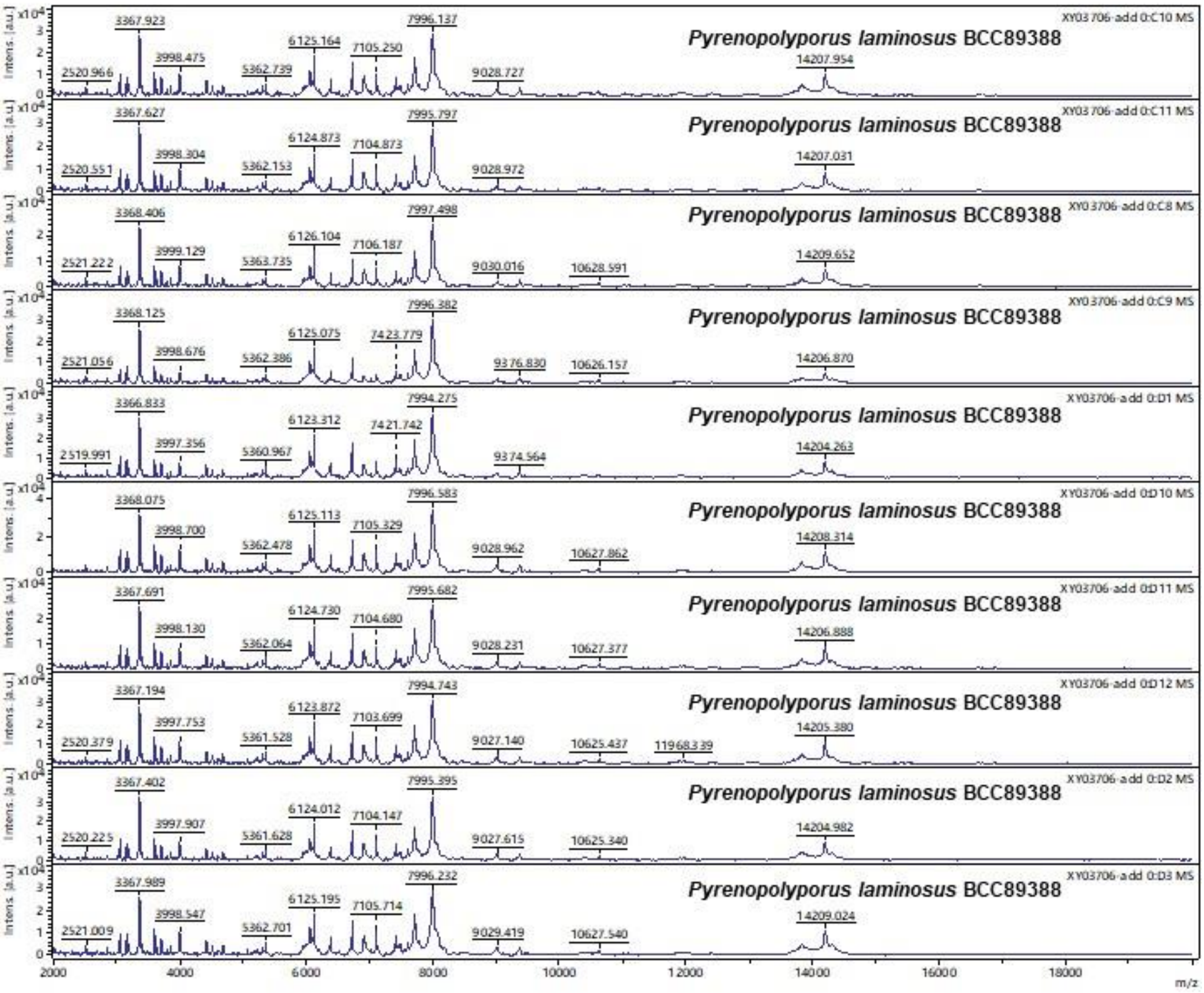
Pyrenopolyporus laminosus BCC89388
XY03706-add 0:C10 MS
3367.923
2520.966
3998.475
5362.739
6125.164
7105.250
7996.137
9028.727
14207.954
XY03706-add 0:C11 MS
3367.627
2520.551
3998.304
5362.153
6124.873
7104.873
7995.797
9028.972
14207.031
XY03706-add 0:C8 MS
3368.406
2521.222
3999.129
5363.735
6126.104
7106.187
7997.498
9030.016
10628.591
14209.652
XY03706-add 0:C9 MS
3368.125
2521.056
3998.676
5362.386
6125.075
7423.779
7996.382
9376.830
10626.157
14206.870
XY03706-add 0:D1 MS
3366.833
2519.991
3997.356
5360.967
6123.312
7421.742
7994.275
9374.564
14204.263
XY03706-add 0:D10 MS
3368.075
3998.700
5362.478
6125.113
7105.329
7996.583
9028.962
10627.862
14208.314
XY03706-add 0:D11 MS
3367.691
3998.130
5362.064
6124.730
7104.680
7995.682
9028.231
10627.377
14206.888
XY03706-add 0:D12 MS
3367.194
2520.379
3997.753
5361.528
6123.872
7103.699
7994.743
9027.140
10625.437
11968.339
14205.380
XY03706-add 0:D2 MS
3367.402
2520.225
3997.907
5361.628
6124.012
7104.147
7995.395
9027.615
10625.340
14204.982
XY03706-add 0:D3 MS
3367.989
2521.009
3998.547
5362.701
6125.195
7105.714
7996.232
9029.419
10627.540
14209.024
Intens. [a.u.]
x10^4
2000
4000
6000
8000
10000
12000
14000
16000
18000
m/z

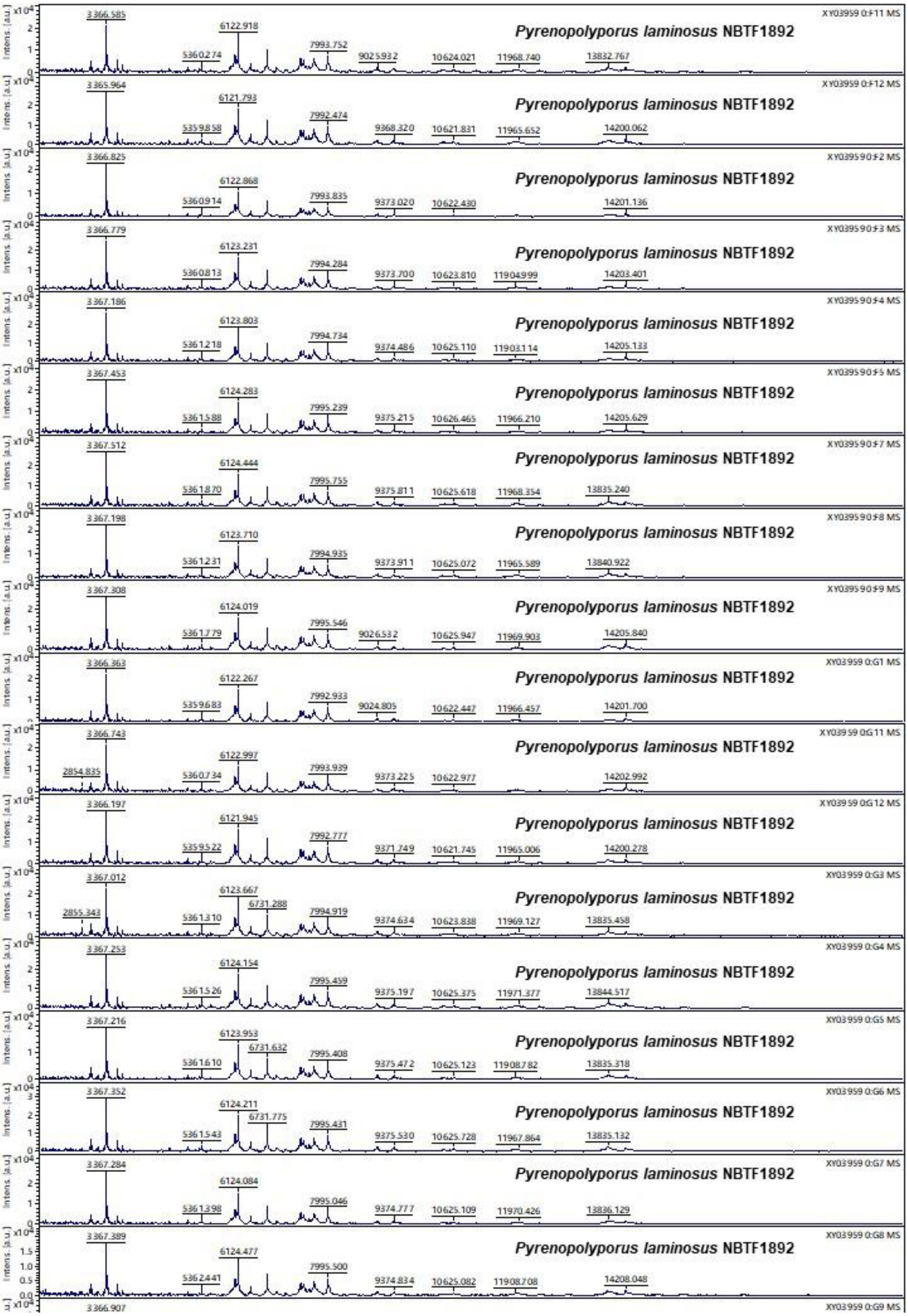
XY03959 0:F11 MS
Pyrenopolyporus laminosus NBTF1892
XY03959 0:F12 MS
Pyrenopolyporus laminosus NBTF1892
XY0395900:F2 MS
Pyrenopolyporus laminosus NBTF1892
XY0395900:F3 MS
Pyrenopolyporus laminosus NBTF1892
XY0395900:F4 MS
Pyrenopolyporus laminosus NBTF1892
XY0395900:F5 MS
Pyrenopolyporus laminosus NBTF1892
XY0395900:F7 MS
Pyrenopolyporus laminosus NBTF1892
XY0395900:F8 MS
Pyrenopolyporus laminosus NBTF1892
XY0395900:F9 MS
Pyrenopolyporus laminosus NBTF1892
XY03959 0:G1 MS
Pyrenopolyporus laminosus NBTF1892
XY03959 0:G11 MS
Pyrenopolyporus laminosus NBTF1892
XY03959 0:G12 MS
Pyrenopolyporus laminosus NBTF1892
XY03959 0:G3 MS
Pyrenopolyporus laminosus NBTF1892
XY03959 0:G4 MS
Pyrenopolyporus laminosus NBTF1892
XY03959 0:G5 MS
Pyrenopolyporus laminosus NBTF1892
XY03959 0:G6 MS
Pyrenopolyporus laminosus NBTF1892
XY03959 0:G7 MS
Pyrenopolyporus laminosus NBTF1892
XY03959 0:G8 MS
Pyrenopolyporus laminosus NBTF1892
XY03959 0:G9 MS

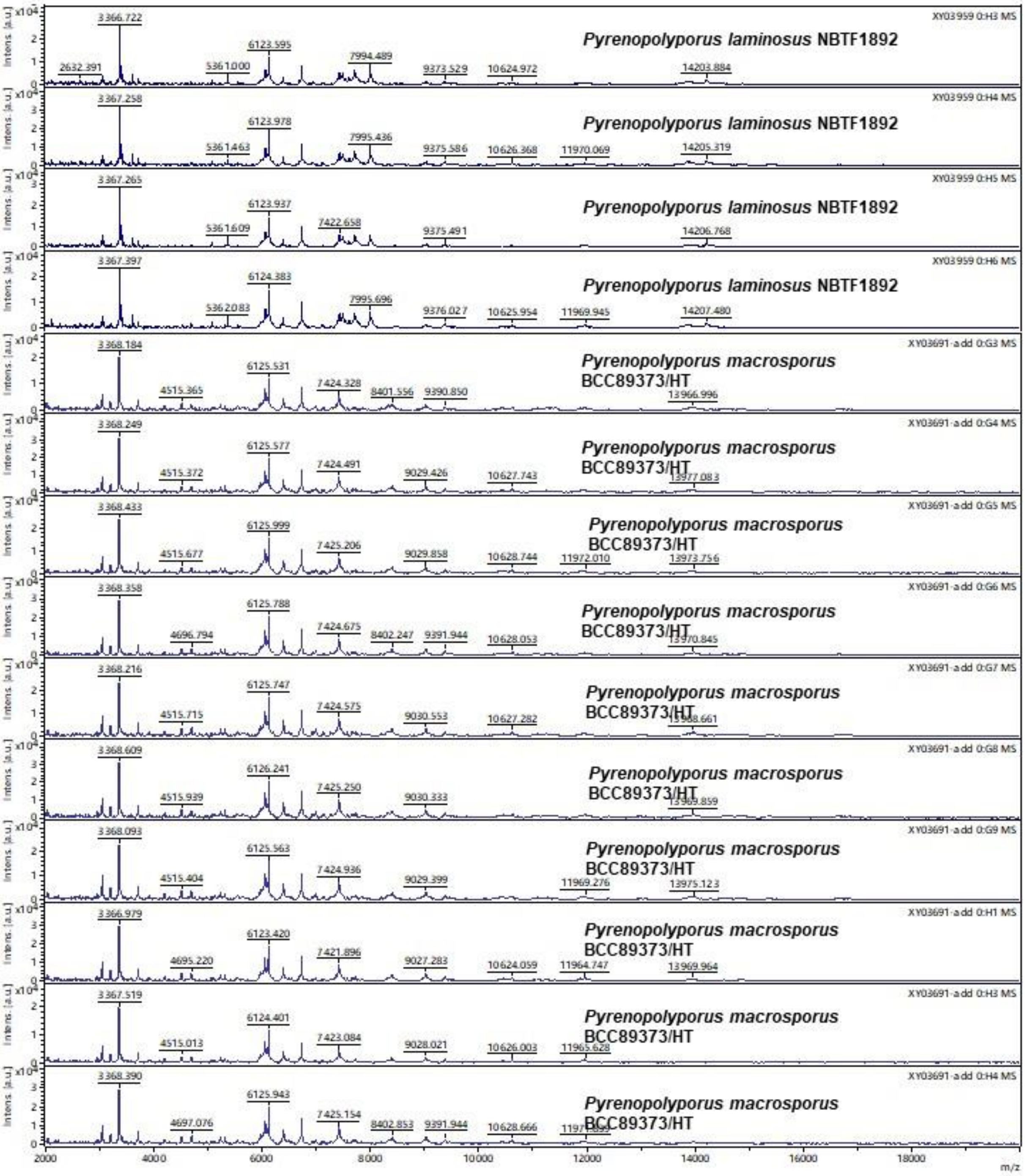
XY03959 0:H3 MS
Pyrenopolyporus laminosus NBTF1892
XY03959 0:H4 MS
Pyrenopolyporus laminosus NBTF1892
XY03959 0:H5 MS
Pyrenopolyporus laminosus NBTF1892
XY03959 0:H6 MS
Pyrenopolyporus laminosus NBTF1892
XY03691-add 0:G3 MS
Pyrenopolyporus macrosporus BCC89373/HT
XY03691-add 0:G4 MS
Pyrenopolyporus macrosporus BCC89373/HT
XY03691-add 0:G5 MS
Pyrenopolyporus macrosporus BCC89373/HT
XY03691-add 0:G6 MS
Pyrenopolyporus macrosporus BCC89373/HT
XY03691-add 0:G7 MS
Pyrenopolyporus macrosporus BCC89373/HT
XY03691-add 0:G8 MS
Pyrenopolyporus macrosporus BCC89373/HT
XY03691-add 0:G9 MS
Pyrenopolyporus macrosporus BCC89373/HT
XY03691-add 0:H1 MS
Pyrenopolyporus macrosporus BCC89373/HT
XY03691-add 0:H3 MS
Pyrenopolyporus macrosporus BCC89373/HT
XY03691-add 0:H4 MS
Pyrenopolyporus macrosporus BCC89373/HT
Intens. [a.u.]
m/z

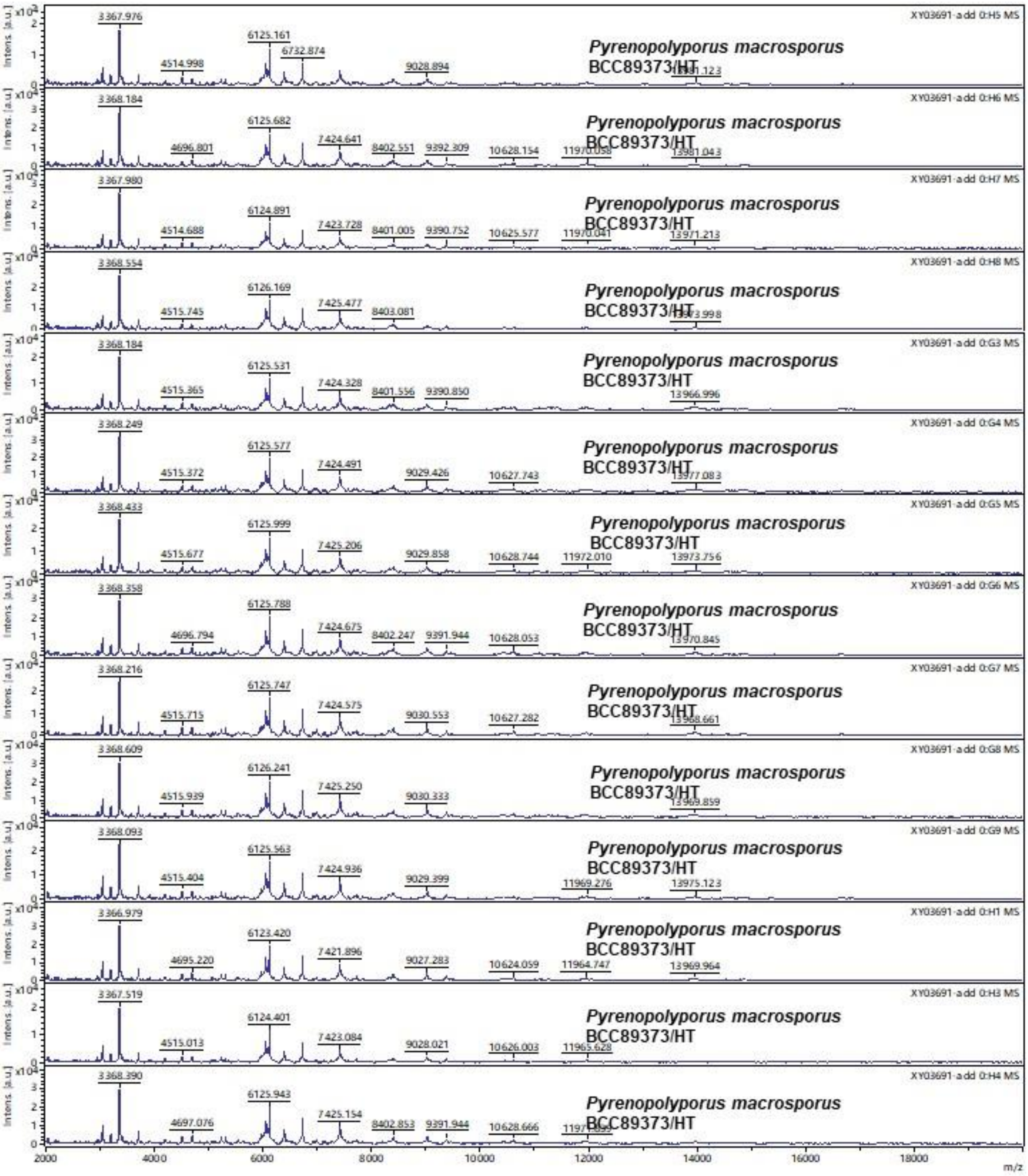
XY03691-add 0:H5 MS
XY03691-add 0:H6 MS
XY03691-add 0:H7 MS
XY03691-add 0:H8 MS
XY03691-add 0:G3 MS
XY03691-add 0:G4 MS
XY03691-add 0:G5 MS
XY03691-add 0:G6 MS
XY03691-add 0:G7 MS
XY03691-add 0:G8 MS
XY03691-add 0:G9 MS
XY03691-add 0:H1 MS
XY03691-add 0:H3 MS
XY03691-add 0:H4 MS
Pyrenopolyporus macrosporus
BCC89373/HT
Intens. [a.u.]
m/z

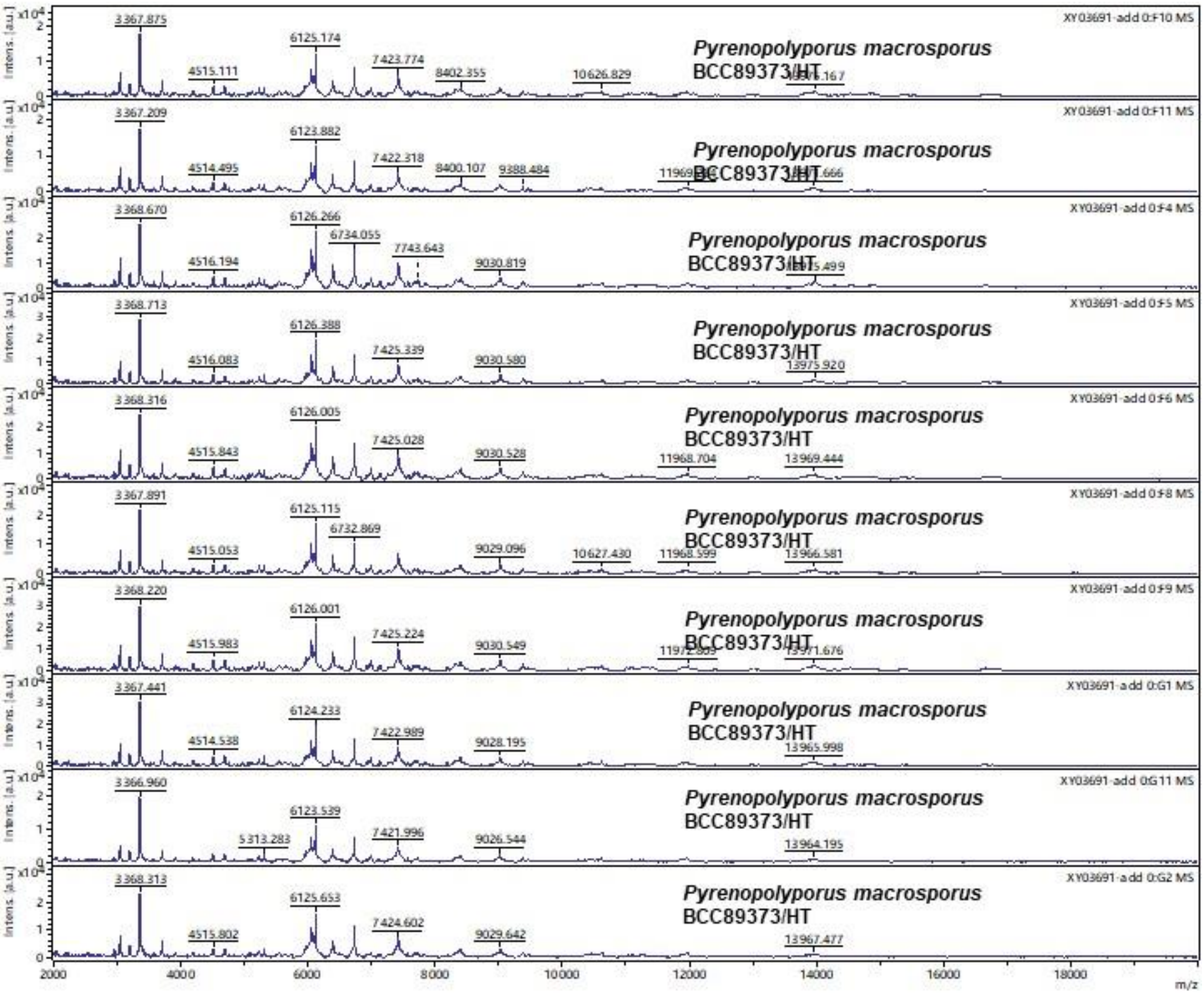
XY03691-add 0:F10 MS
Pyrenopolyporus macrosporus
BCC89373/HT
3367.875
4515.111
6125.174
7423.774
8402.355
10626.829
XY03691-add 0:F11 MS
3367.209
4514.495
6123.882
7422.318
8400.107
9388.484
XY03691-add 0:F4 MS
3368.670
4516.194
6126.266
6734.055
7743.643
9030.819
XY03691-add 0:F5 MS
3368.713
4516.083
6126.388
7425.339
9030.580
13975.920
XY03691-add 0:F6 MS
3368.316
4515.843
6126.005
7425.028
9030.528
11968.704
13969.444
XY03691-add 0:F8 MS
3367.891
4515.053
6125.115
6732.869
9029.096
10627.430
11968.599
13966.581
XY03691-add 0:F9 MS
3368.220
4515.983
6126.001
7425.224
9030.549
XY03691-add 0:G1 MS
3367.441
4514.538
6124.233
7422.989
9028.195
13965.998
XY03691-add 0:G11 MS
3366.960
5313.283
6123.539
7421.996
9026.544
13964.195
XY03691-add 0:G2 MS
3368.313
4515.802
6125.653
7424.602
9029.642
13967.477
Intens. [a.u.]
x10⁴
m/z
2000
4000
6000
8000
10000
12000
14000
16000
18000

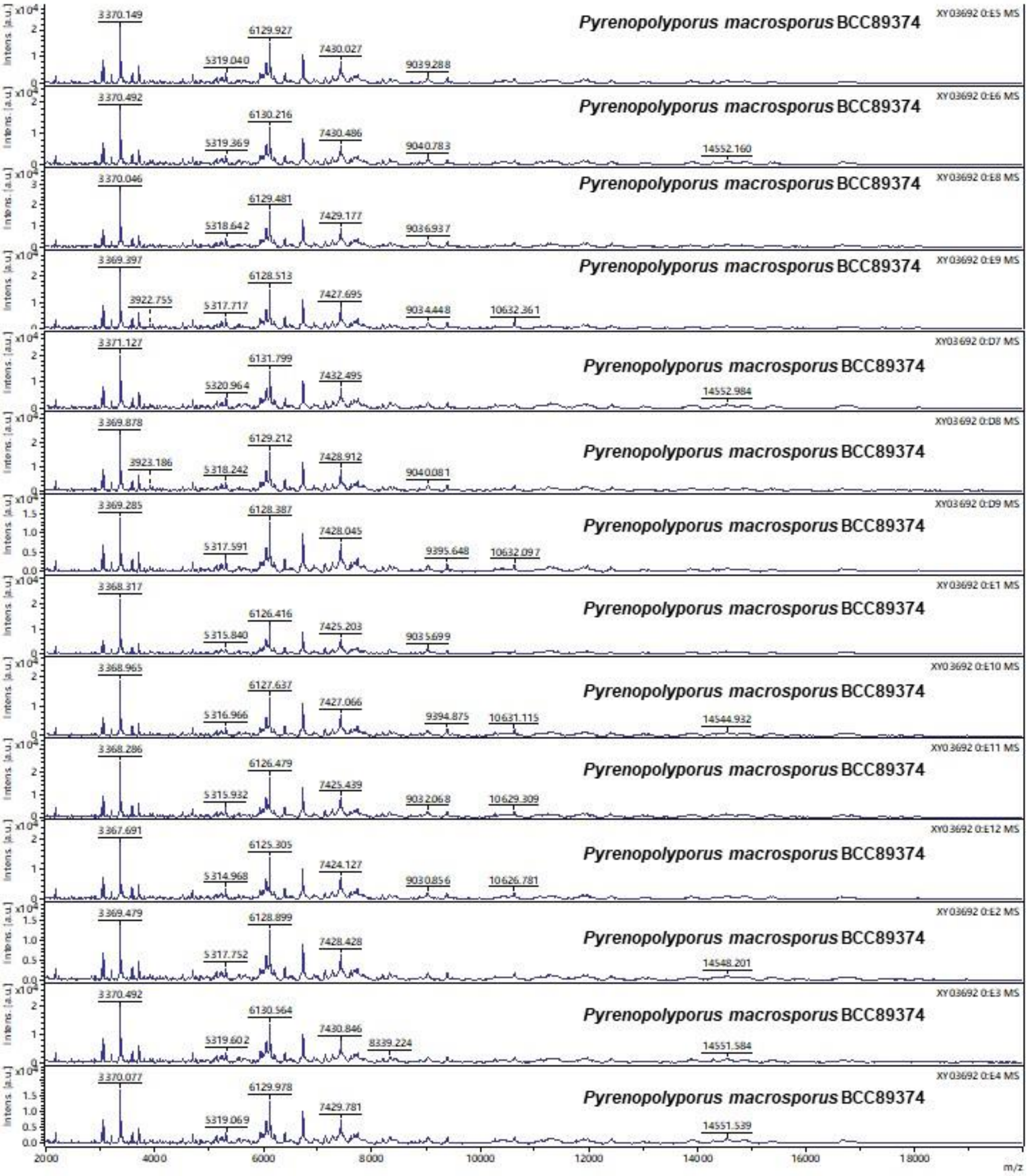
Pyrenopolyporus macrosporus BCC89374
XY03692 0:E5 MS
3370.149
6129.927
7430.027
5319.040
9039.288
XY03692 0:E6 MS
3370.492
6130.216
7430.486
5319.369
9040.783
14552.160
XY03692 0:E8 MS
3370.046
6129.481
7429.177
5318.642
9036.937
XY03692 0:E9 MS
3369.397
6128.513
7427.695
3922.755
5317.717
9034.448
10632.361
XY03692 0:D7 MS
3371.127
6131.799
7432.495
5320.964
14552.984
XY03692 0:D8 MS
3369.878
6129.212
7428.912
3923.186
5318.242
9040.081
XY03692 0:D9 MS
3369.285
6128.387
7428.045
5317.591
9395.648
10632.097
XY03692 0:E1 MS
3368.317
6126.416
7425.203
5315.840
9035.699
XY03692 0:E10 MS
3368.965
6127.637
7427.066
5316.966
9394.875
10631.115
14544.932
XY03692 0:E11 MS
3368.286
6126.479
7425.439
5315.932
9032.068
10629.309
XY03692 0:E12 MS
3367.691
6125.305
7424.127
5314.968
9030.856
10626.781
XY03692 0:E2 MS
3369.479
6128.899
7428.428
5317.752
14548.201
XY03692 0:E3 MS
3370.492
6130.564
7430.846
5319.602
8339.224
14551.584
XY03692 0:E4 MS
3370.077
6129.978
7429.781
5319.069
14551.539
Intens. [a.u.]
x10^4
2000
4000
6000
8000
10000
12000
14000
16000
18000
m/z

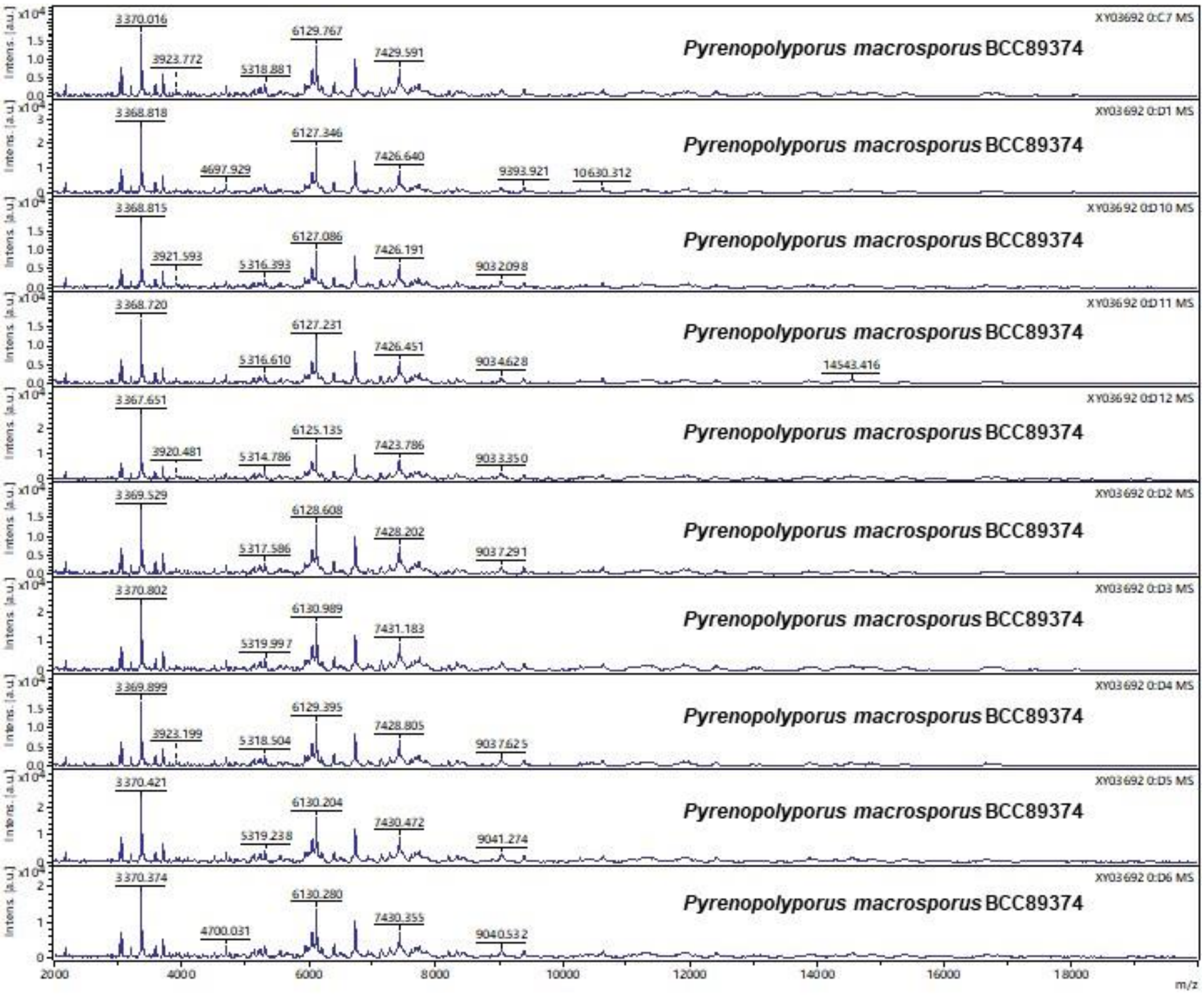

XY03692 0:C7 MS
Pyrenopolyporus macrosporus BCC89374
3370.016
3923.772
5318.881
6129.767
7429.591
XY03692 0:D1 MS
Pyrenopolyporus macrosporus BCC89374
3368.818
4697.929
6127.346
7426.640
9393.921
10630.312
XY03692 0:D10 MS
Pyrenopolyporus macrosporus BCC89374
3368.815
3921.593
5316.393
6127.086
7426.191
9032.098
XY03692 0:D11 MS
Pyrenopolyporus macrosporus BCC89374
3368.720
5316.610
6127.231
7426.451
9034.628
14543.416
XY03692 0:D12 MS
Pyrenopolyporus macrosporus BCC89374
3367.651
3920.481
5314.786
6125.135
7423.786
9033.350
XY03692 0:D2 MS
Pyrenopolyporus macrosporus BCC89374
3369.529
5317.586
6128.608
7428.202
9037.291
XY03692 0:D3 MS
Pyrenopolyporus macrosporus BCC89374
3370.802
5319.997
6130.989
7431.183
XY03692 0:D4 MS
Pyrenopolyporus macrosporus BCC89374
3369.899
3923.199
5318.504
6129.395
7428.805
9037.625
XY03692 0:D5 MS
Pyrenopolyporus macrosporus BCC89374
3370.421
5319.238
6130.204
7430.472
9041.274
XY03692 0:D6 MS
Pyrenopolyporus macrosporus BCC89374
3370.374
4700.031
6130.280
7430.355
9040.532
Intens. [a.u.]
x10^4
2000
4000
6000
8000
10000
12000
14000
16000
18000
m/z

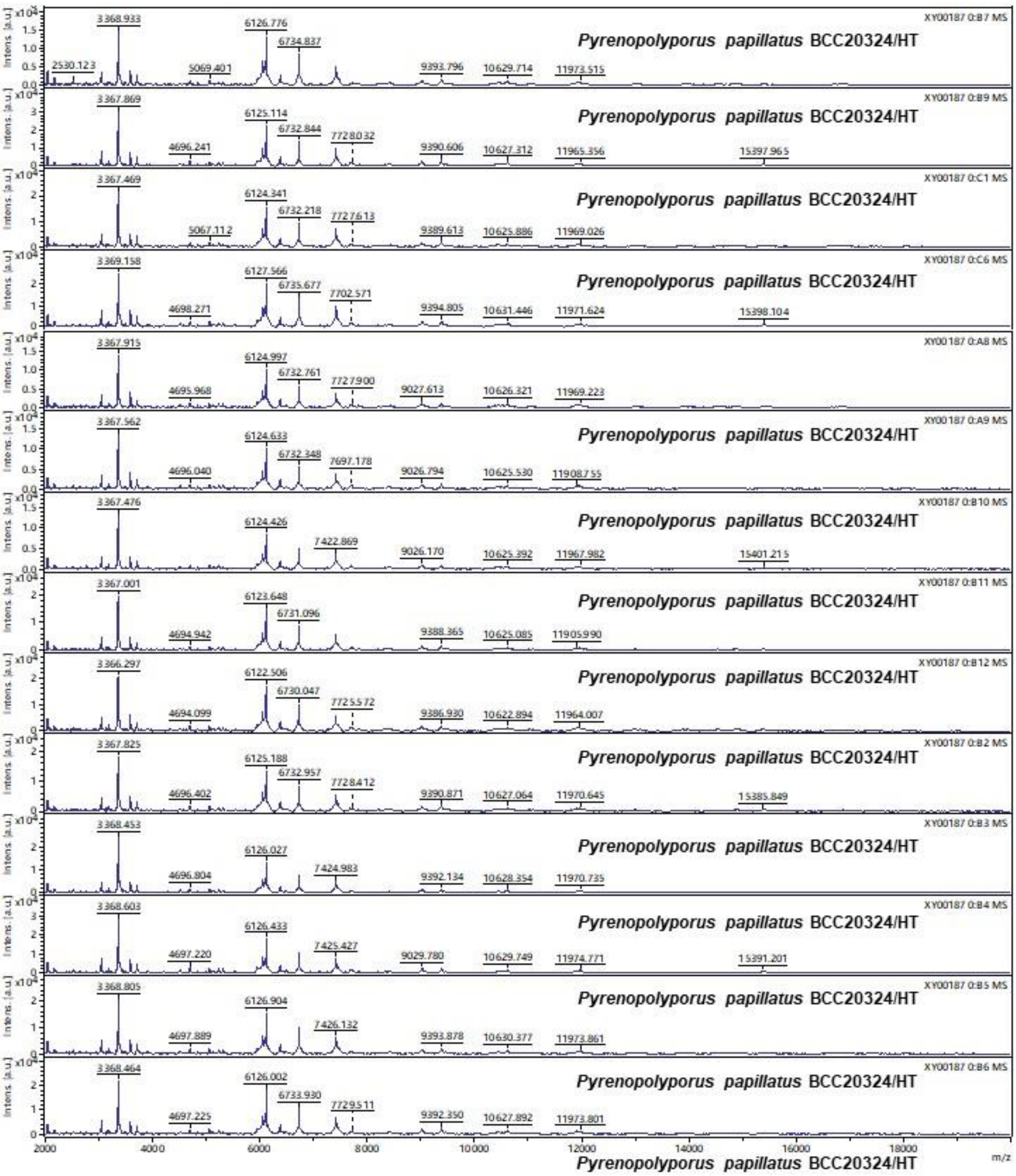

Pyrenopolyporus papillatus BCC20324/HT

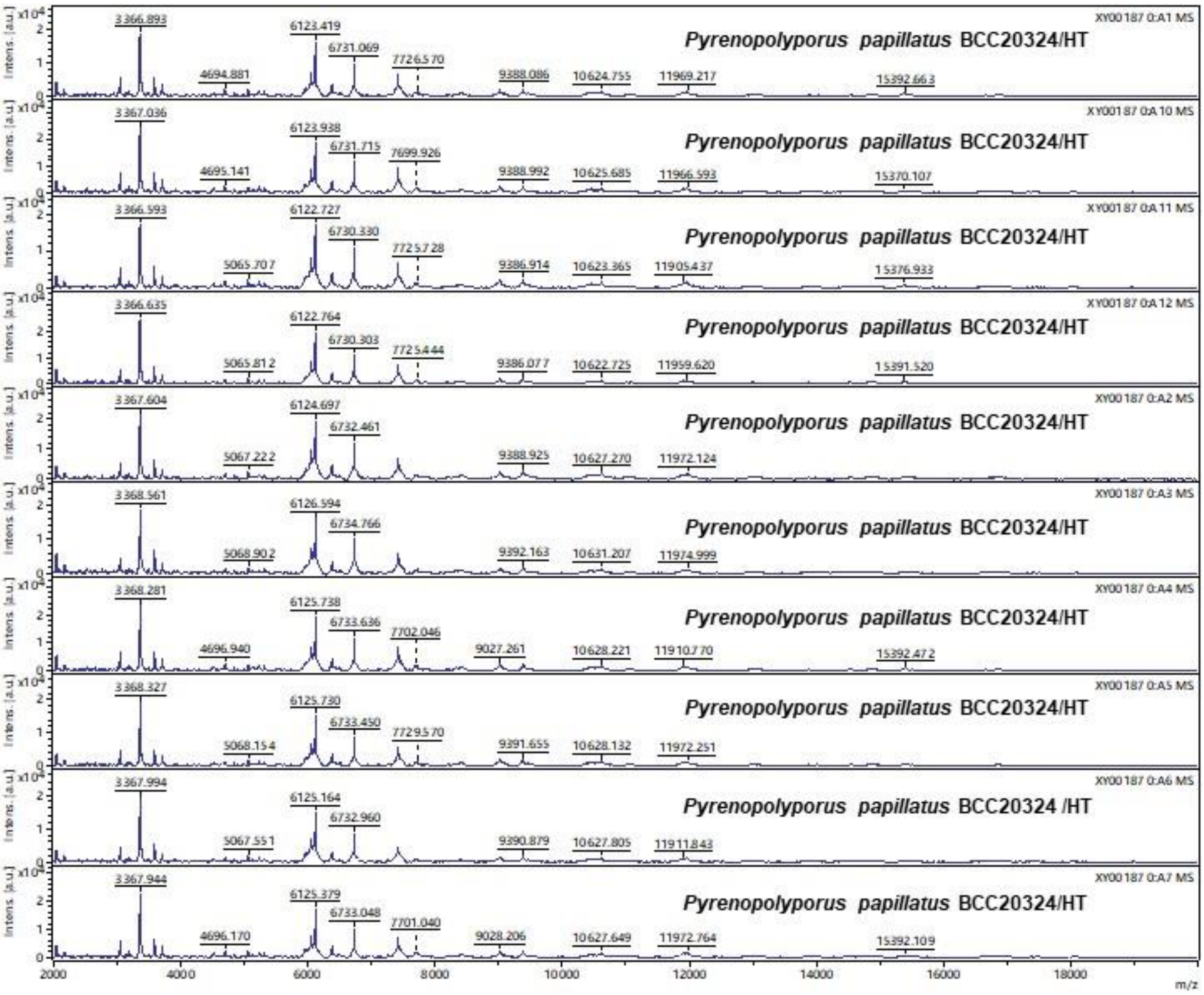
XY00187 0:A1 MS
Pyrenopolyporus papillatus BCC20324/HT
3366.893
4694.881
6123.419
6731.069
7726.570
9388.086
10624.755
11969.217
15392.663
XY00187 0:A10 MS
Pyrenopolyporus papillatus BCC20324/HT
3367.036
4695.141
6123.938
6731.715
7699.926
9388.992
10625.685
11966.593
15370.107
XY00187 0:A11 MS
Pyrenopolyporus papillatus BCC20324/HT
3366.593
5065.707
6122.727
6730.330
7725.728
9386.914
10623.365
11905.437
15376.933
XY00187 0:A12 MS
Pyrenopolyporus papillatus BCC20324/HT
3366.635
5065.812
6122.764
6730.303
7725.444
9386.077
10622.725
11959.620
15391.520
XY00187 0:A2 MS
Pyrenopolyporus papillatus BCC20324/HT
3367.604
5067.222
6124.697
6732.461
9388.925
10627.270
11972.124
XY00187 0:A3 MS
Pyrenopolyporus papillatus BCC20324/HT
3368.561
5068.902
6126.594
6734.766
9392.163
10631.207
11974.999
XY00187 0:A4 MS
Pyrenopolyporus papillatus BCC20324/HT
3368.281
4696.940
6125.738
6733.636
7702.046
9027.261
10628.221
11910.770
15392.472
XY00187 0:A5 MS
Pyrenopolyporus papillatus BCC20324/HT
3368.327
5068.154
6125.730
6733.450
7729.570
9391.655
10628.132
11972.251
XY00187 0:A6 MS
Pyrenopolyporus papillatus BCC20324 /HT
3367.994
5067.551
6125.164
6732.960
9390.879
10627.805
11911.843
XY00187 0:A7 MS
Pyrenopolyporus papillatus BCC20324/HT
3367.944
4696.170
6125.379
6733.048
7701.040
9028.206
10627.649
11972.764
15392.109
Intens. [a.u.]
x10^4
2000
4000
6000
8000
10000
12000
14000
16000
18000
m/z

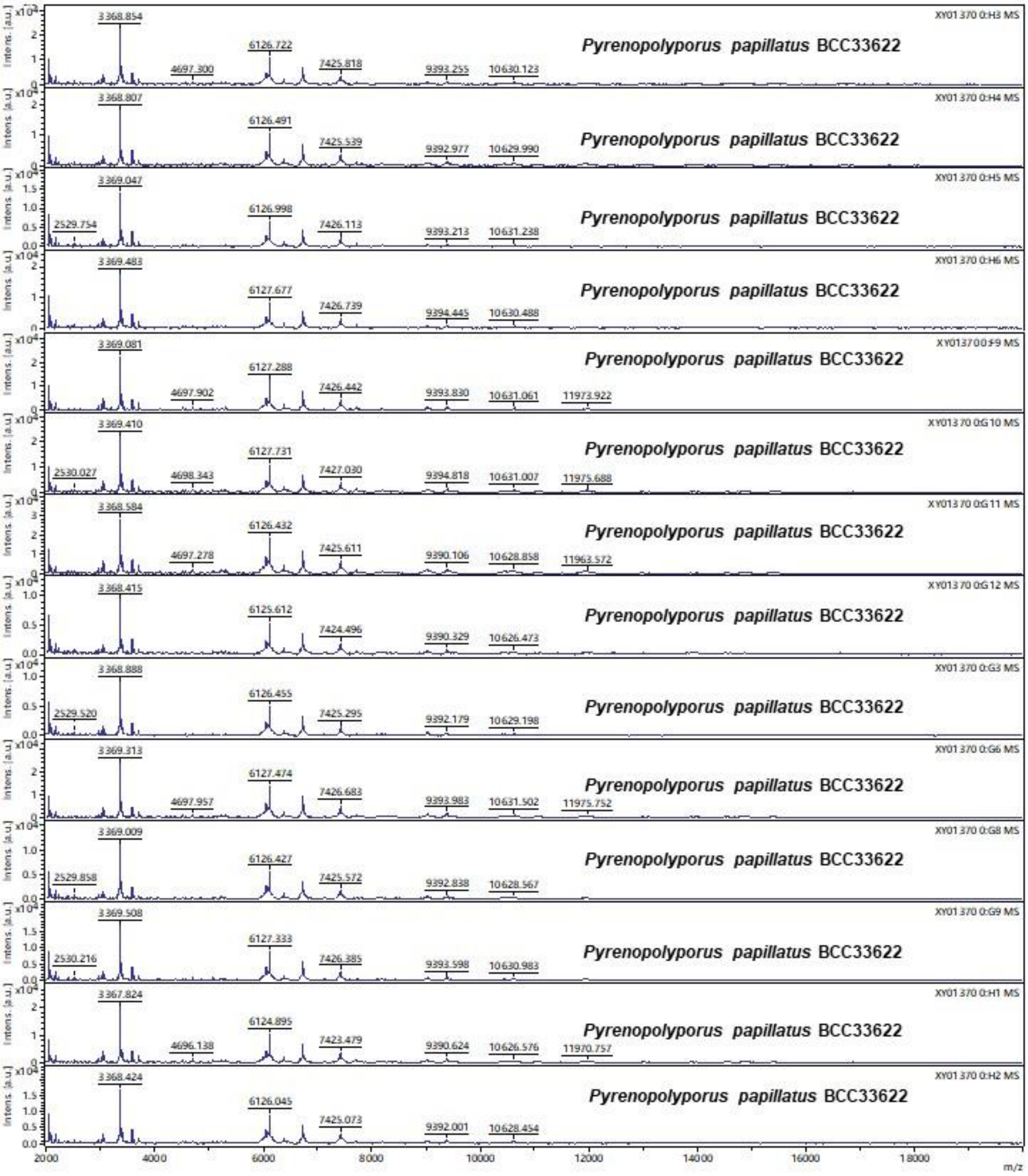
Intens. [a.u.]
x10^4
3368.854
4697.300
6126.722
7425.818
9393.255
10630.123
XY01370 0:H3 MS
Pyrenopolyporus papillatus BCC33622
3368.807
6126.491
7425.539
9392.977
10629.990
XY01370 0:H4 MS
Pyrenopolyporus papillatus BCC33622
3369.047
2529.754
6126.998
7426.113
9393.213
10631.238
XY01370 0:H5 MS
Pyrenopolyporus papillatus BCC33622
3369.483
6127.677
7426.739
9394.445
10630.488
XY01370 0:H6 MS
Pyrenopolyporus papillatus BCC33622
3369.081
4697.902
6127.288
7426.442
9393.830
10631.061
11973.922
Pyrenopolyporus papillatus BCC33622
3369.410
2530.027
4698.343
6127.731
7427.030
9394.818
10631.007
11975.688
XY01370 0:G10 MS
Pyrenopolyporus papillatus BCC33622
3368.584
4697.278
6126.432
7425.611
9390.106
10628.858
11963.572
XY01370 0:G11 MS
Pyrenopolyporus papillatus BCC33622
3368.415
6125.612
7424.496
9390.329
10626.473
XY01370 0:G12 MS
Pyrenopolyporus papillatus BCC33622
3368.888
2529.520
6126.455
7425.295
9392.179
10629.198
XY01370 0:G3 MS
Pyrenopolyporus papillatus BCC33622
3369.313
4697.957
6127.474
7426.683
9393.983
10631.502
11975.752
XY01370 0:G6 MS
Pyrenopolyporus papillatus BCC33622
3369.009
2529.858
6126.427
7425.572
9392.838
10628.567
XY01370 0:G8 MS
Pyrenopolyporus papillatus BCC33622
3369.508
2530.216
6127.333
7426.385
9393.598
10630.983
XY01370 0:G9 MS
Pyrenopolyporus papillatus BCC33622
3367.824
4696.138
6124.895
7423.479
9390.624
10626.576
11970.757
XY01370 0:H1 MS
Pyrenopolyporus papillatus BCC33622
3368.424
6126.045
7425.073
9392.001
10628.454
XY01370 0:H2 MS
Pyrenopolyporus papillatus BCC33622
2000
4000
6000
8000
10000
12000
14000
16000
18000
m/z

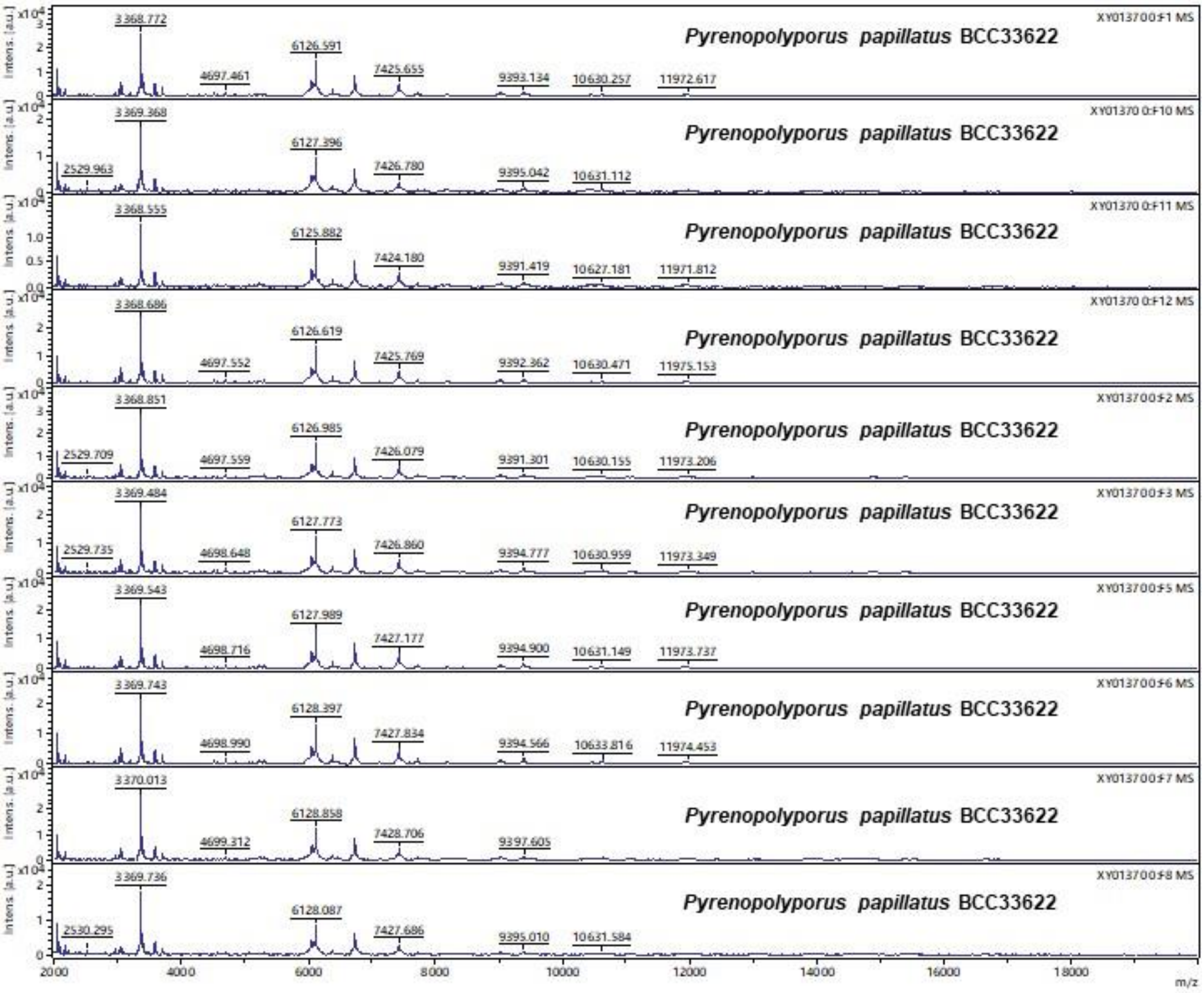

XY013700:F1 MS
Pyrenopolyporus papillatus BCC33622
3368.772
4697.461
6126.591
7425.655
9393.134
10630.257
11972.617
XY01370 0:F10 MS
Pyrenopolyporus papillatus BCC33622
2529.963
3369.368
6127.396
7426.780
9395.042
10631.112
XY01370 0:F11 MS
Pyrenopolyporus papillatus BCC33622
3368.555
6125.882
7424.180
9391.419
10627.181
11971.812
XY01370 0:F12 MS
Pyrenopolyporus papillatus BCC33622
3368.686
4697.552
6126.619
7425.769
9392.362
10630.471
11975.153
XY013700:F2 MS
Pyrenopolyporus papillatus BCC33622
2529.709
3368.851
4697.559
6126.985
7426.079
9391.301
10630.155
11973.206
XY013700:F3 MS
Pyrenopolyporus papillatus BCC33622
2529.735
3369.484
4698.648
6127.773
7426.860
9394.777
10630.959
11973.349
XY013700:F5 MS
Pyrenopolyporus papillatus BCC33622
3369.543
4698.716
6127.989
7427.177
9394.900
10631.149
11973.737
XY013700:F6 MS
Pyrenopolyporus papillatus BCC33622
3369.743
4698.990
6128.397
7427.834
9394.566
10633.816
11974.453
XY013700:F7 MS
Pyrenopolyporus papillatus BCC33622
3370.013
4699.312
6128.858
7428.706
9397.605
XY013700:F8 MS
Pyrenopolyporus papillatus BCC33622
2530.295
3369.736
6128.087
7427.686
9395.010
10631.584
Intens. [a.u.]
x10^4
2000
4000
6000
8000
10000
12000
14000
16000
18000
m/z

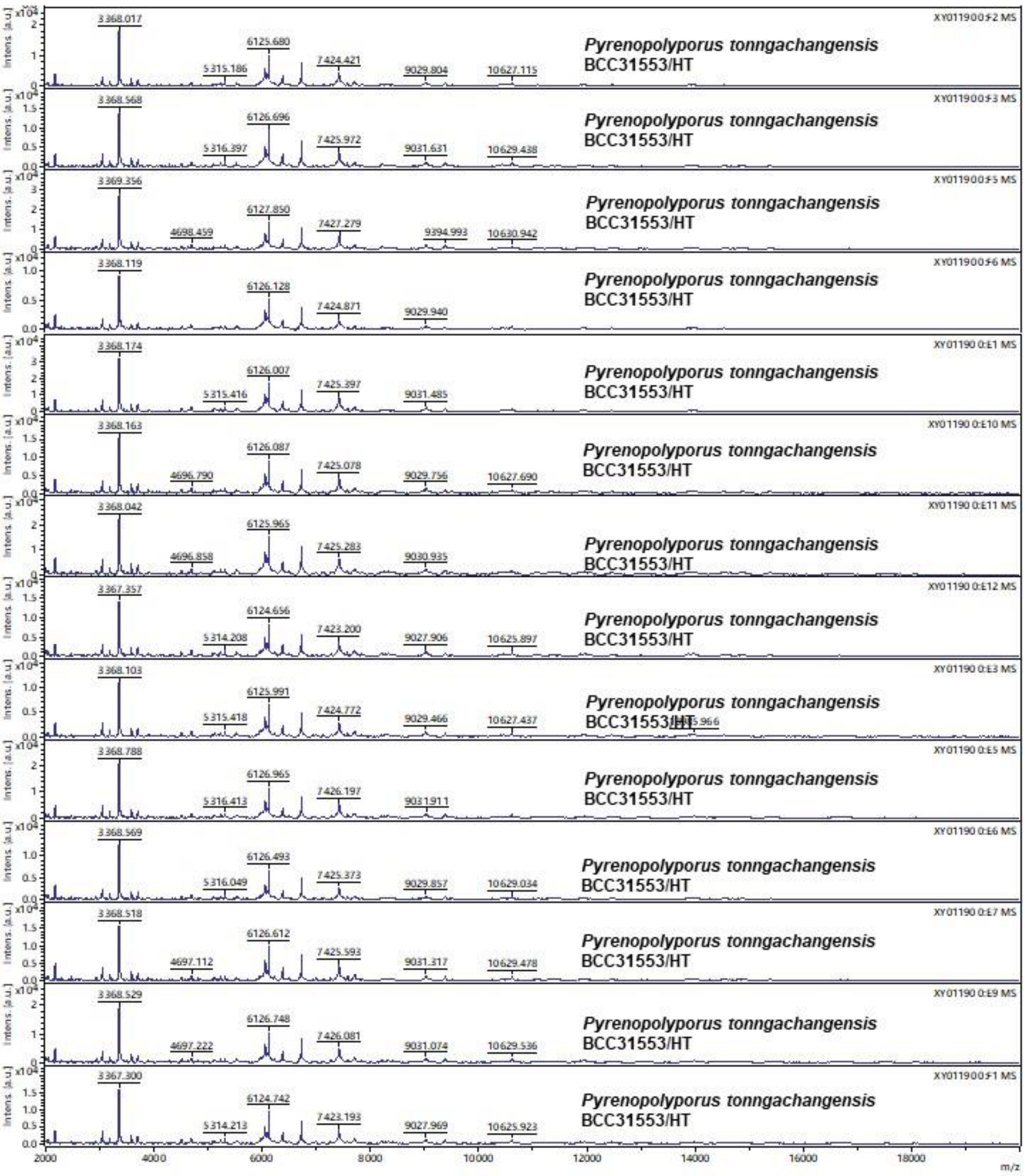
Pyrenopolyporus tonngachangensis
BCC31553/HT
Intens. [a.u.]
m/z

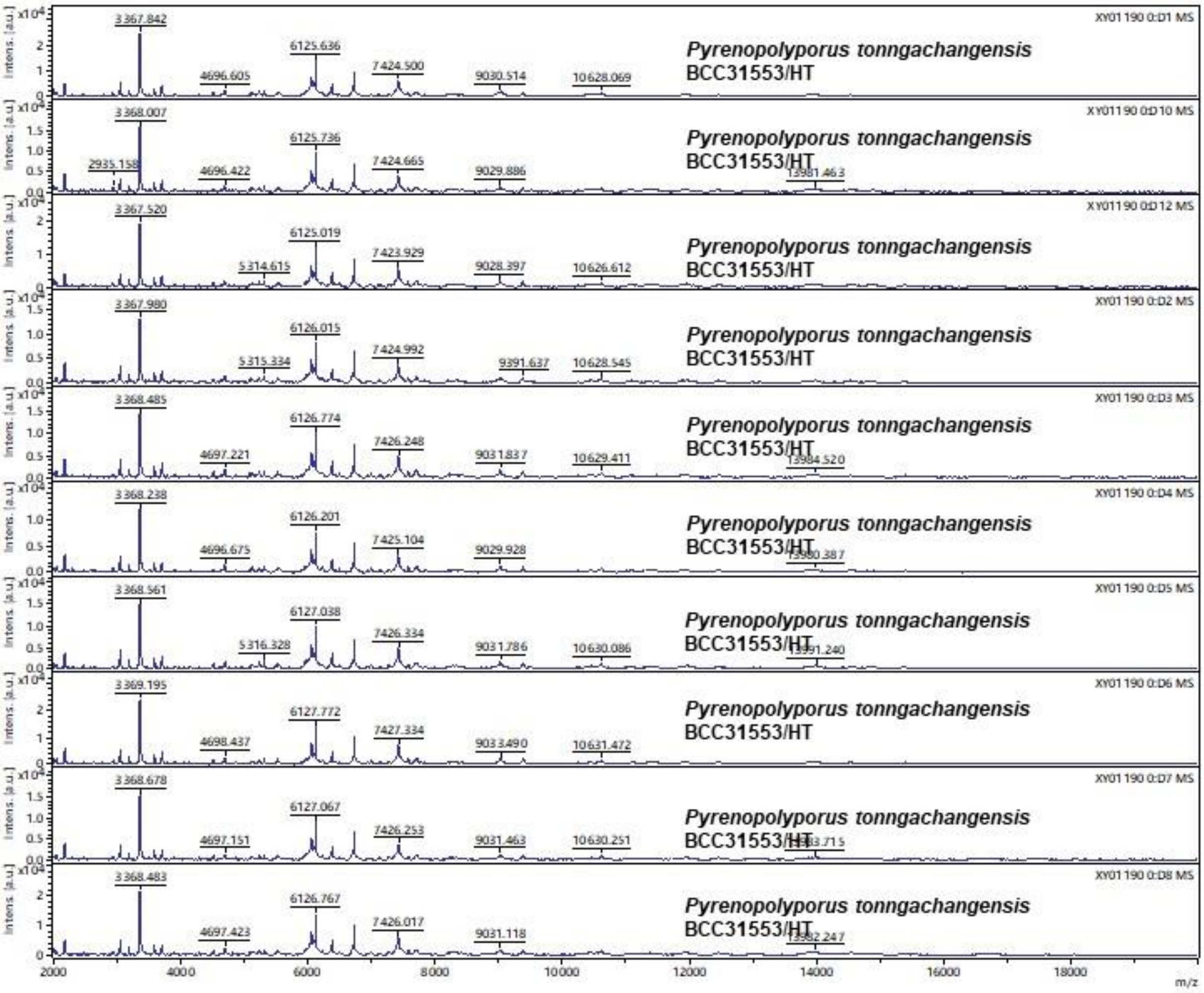
XY01190 0:D1 MS
3367.842
6125.636
7424.500
4696.605
9030.514
10628.069
Pyrenopolyporus tonngachangensis
BCC31553/HT
XY01190 0:D10 MS
3368.007
6125.736
2935.158
7424.665
4696.422
9029.886
13981.463
XY01190 0:D12 MS
3367.520
6125.019
7423.929
5314.615
9028.397
10626.612
XY01190 0:D2 MS
3367.980
6126.015
7424.992
5315.334
9391.637
10628.545
XY01190 0:D3 MS
3368.485
6126.774
7426.248
4697.221
9031.837
10629.411
13984.520
XY01190 0:D4 MS
3368.238
6126.201
7425.104
4696.675
9029.928
XY01190 0:D5 MS
3368.561
6127.038
7426.334
5316.328
9031.786
10630.086
XY01190 0:D6 MS
3369.195
6127.772
7427.334
4698.437
9033.490
10631.472
XY01190 0:D7 MS
3368.678
6127.067
7426.253
4697.151
9031.463
10630.251
XY01190 0:D8 MS
3368.483
6126.767
7426.017
4697.423
9031.118
Intens. [a.u.]
x10⁴
m/z
2000
4000
6000
8000
10000
12000
14000
16000
18000

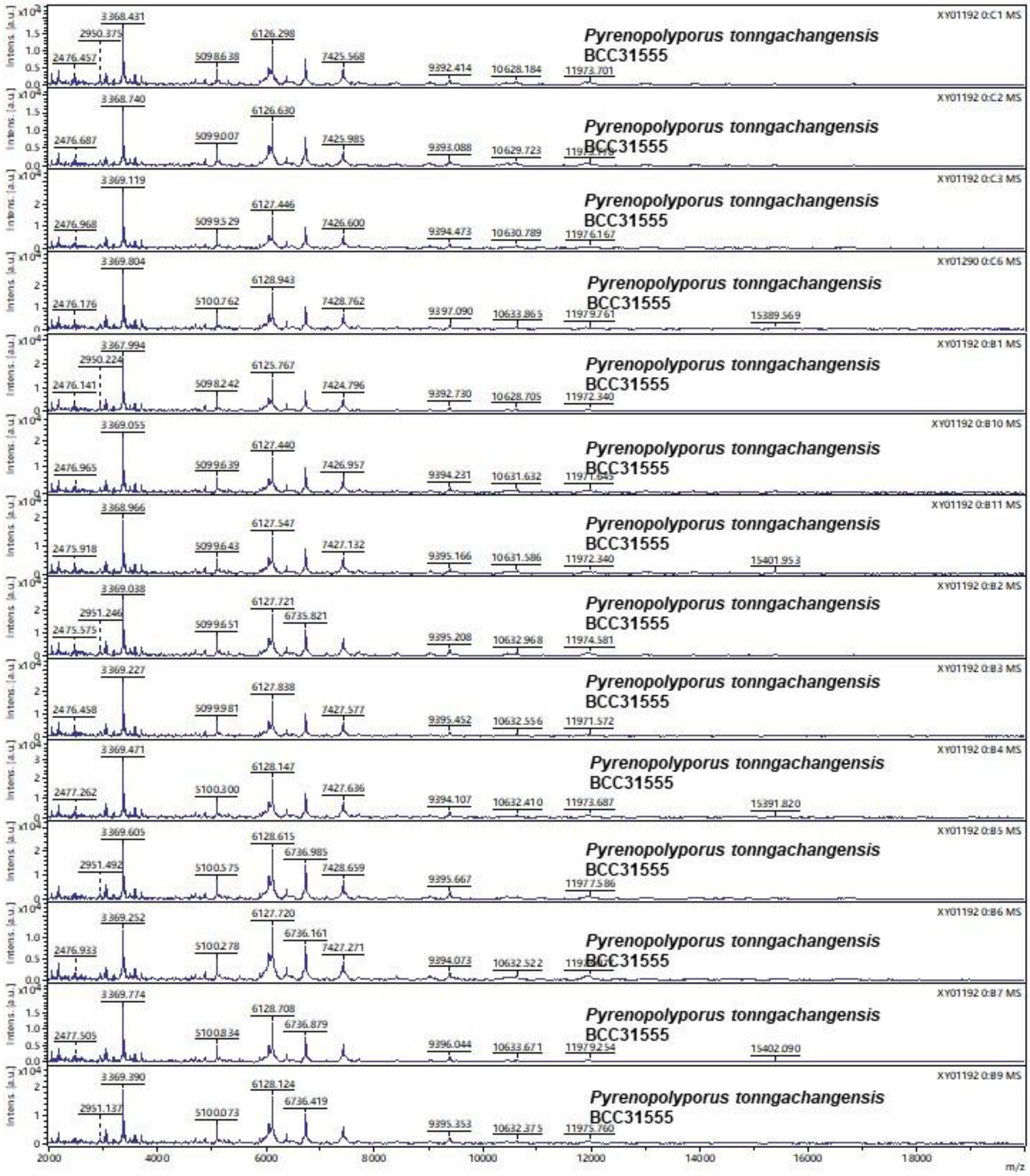
Pyrenopolyporus tonngachangensis
BCC31555
XY01192 0:C1 MS
XY01192 0:C2 MS
XY01192 0:C3 MS
XY01290 0:C6 MS
XY01192 0:B1 MS
XY01192 0:B10 MS
XY01192 0:B11 MS
XY01192 0:B2 MS
XY01192 0:B3 MS
XY01192 0:B4 MS
XY01192 0:B5 MS
XY01192 0:B6 MS
XY01192 0:B7 MS
XY01192 0:B9 MS
Intens. [a.u.]
m/z

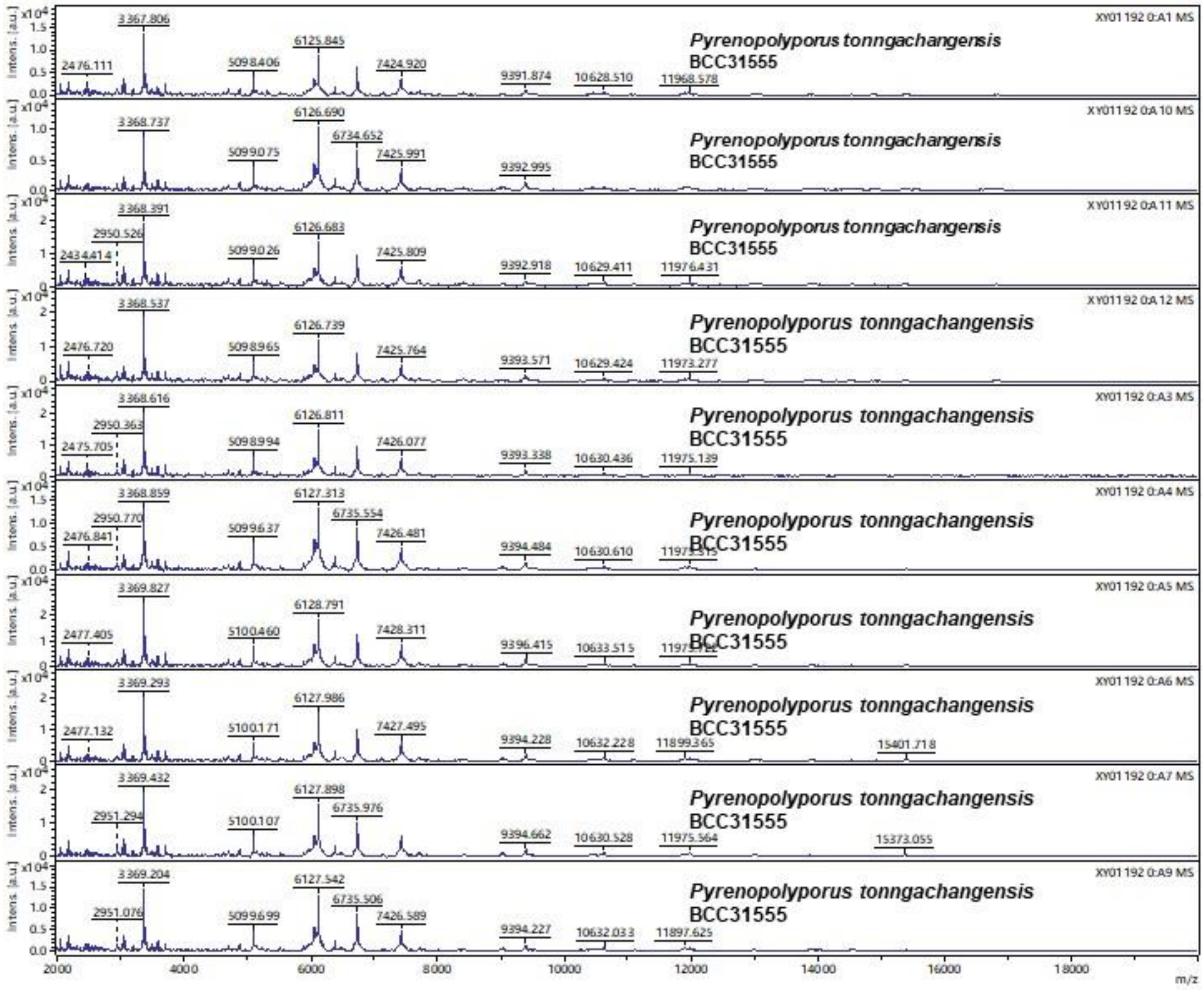
Pyrenopolyporus tonngachangensis
BCC31555
XY01192 0:A1 MS
XY01192 0:A10 MS
XY01192 0:A11 MS
XY01192 0:A12 MS
XY01192 0:A3 MS
XY01192 0:A4 MS
XY01192 0:A5 MS
XY01192 0:A6 MS
XY01192 0:A7 MS
XY01192 0:A9 MS
Intens. [a.u.]
m/z

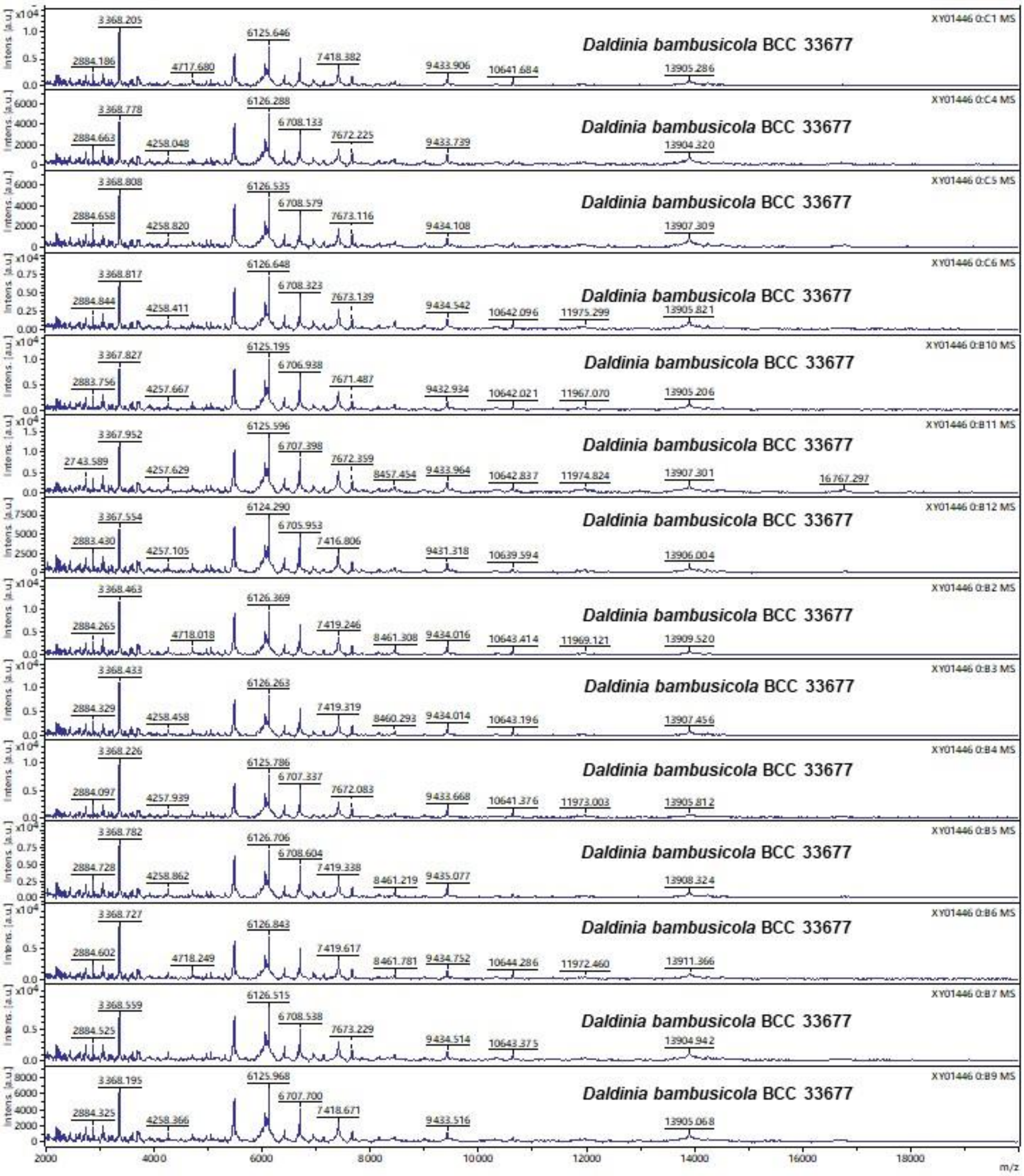
XY01446 0:C1 MS
Daldinia bambusicola BCC 33677
3368.205
2884.186
4717.680
6125.646
7418.382
9433.906
10641.684
13905.286
XY01446 0:C4 MS
Daldinia bambusicola BCC 33677
3368.778
2884.663
4258.048
6126.288
6708.133
7672.225
9433.739
13904.320
XY01446 0:C5 MS
Daldinia bambusicola BCC 33677
3368.808
2884.658
4258.820
6126.535
6708.579
7673.116
9434.108
13907.309
XY01446 0:C6 MS
Daldinia bambusicola BCC 33677
3368.817
2884.844
4258.411
6126.648
6708.323
7673.139
9434.542
10642.096
11975.299
13905.821
XY01446 0:B10 MS
Daldinia bambusicola BCC 33677
3367.827
2883.756
4257.667
6125.195
6706.938
7671.487
9432.934
10642.021
11967.070
13905.206
XY01446 0:B11 MS
Daldinia bambusicola BCC 33677
3367.952
2743.589
4257.629
6125.596
6707.398
7672.359
8457.454
9433.964
10642.837
11974.824
13907.301
16767.297
XY01446 0:B12 MS
Daldinia bambusicola BCC 33677
3367.554
2883.430
4257.105
6124.290
6705.953
7416.806
9431.318
10639.594
13906.004
XY01446 0:B2 MS
Daldinia bambusicola BCC 33677
3368.463
2884.265
4718.018
6126.369
7419.246
8461.308
9434.016
10643.414
11969.121
13909.520
XY01446 0:B3 MS
Daldinia bambusicola BCC 33677
3368.433
2884.329
4258.458
6126.263
7419.319
8460.293
9434.014
10643.196
13907.456
XY01446 0:B4 MS
Daldinia bambusicola BCC 33677
3368.226
2884.097
4257.939
6125.786
6707.337
7672.083
9433.668
10641.376
11973.003
13905.812
XY01446 0:B5 MS
Daldinia bambusicola BCC 33677
3368.782
2884.728
4258.862
6126.706
6708.604
7419.338
8461.219
9435.077
13908.324
XY01446 0:B6 MS
Daldinia bambusicola BCC 33677
3368.727
2884.602
4718.249
6126.843
7419.617
8461.781
9434.752
10644.286
11972.460
13911.366
XY01446 0:B7 MS
Daldinia bambusicola BCC 33677
3368.559
2884.525
6126.515
6708.538
7673.229
9434.514
10643.375
13904.942
XY01446 0:B9 MS
Daldinia bambusicola BCC 33677
3368.195
2884.325
4258.366
6125.968
6707.700
7418.671
9433.516
13905.068
Intens. [a.u.]
m/z
2000
4000
6000
8000
10000
12000
14000
16000
18000

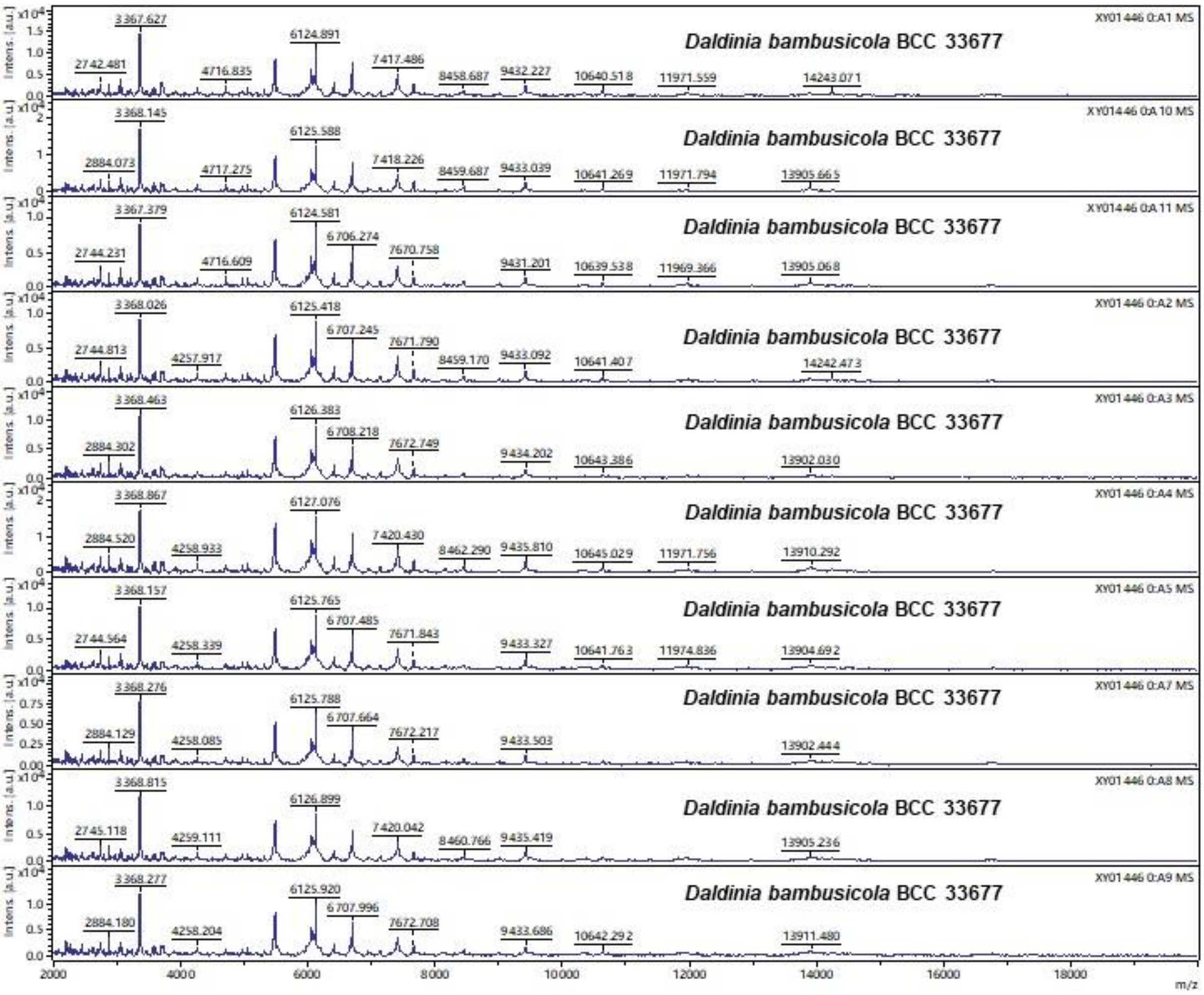

Daldinia bambusicola BCC 33677
XY01446 0:A1 MS
3367.627
2742.481
4716.835
6124.891
7417.486
8458.687
9432.227
10640.518
11971.559
14243.071
XY01446 0:A10 MS
3368.145
2884.073
4717.275
6125.588
7418.226
8459.687
9433.039
10641.269
11971.794
13905.665
XY01446 0:A11 MS
3367.379
2744.231
4716.609
6124.581
6706.274
7670.758
9431.201
10639.538
11969.366
13905.068
XY01446 0:A2 MS
3368.026
2744.813
4257.917
6125.418
6707.245
7671.790
8459.170
9433.092
10641.407
14242.473
XY01446 0:A3 MS
3368.463
2884.302
6126.383
6708.218
7672.749
9434.202
10643.386
13902.030
XY01446 0:A4 MS
3368.867
2884.520
4258.933
6127.076
7420.430
8462.290
9435.810
10645.029
11971.756
13910.292
XY01446 0:A5 MS
3368.157
2744.564
4258.339
6125.765
6707.485
7671.843
9433.327
10641.763
11974.836
13904.692
XY01446 0:A7 MS
3368.276
2884.129
4258.085
6125.788
6707.664
7672.217
9433.503
13902.444
XY01446 0:A8 MS
3368.815
2745.118
4259.111
6126.899
7420.042
8460.766
9435.419
13905.236
XY01446 0:A9 MS
3368.277
2884.180
4258.204
6125.920
6707.996
7672.708
9433.686
10642.292
13911.480
Intens. [a.u.]
x10⁴
m/z
2000
4000
6000
8000
10000
12000
14000
16000
18000

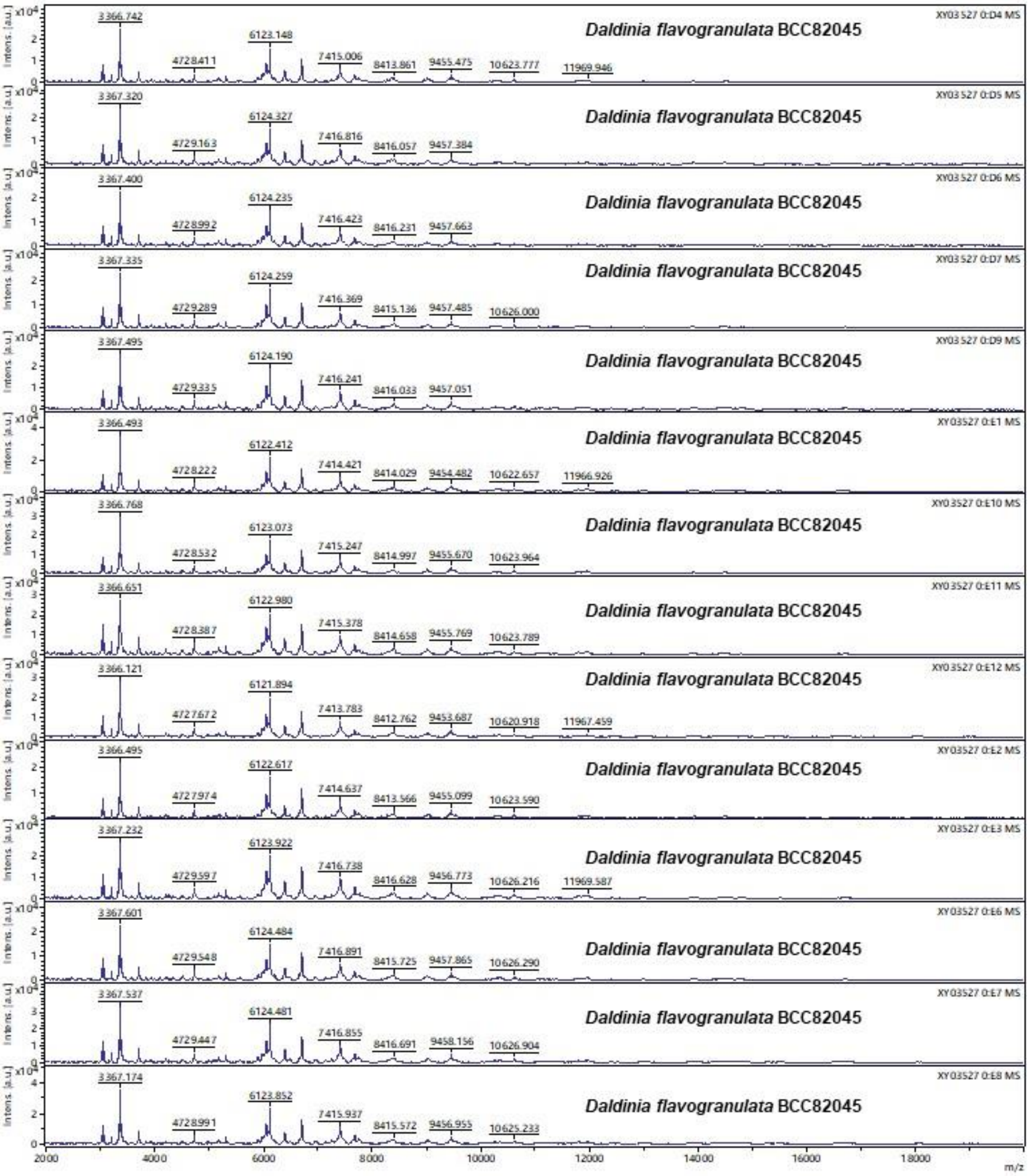

Daldinia flavogranulata BCC82045
XY03527 0:D4 MS
3366.742
6123.148
4728.411
7415.006
8413.861
9455.475
10623.777
11969.946
XY03527 0:D5 MS
3367.320
6124.327
4729.163
7416.816
8416.057
9457.384
XY03527 0:D6 MS
3367.400
6124.235
4728.992
7416.423
8416.231
9457.663
XY03527 0:D7 MS
3367.335
6124.259
4729.289
7416.369
8415.136
9457.485
10626.000
XY03527 0:D9 MS
3367.495
6124.190
4729.335
7416.241
8416.033
9457.051
XY03527 0:E1 MS
3366.493
6122.412
4728.222
7414.421
8414.029
9454.482
10622.657
11966.926
XY03527 0:E10 MS
3366.768
6123.073
4728.532
7415.247
8414.997
9455.670
10623.964
XY03527 0:E11 MS
3366.651
6122.980
4728.387
7415.378
8414.658
9455.769
10623.789
XY03527 0:E12 MS
3366.121
6121.894
4727.672
7413.783
8412.762
9453.687
10620.918
11967.459
XY03527 0:E2 MS
3366.495
6122.617
4727.974
7414.637
8413.566
9455.099
10623.590
XY03527 0:E3 MS
3367.232
6123.922
4729.597
7416.738
8416.628
9456.773
10626.216
11969.587
XY03527 0:E6 MS
3367.601
6124.484
4729.548
7416.891
8415.725
9457.865
10626.290
XY03527 0:E7 MS
3367.537
6124.481
4729.447
7416.855
8416.691
9458.156
10626.904
XY03527 0:E8 MS
3367.174
6123.852
4728.991
7415.937
8415.572
9456.955
10625.233
Intens. [a.u.]
x10⁴
2000
4000
6000
8000
10000
12000
14000
16000
18000
m/z

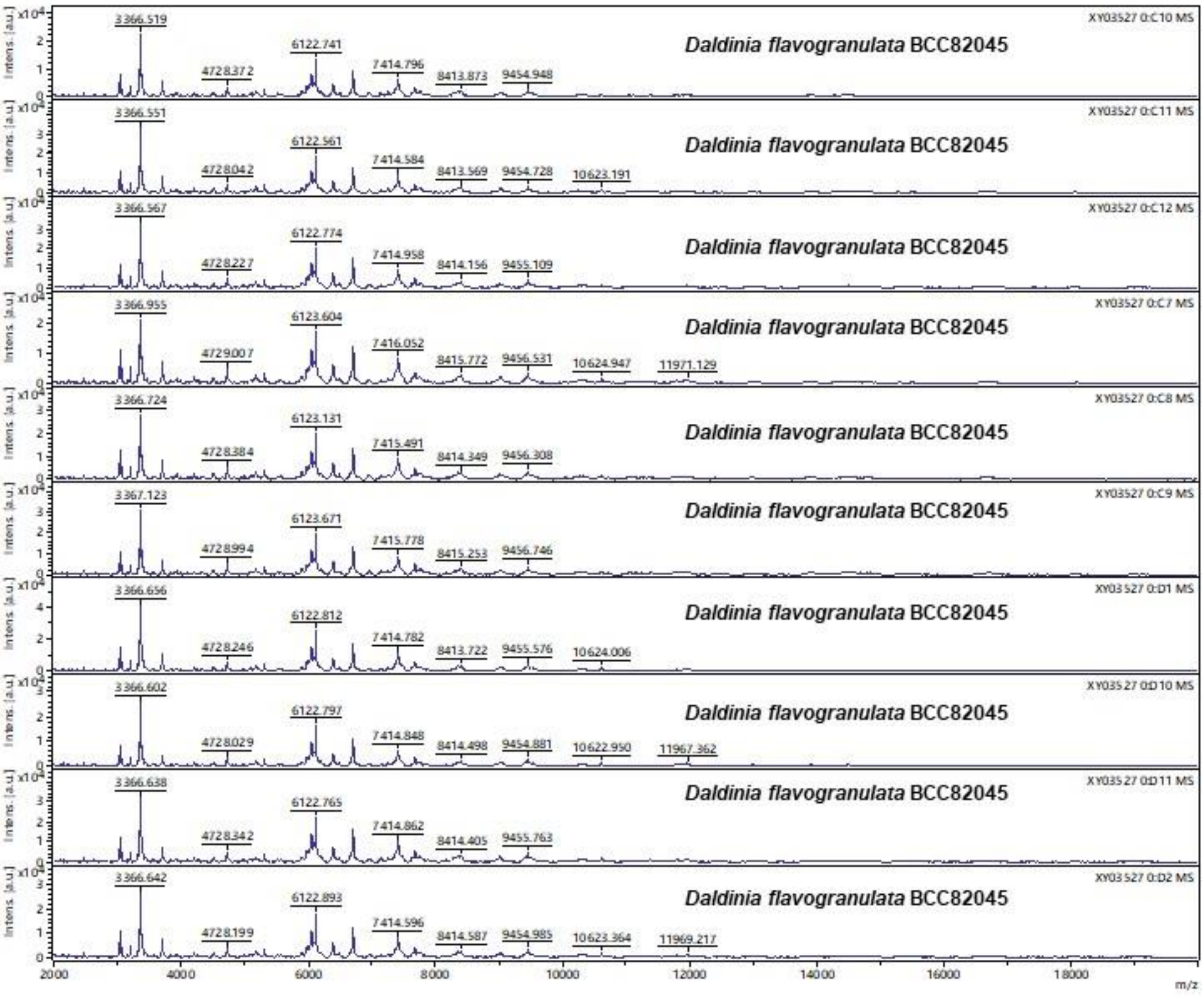
Daldinia flavogranulata BCC82045
XY03527 0:C10 MS
XY03527 0:C11 MS
XY03527 0:C12 MS
XY03527 0:C7 MS
XY03527 0:C8 MS
XY03527 0:C9 MS
XY03527 0:D1 MS
XY03527 0:D10 MS
XY03527 0:D11 MS
XY03527 0:D2 MS
3366.519
6122.741
7414.796
9454.948
3366.551
6122.561
10623.191
3366.567
6122.774
3366.955
6123.604
11971.129
3366.724
6123.131
3367.123
6123.671
3366.656
6122.812
3366.602
6122.797
11967.362
3366.638
6122.765
3366.642
6122.893
11969.217
Intens. [a.u.]
x10^4
m/z
2000
4000
6000
8000
10000
12000
14000
16000
18000

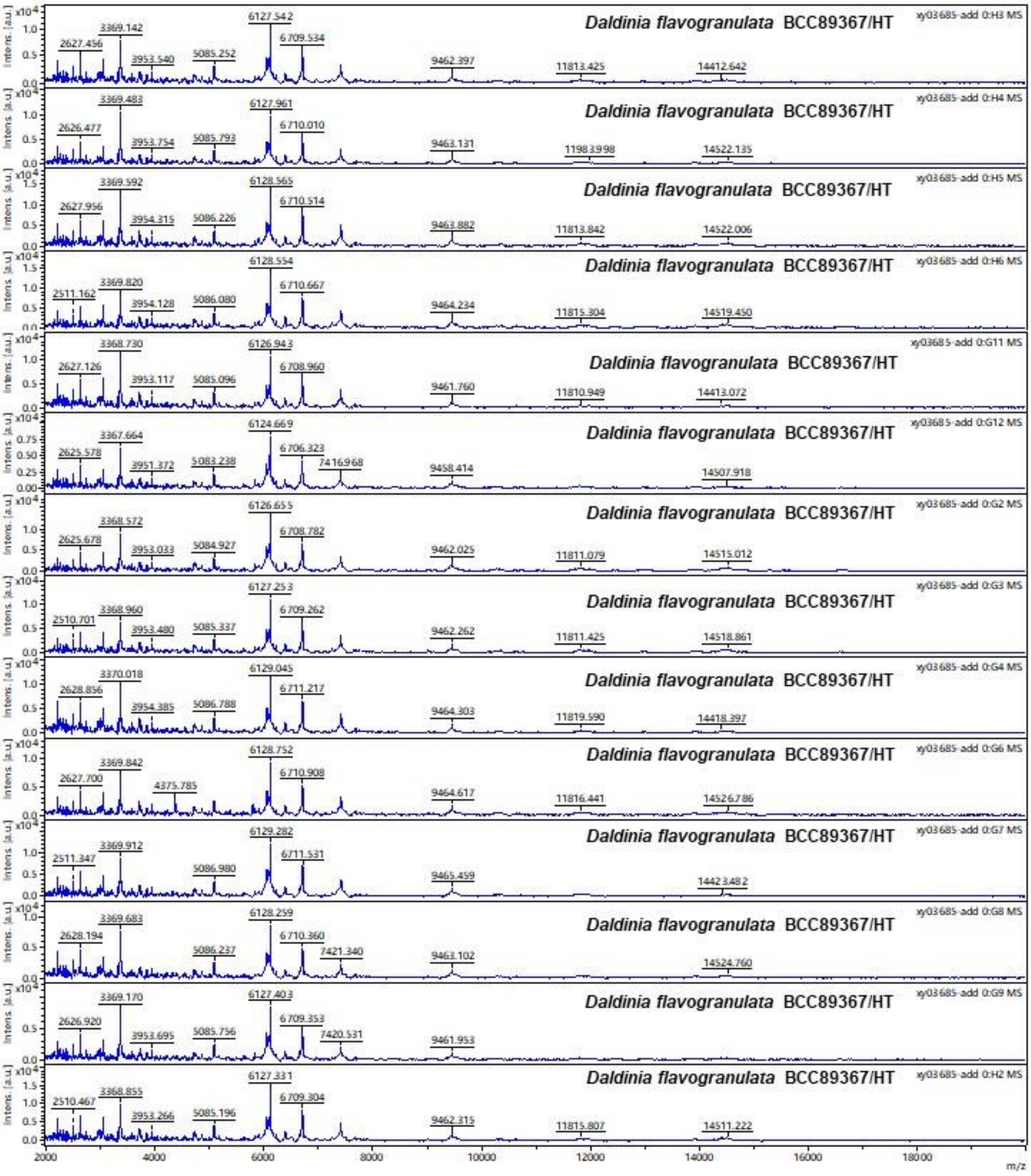

Daldinia flavogranulata BCC89367/HT
xy03685-add 0:H3 MS
3369.142
2627.456
3953.540
5085.252
6127.542
6709.534
9462.397
11813.425
14412.642
xy03685-add 0:H4 MS
3369.483
2626.477
3953.754
5085.793
6127.961
6710.010
9463.131
11983.998
14522.135
xy03685-add 0:H5 MS
3369.592
2627.956
3954.315
5086.226
6128.565
6710.514
9463.882
11813.842
14522.006
xy03685-add 0:H6 MS
2511.162
3369.820
3954.128
5086.080
6128.554
6710.667
9464.234
11815.304
14519.450
xy03685-add 0:G11 MS
3368.730
2627.126
3953.117
5085.096
6126.943
6708.960
9461.760
11810.949
14413.072
xy03685-add 0:G12 MS
3367.664
2625.578
3951.372
5083.238
6124.669
6706.323
7416.968
9458.414
14507.918
xy03685-add 0:G2 MS
3368.572
2625.678
3953.033
5084.927
6126.655
6708.782
9462.025
11811.079
14515.012
xy03685-add 0:G3 MS
2510.701
3368.960
3953.480
5085.337
6127.253
6709.262
9462.262
11811.425
14518.861
xy03685-add 0:G4 MS
3370.018
2628.856
3954.385
5086.788
6129.045
6711.217
9464.303
11819.590
14418.397
xy03685-add 0:G6 MS
3369.842
2627.700
4375.785
6128.752
6710.908
9464.617
11816.441
14526.786
xy03685-add 0:G7 MS
2511.347
3369.912
5086.980
6129.282
6711.531
9465.459
14423.482
xy03685-add 0:G8 MS
3369.683
2628.194
5086.237
6128.259
6710.360
7421.340
9463.102
14524.760
xy03685-add 0:G9 MS
3369.170
2626.920
3953.695
5085.756
6127.403
6709.353
7420.531
9461.953
xy03685-add 0:H2 MS
2510.467
3368.855
3953.266
5085.196
6127.331
6709.304
9462.315
11815.807
14511.222
Intens. [a.u.]
x10⁴
m/z
2000
4000
6000
8000
10000
12000
14000
16000
18000

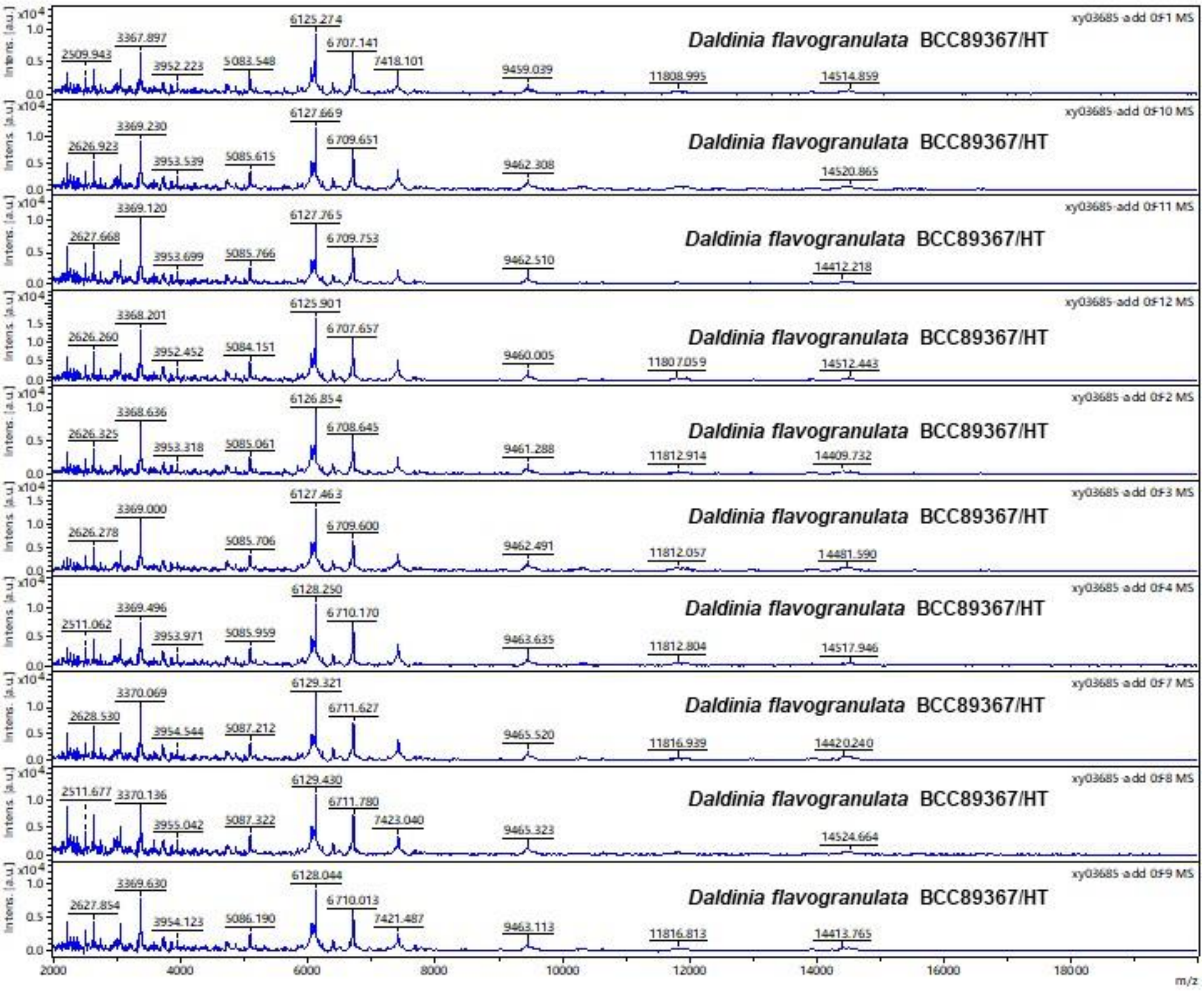

Figure S6. MALDI-TOF MASS SPECTRA.

Table S1. Comparison of morphological and chemotaxonomic features of Hypoxylaceae species with massive stroma and long tubular perithecia.

Taxon	Ostioles	Perispore	Germ Slite	Ascospore size (µm)	KOH-extractable pigments	Metabolites (stroma)
Hypoxylon begae	umbilicate	dehiscent	short, dorsal	21–29 × 12–14.5	Dense isabelline	BNT, napththols, and unknown metabolite
Daldinia placentiformis	umbilicate	dehiscent	spore length, dorsal	14.5–16 × 6.5–7	Olivaceous	BNT, napththols, naphthoquinones
D. kreztschmarioides	umbilicate	dehiscent	spore length, dorsal	(4) 5–6 × 13–15(16)	Dark Vinaceous	BNT, Cytochalasan
Pyrenopolyporus laminosus	umbilicate	**indehiscent**	spore length, dorsal	11–13.5 × 4.2–4.5	Dilute purple	BNT, napththols, naphthoquinones
P. hunteri	umbilicate	**indehiscent**	less than spore length, dorsal	11.5–14 × 5–5.5	Dense purple or absent	BNT, napththols, naphthoquinones
P. nicaraguensis	papillate	**indehiscent**	spore length, dorsal	(11–)12–15(–16) × 5–6.5	Dilute purple or absent	BNT, napththols, naphthoquinones
P. tortisporus	umbilicate	**indehiscent**	spore length, dorsal	9.5–14(–16) × 4–6	Dense amber or Olivaceous	BNT, napththols, naphthoquinones
P. sympyhon	papillate	**indehiscent**	spore length, dorsal	9.5–12(–13) × 4–5	Purple or absent	BNT, napththols, naphthoquinones, daldinal A, daldinal C
P. laminosus (this study)	umbilicate	**indehiscent**	spore length, dorsal	(12–)13–14(–15) × 4–5	Dense purple	BNT, hypoxylones
P. bambusicola (this study)	umbilicate	**indehiscent**	spore length, dorsal	8–10(–11) × (3–) 4–5	Dense purple	BNT, hypoxylones
P. macrosporus (this study)	inconspicuous	**indehiscent**	spore length, dorsal	(14–) 16–17 × (6–)7–8	Dense purple	BNT, hypoxylones
P. papillatus (this study)	papillate	**indehiscent**	spore length, dorsal	(11–) 12–13(–14) × 4–5	Dense purple	BNT, hypoxylones
P. tonngachangensi (this study)	umbilicate	**indehiscent**	2/3 spore length, dorsal	(12–) 13–14(–16) × 4–5	Dense purple	BNT, hypoxylones
P. cinereopigmentosus (this study)	inconspicuous	**indehiscent**	spore length, dorsal	(12–) 13–14 (–15) × 6–7	Grey	BNT, hypoxylones

Table S2. Dereplicated metabolites from the stromatal extracts of the *Pyrenopolyporus* spp. and in-house standards.

Compound	*m/z*	rt	CCS	Formula	Annotation*
Hypoxylone (**1**)	333.07588 $[M+H]^+$	13.42	169.2	$C_{20}H_{12}O_5$	Level 2
BNT (**2**)	319.09643 $[M+H]^+$	12.28	172.6	$C_{20}H_{14}O_4$	Level 1
3	349.07041 $[M+H]^+$	11.38	175.1	$C_{20}H_{12}O_6$	Level 2
4	258.10997 $[M+Na]^+$	8.92	159.3	$C_{13}H_{17}NO_3$	n.i.
5	633.45371 $[M+H]^+$	18.59	251.9	$C_{30}H_{60}N_6O_8$	n.i.

*Level 0 corresponds to data obtained from in-house standards; level 1 to annotations by comparison with standards; level 2 to putative annotations (i.e., MS/MS library search); level 3 to a compound class assignment; n.i. to non-identified metabolites. rt = retention time in min; CCS = collisional cross section in Å^2.

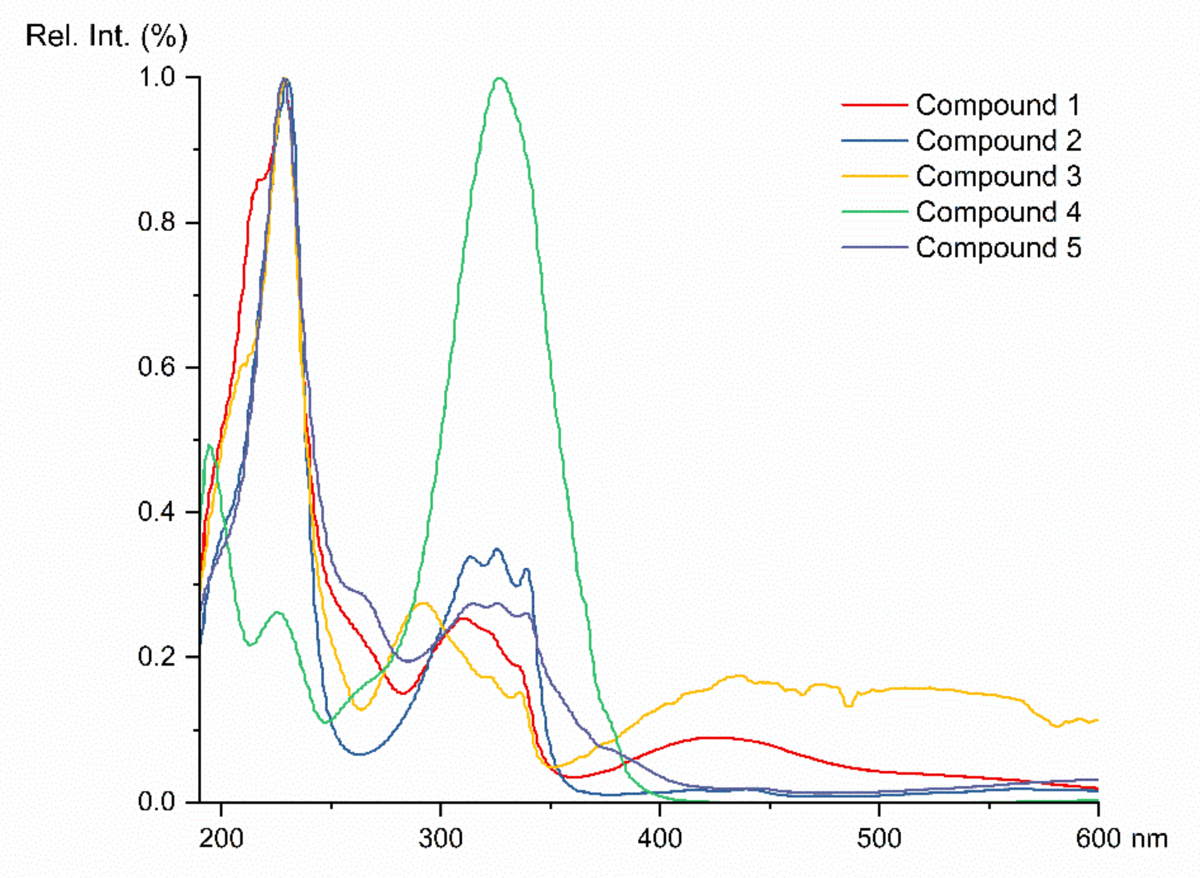

Figure S7. DAD spectra of the major metabolites (**1**–**5**) depicted in the stromata of the evaluated *Pyrenopolyporus* spp.

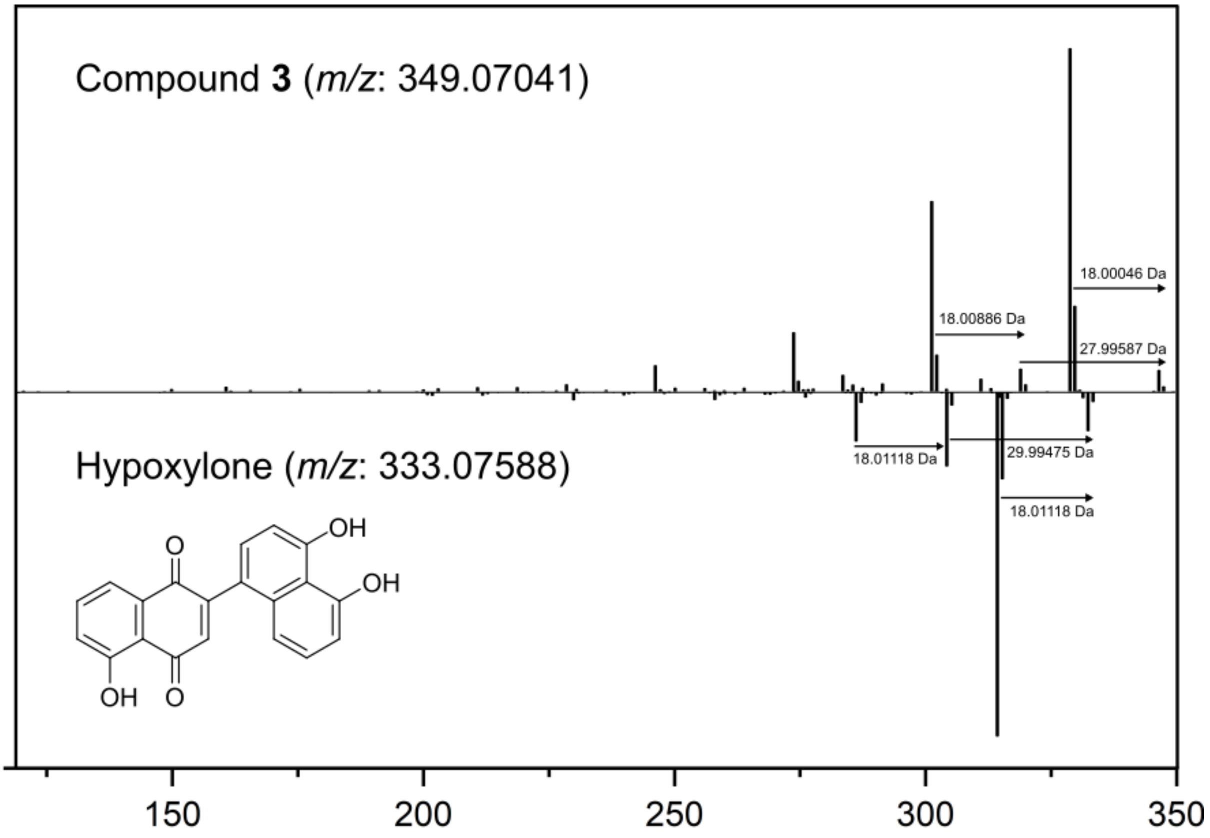

Figure S8. MS/MS spectra comparison between hypoxylone (**1**) and compound **3**. The MS/MS similarity score between the two metabolites is < 500, but both spectra share analogous neutral losses for the major fragment ions.
